# Pushing C–N bond formation in water: sustainable strategies, mechanistic roles and future perspectives

**DOI:** 10.1039/d6ra06057a

**Published:** 2026-09-28

**Authors:** Muhammad Adnan Bashir, Xue Shi, Rui Ding, Chun-Hong Hu, Shuangshuang Wang, Xiong-Zhenfang, Xunbo Lu

**Affiliations:** a School of Nursing and Health Management (School of Life Sciences and Chemistry), Wuhan Donghu University Wuhan 430200 China qureshi.chemist@gmail.com; b School of Chemical Biology and Biotechnology, Shenzhen Graduate School of Peking University Shenzhen 518055 China; c School of Chemistry and Chemical Engineering, Henan University Henan China; d School of Chemistry and Chemical Engineering, Laboratory of Marine Green Fine Chemicals, Lingnan Normal University (LNU) Zhanjiang Guangdong 524048 China Luxunbo@foxmail.com

## Abstract

C–N bonds are fundamental structural motifs in pharmaceuticals, natural products, agrochemicals, dyes, polymers, and functional organic materials. Consequently, the development of efficient and sustainable methods for C–N bond construction has become a central objective in modern synthetic chemistry. In this context, water has emerged as an attractive reaction medium owing to its abundance, low toxicity, environmental compatibility, unique hydrogen-bonding ability, and capacity to promote distinct reactivity under both homogeneous and heterogeneous conditions. Nevertheless, the use of water in C–N bond formation offers more than solvent replacement; it represents a mechanistically distinctive approach in which aqueous environments actively reshape reaction pathways. This review summarizes recent progress in C–N bond formation and covers both “on water” and “in water” phenomena, with particular emphasis on current achievements, mechanistic understanding, and future opportunities. The discussed transformations are organized according to reaction strategy, including transition-metal-free processes, transition-metal-catalysed systems, and emerging electrochemical and photochemical approaches. Particular emphasis is placed on how water changes reaction pathways through hydrogen bonding, interfacial effects, proton transfer, hydrophobic aggregation, and catalyst stabilization. By integrating synthetic scope with mechanistic insights, this review provides a coherent framework for the rational design of sustainable aqueous C–N bond-forming reactions.

## Abstract 2

This review summarizes recent advances in C–N bond formation in water, covering “on-water” and “in water” processes. It highlights the mechanistic roles of water, including hydrogen bonding, proton transfer and catalyst stabilization.

## Graphical abstract

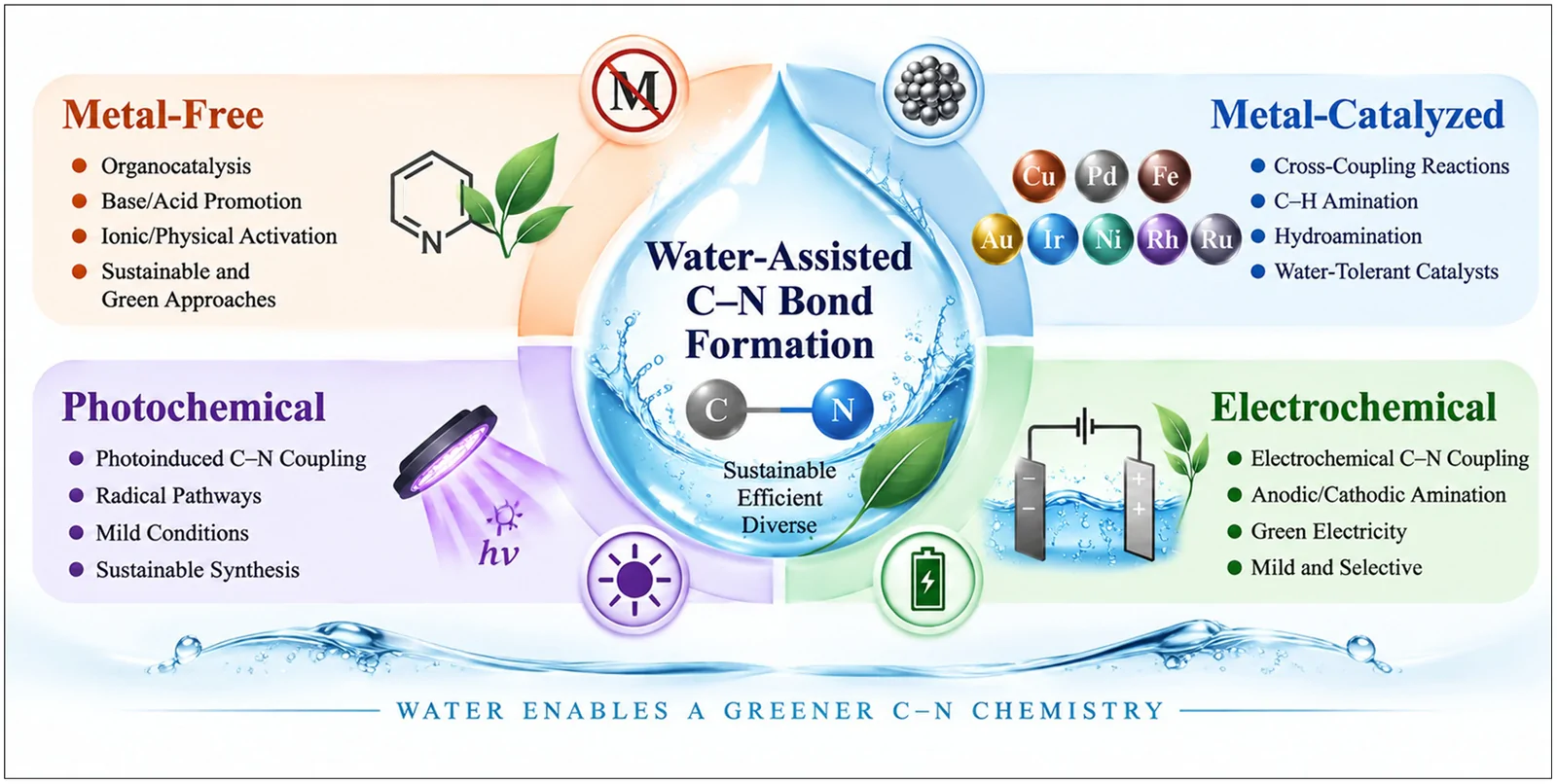

## 1 Introduction

The construction of C–N bonds represents one of the most fundamental transformations in modern synthetic chemistry because nitrogen-containing molecules are central to pharmaceuticals, natural products, agrochemicals, dyes, polymers, and functional materials.^1–5^ Amines, amides, heterocycles, amino acids, ureas, and related nitrogen-containing frameworks constitute key structural units in medicinal chemistry and fine-chemical manufacturing.^5–8^ In pharmaceutical synthesis in particular, C–N bond-forming reactions are among the most frequently used transformations, reflecting their central role in the preparation and diversification of bioactive molecules.^9–12^ Despite this importance, the selective formation of C–N bonds remains challenging because carbon and nitrogen differ substantially in electronegativity, nucleophilicity, basicity, and coordination behavior.^13^ These features often lead to competing pathways, catalyst inhibition, and poor chemoselectivity, or they necessitate activated substrates, strong bases, transition-metal catalysts, and organic solvents. Conventional C–N bond-forming methodologies have historically relied on energy-intensive conditions, prefunctionalised coupling partners, toxic reagents, and large quantities of organic solvents.^14,15^ Such limitations are increasingly incompatible with the principles of green and sustainable chemistry, particularly in pharmaceutical and fine-chemical synthesis, where solvent waste, metal contamination, and process safety are critical concerns. Therefore, the development of milder, cleaner, and more resource-efficient C–N bond-forming strategies has become an urgent objective. In this context, water has emerged as a highly attractive reaction medium because it is abundant, inexpensive, non-flammable, environmentally benign, and uniquely capable of participating in hydrogen bonding, proton transfer, hydrophobic aggregation, and interfacial activation (Fig. 1A).

**Fig. 1:**
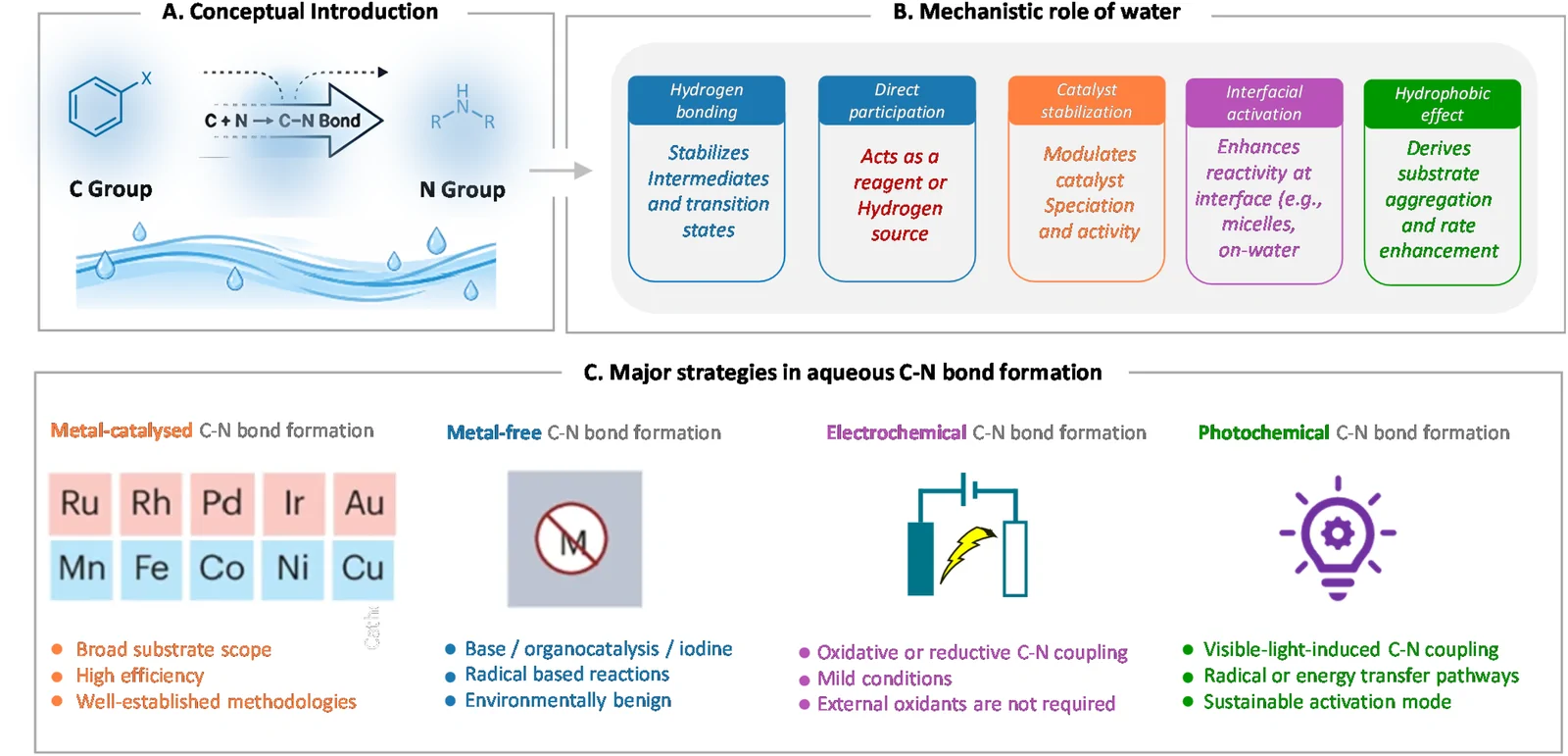
Schematic overview of C–N bond formation in water: (A) conceptual introduction of aqueous C–N bond construction; (B) mechanistic roles of water in C–N bond-forming reactions; and (C) major strategies for aqueous C–N bond formation, including metal-catalysed, transition-metal-free, electrochemical, and photochemical approaches.

The relationship between water and C–N bond formation is, however, intrinsically complex.^16^ In this context, it is important to distinguish the terms “in water”, “on water”, and “aqueous medium”, which describe different reaction environments. Reactions performed “in water” generally involve substrates, catalysts, or reagents dissolved or dispersed within water as the reaction medium, where water directly influences solvation, hydrogen bonding, and proton-transfer processes.^16,17^ In contrast, “on water” reactions typically involve water-insoluble organic substrates and occur at the water–organic interface, where interfacial effects, hydrophobic interactions, and substrate organization may contribute to enhanced reactivity.^18–21^ The broader term “aqueous medium” refers to reaction systems in which water serves as the predominant solvent or reaction environment, including homogeneous, heterogeneous, micellar, and biphasic conditions.^19,22^ Recognizing these distinctions is essential because the contribution of water to C–N bond formation depends strongly on the physical and chemical characteristics of the reaction system (Fig. 1B).

On the one hand, water has long been considered problematic in organic synthesis because many substrates and catalysts show poor aqueous solubility, and key intermediates, such as imines, iminium ions, organometallic species, and activated electrophiles, may undergo hydrolysis or catalyst deactivation.^23^ In metal-catalysed reactions, water can also compete for coordination sites or alter catalyst speciation. The recent advances have demonstrated that water can be transformed from an apparent limitation into a powerful enabling medium.^1^ Depending on the reaction system, water can function as a green solvent, hydrogen-bond donor or acceptor, proton shuttle, phase-transfer-supporting medium, micellar or interfacial reaction platform, stabilizer of polar transition states, hydrogen source, or even a direct reaction participant.^24^

Beyond solvent selection, the sustainability of aqueous C–N bond-forming processes should be evaluated using quantitative green chemistry metrics, including atom economy (AE),^25^ E-factor,^26^ and process mass intensity (PMI).^27^ Although the use of water can substantially reduce the environmental burden associated with volatile organic solvents, the overall sustainability of a reaction also depends on catalyst loading, stoichiometric reagents, additives, reaction concentration, energy input, and purification procedures. High atom economy transformations that minimize by-product formation, together with low E-factor and PMI values resulting from reduced solvent and waste generation, represent important targets for future aqueous C–N bond-forming methodologies.^28^ Therefore, water should be considered as one component of a broad sustainability framework rather than the sole criterion for evaluating green synthetic processes.

From a green chemistry perspective, water represents the ideal solvent, nature's own reaction medium.^29–31^ As Lipshutz has compellingly argued, “making the switch to water, nature's chosen reaction medium, akin to that in which biocatalysis is typically performed is inevitable”.^32^ The inertness of water toward many organic functional groups, combined with its unique ability to participate in hydrogen bonding and proton transfer, can facilitate certain reaction pathways.

Recent advances in nanocatalysis have further expanded the possibilities of sustainable C–N bond formation by providing catalysts with unique nanoscale properties.^33^ Compared with conventional bulk catalysts, nanocatalysts offer high surface-to-volume ratios, increased exposure of active sites, tunable electronic structures, and improved catalyst–substrate interactions, which can enhance catalytic efficiency and selectivity.^34–36^ In aqueous systems, nanoscale catalysts may also facilitate mass transfer, stabilize reactive intermediates, and enable efficient catalyst recovery and recycling, making them attractive platforms for green C–N bond-forming transformations.

Although several insightful reviews have summarized the progress in C–N bond formation, few have focused specifically on Pd-^37–39^ or Cu-^40–43^ based systems, and even fewer have done so in the context of water as a reaction medium. Therefore, a dedicated, systematic and comprehensive review focusing on C–N bond formation in water is both timely and necessary. This review summarizes recent progress in C–N bond formation in water, with particular emphasis on synthetic development and the mechanistic roles of water. Unlike previous reviews focusing mainly on specific catalytic platforms or general aqueous organic synthesis, this review highlights how water actively influences C–N bond-forming processes through hydrogen bonding, interfacial effects, proton transfer, hydrophobic aggregation, substrate organization, and catalyst stabilization. The discussion is organized into transition-metal-free approaches, including iodine-mediated, dual-activation, microwave-assisted, organocatalytic, micellar, ionic-liquid-assisted, and microdroplet-mediated systems, followed by transition-metal-catalysed reactions involving Cu, Pd, Rh, Fe, Ni, Au, and related catalysts. Emerging electrochemical and photochemical C–N bond-forming reactions in aqueous media are also discussed (Fig. 1C). This review primarily covers literature published between 2010 and 2025, with a particular focus on developments from the past decade, while also including selected seminal earlier contributions that established foundational concepts in aqueous C–N bond formation. Biocatalytic C–N bond formation, although an important and rapidly developing area, is beyond the scope of this review; instead, we focus on chemical approaches. By integrating reaction scope with mechanistic understanding, this review provides a coherent framework for designing sustainable, selective, and scalable aqueous C–N bond-forming processes.

## 2 Metal-free C–N bond formation

C–N bond formation without the use of any metal catalyst is of particular interest, especially when carried out in water as a green reaction medium.^44^ It is noteworthy that some protocols can efficiently promote such transformations even under mild reaction conditions.^45^ In this section, we have selected several interesting reports that highlight the potential of water as a sustainable solvent for transition-metal-free C–N bond formation.

### 2.1 Iodine-catalysed C–N bond formation in water

Iodine-mediated C–N bond formation in water represents an important transition-metal-free strategy for sustainable synthesis,^46^ because iodine species can function as mild oxidants, Lewis acids, redox mediators, or precursors to hypervalent iodine reagents under aqueous conditions.^46–48^ In these transformations, water influences catalyst speciation, stabilizes polar or charged intermediates, promotes proton transfer, and, in selected cases, participates directly in the generation of reactive iodine species.^49^ Therefore, this section focuses on iodine-catalysed or iodine-mediated C–N bond-forming reactions performed in water or aqueous media, with particular attention to the mechanistic contribution of water.

An early representative example by Das and co-workers disclosed an unprecedented *in situ*-generated iodine(iii)-catalysed oxidative C–N bond-forming cyclization for the synthesis of benzimidazole-fused heterocycles from readily accessible *N*-aryl-2-amino-N-heterocycles. Notably, water participates directly in the generation of the active catalyst, the hypervalent iodine(iii) species (Koser's reagent (PhI(OH)OTs)). Nevertheless, this protocol has notable limitations, as it relies on stoichiometric acid and an external oxidant (*m*-CPBA), which restrict functional-group tolerance and practical scalability. Moreover, the method is largely confined to pre-organized *N*-aryl heterocycles, thereby limiting its generalizability (Scheme 1).^50^

**Scheme 1:**
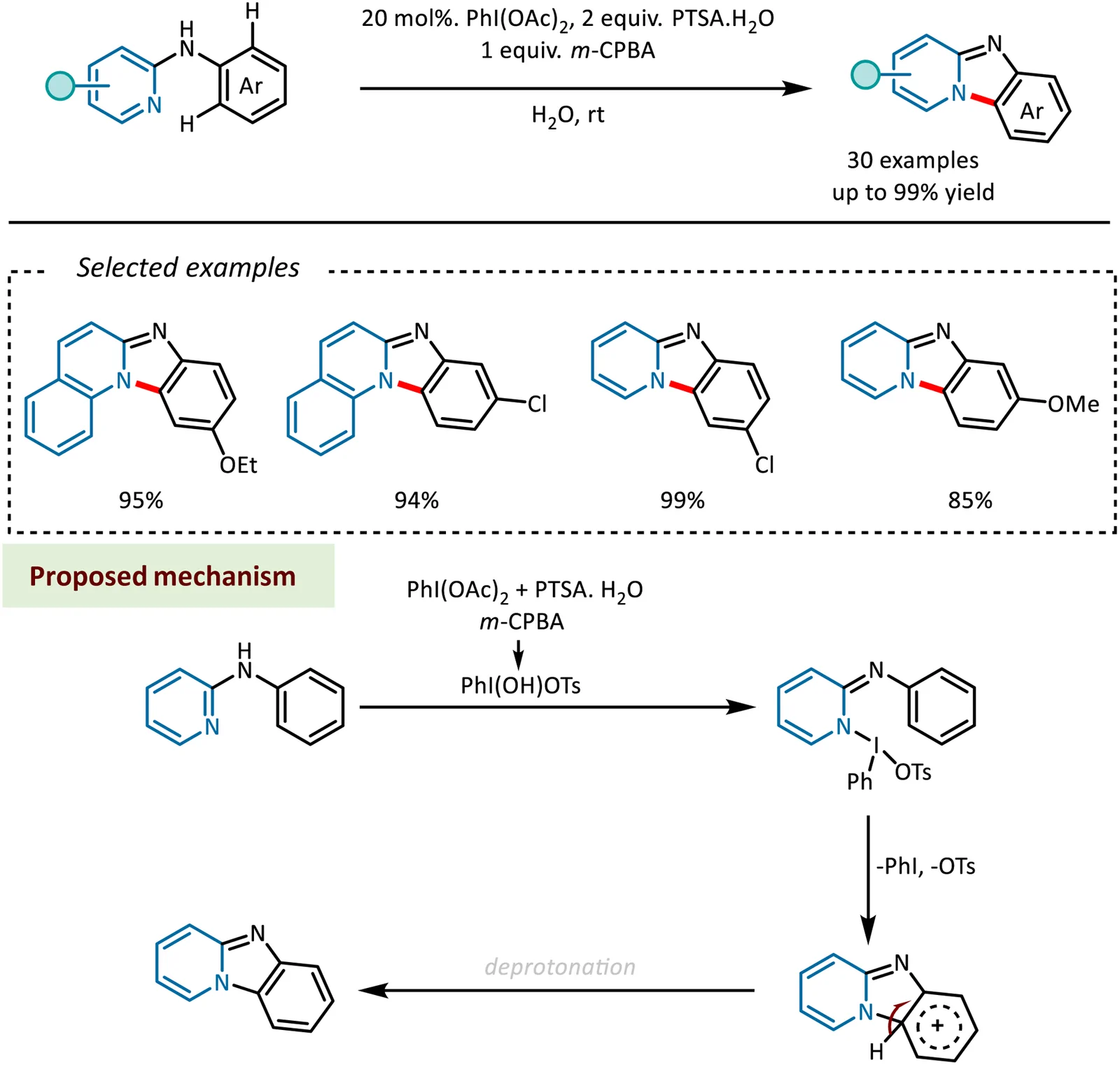
Synthesis of benzimidazole-fused heterocycles.

In contrast to the hypervalent iodine(iii)-catalysed system requiring a stoichiometric external oxidant, Narender and colleagues reported in the same year an operationally simpler approach using molecular I_2_ as a direct mediator in water as the sole solvent. This method enables oxidative C–N/C–O bond formation for the synthesis of polysubstituted oxazoles from readily available β-ketoesters and primary amines. Notably, it was proposed that water reacted with molecular iodine under basic conditions to generate hypoiodous acid (HIO), which subsequently disproportionated to produce oxygen (O_2_), acting as the terminal oxidant for the aromatization step (Scheme 2).^51^ The authors demonstrated that the β-keto esters having aliphatic substituents afforded the oxazoles in better yield than those with the aromatic groups. The proposed mechanism involved the initial substitution of iodine with benzylamine to produce an intermediate, which underwent intramolecular cyclization and oxidation to afford the final aromatised oxazole.

**Scheme 2:**
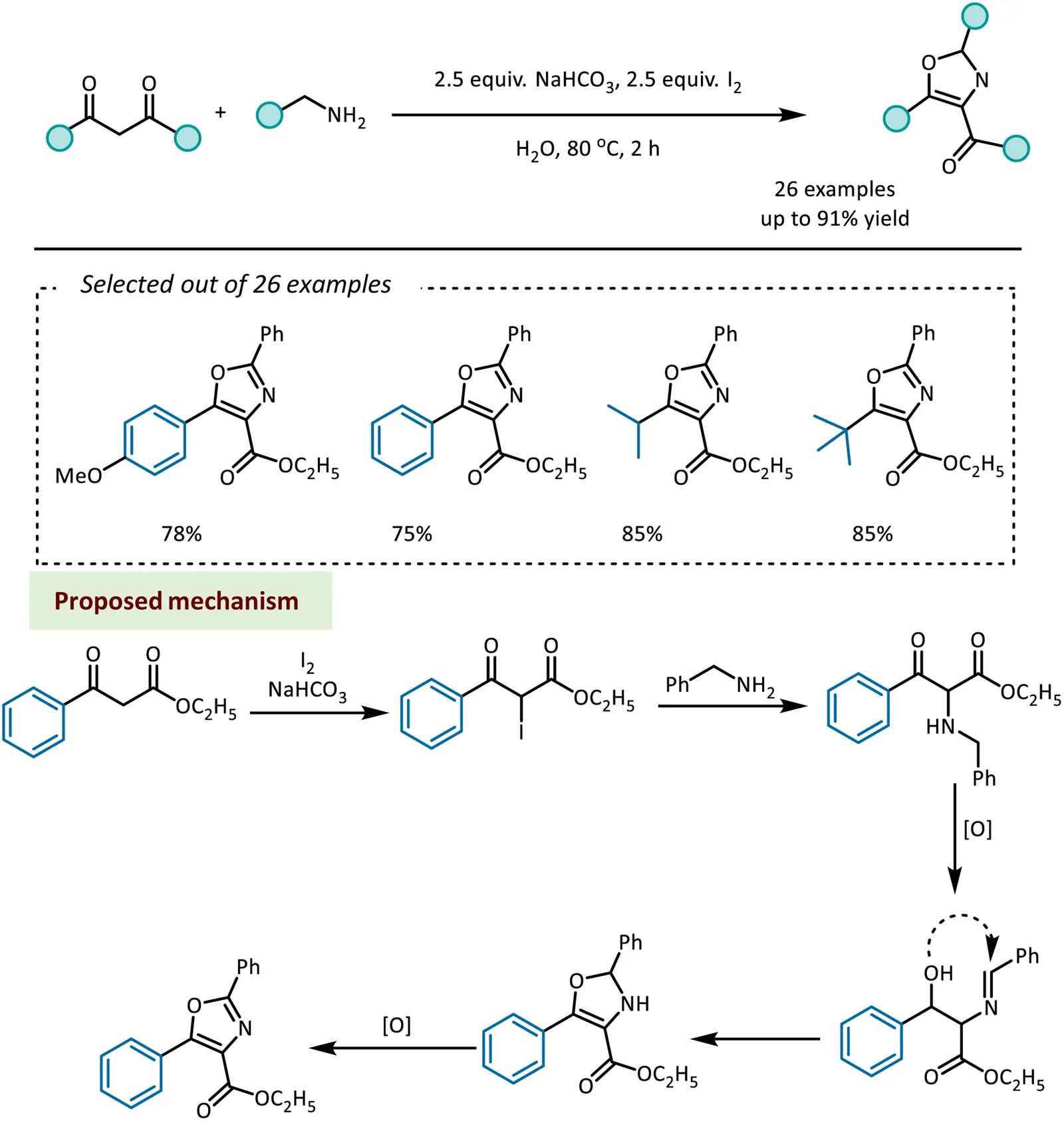
Synthesis of polysubstituted oxazoles.

Extending the utility of molecular iodine in aqueous oxidative heterocyclization, Nakka and co-workers demonstrated that isothiocyanates could serve as versatile precursors for the construction of 1,2,4-triazole and 1,2,4-thiadiazole scaffolds through oxidative C–N and N–S bond formation in water. In this system, water likely functions as a protic medium capable of stabilizing polar intermediates, including thioamide- and hydrazone-type species, thereby facilitating cyclization and improving product formation. The practical value of the method was illustrated by the efficient synthesis of 3-substituted 5-amino-1,2,4-thiadiazoles.^52^

Further progress in I_2_-mediated oxidative C–N bond formation was made by Banerji and co-workers, who reported an aerobic oxidative C(sp^3^)–H functionalization of primary amines for the regioselective synthesis of polysubstituted oxazoles in water. This peroxide-free protocol is attractive from a green chemistry perspective and is further demonstrated through the synthesis of the natural product, texaline. In addition, the resulting polyarylated oxazoles displayed fluorescence properties, highlighting the potential connection between aqueous C–N bond-forming chemistry and functional materials.^53^

In a related multicomponent strategy, Singh and co-workers used molecular iodine as a mild Lewis acid catalyst in water for the one-pot synthesis of highly functionalized pyrazoles from phenylhydrazine, malononitrile, and aldehydes. Here, iodine activates the aldehyde carbonyl, whereas water likely provides a polar environment that stabilizes intermediates and enables simple product isolation. These studies show that iodine/water systems can support both oxidative and Lewis acid-type pathways; however, their efficiency often depends on highly reactive substrates and favourable cyclocondensation pathways.^54^

A mechanistically distinct class of iodine-mediated reactions involves C(sp^3^)–H functionalization through redox or radical pathways. In 2023, Mukhopadhyay and co-workers reported that molecular iodine (I_2_) functioned as an efficient transition-metal-free redox catalyst for the aerobic oxidative functionalization of Cα(sp^3^)–H bonds of benzyl amines in water under air. This protocol promotes successive oxidative C–N and C–C coupling reactions through the activation of benzylic C(sp^3^)–H bonds and the *in situ* generation of imine intermediates, leading to intramolecular cyclization and the formation of 5*H*-pyrazino[2,3-*b*]indoles (Scheme 3).^55^ Importantly, unlike the iodine/water system reported by Narender and colleagues,^51^ water does not chemically react with iodine to form species such as HIO; instead, it provides a polar environment that stabilizes charged intermediates while allowing aerial oxygen to participate in the final aromatization step. Notably, mildly aqueous (moist) conditions provide optimal yields, whereas solvent-free or excess water conditions significantly diminish reactivity.

**Scheme 3:**
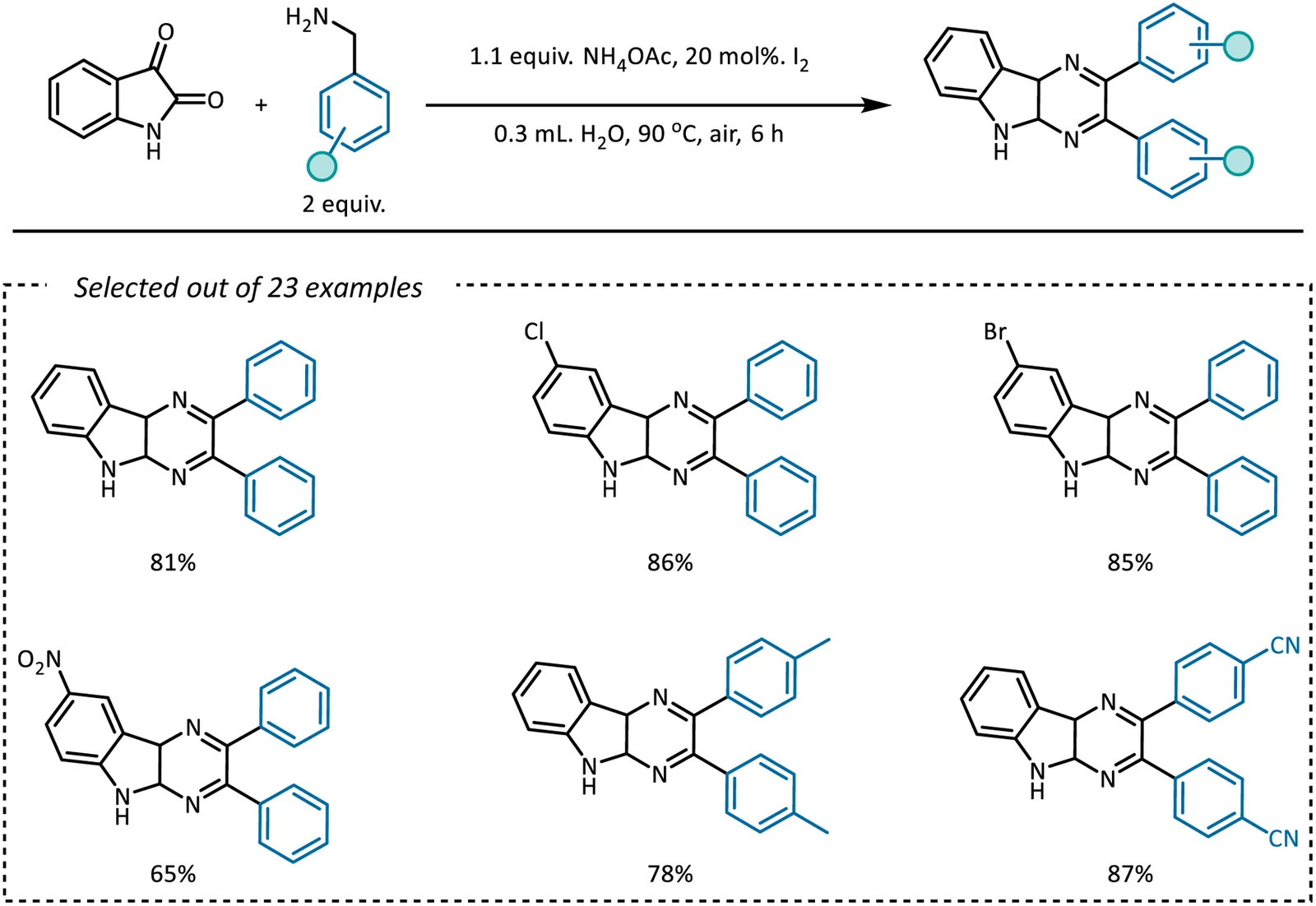
Synthesis of 5*H*-pyrazino[2,3-*b*]indoles.

Punniyamurthy and co-workers further expanded iodine-based aqueous C–N bond formation by employing tetrabutylammonium iodide and *tert*-butyl hydroperoxide for the synthesis of functionalized oxazolidines and imidazolidines through C–O and C–N bond formation. In contrast to molecular-I_2_-mediated oxidative cyclization, this reaction proceeds through a radical pathway, in which the oxidation of iodide generates iodine species and *tert*-butoxyl radicals. These radicals initiate C–H bond cleavage to form an iminium intermediate, followed by intramolecular cyclization to furnish the heterocyclic products.

Although this strategy broadens the mechanistic diversity of iodine-mediated C–N bond formation, the use of *tert*-butyl hydroperoxide and radical intermediates may complicate selectivity control, especially for more complex or multifunctional substrates (Scheme 4).^56^ Hypervalent iodine chemistry in aqueous media has also enabled transformations in which water likely acts as a direct reactant. Zhao and co-workers reported a PIFA-mediated cyclization of *ortho*-(1-alkynyl)benzamides to afford 3-hydroxy-2,3-dihydroisoquinoline-1,4-diones. Here, water plays a more complex role than that of a simple co-solvent, as it serves as the oxygen source for the hydroxyl group at the C-3 position of the product. Nevertheless, the reaction was inefficient in dry acetonitrile, whereas the addition of water markedly improved product formation, confirming its essential mechanistic role (Scheme 5).^57^

**Scheme 4:**
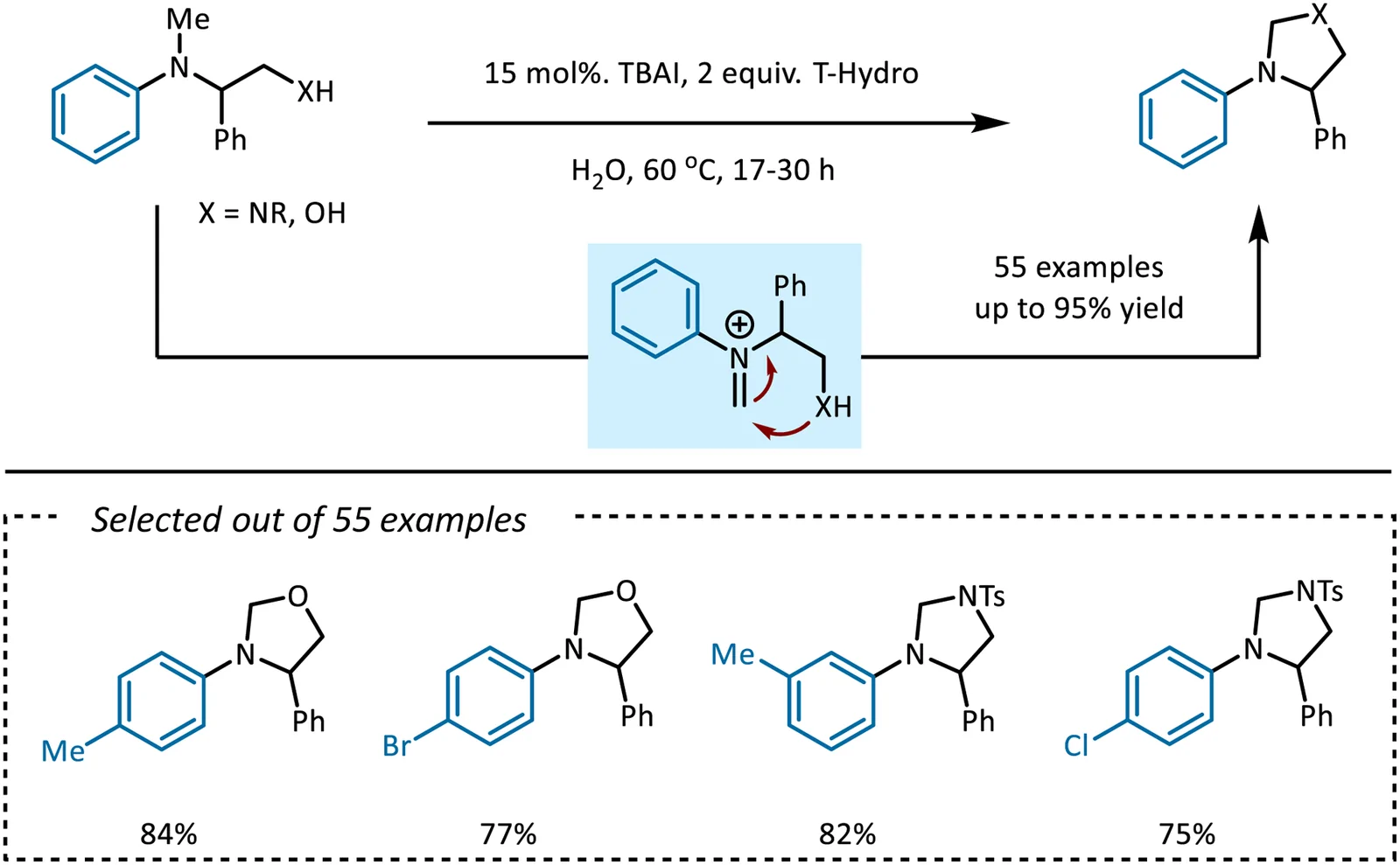
Synthesis of functionalized oxazolidines and imidazolidines.

**Scheme 5:**
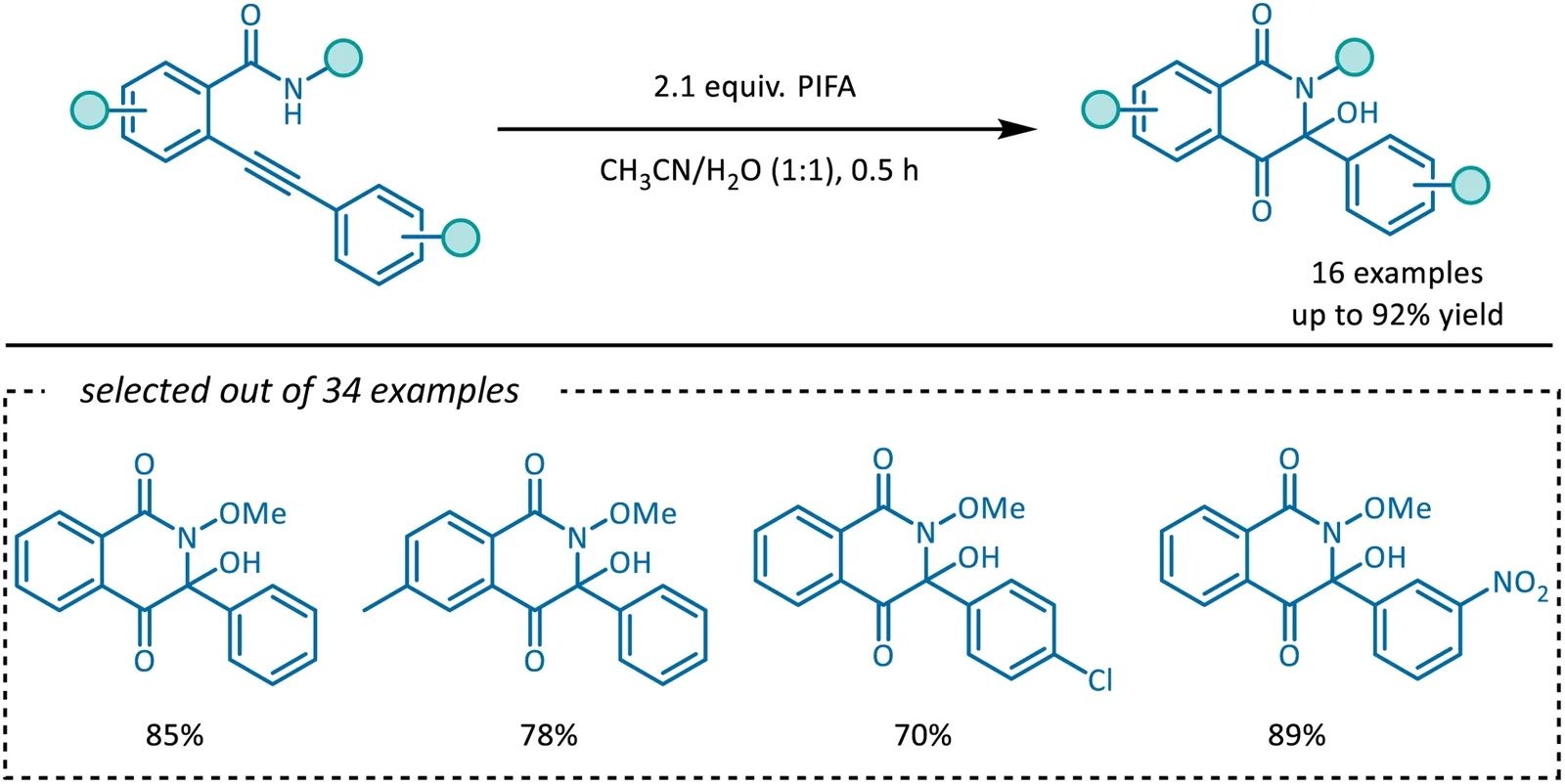
Synthesis of 3-hydroxy-2,3-dihydroisoquinoline-1,4-dione.

Extending the aqueous C–N bond-forming strategy to the realm of peptide chemistry, Hanaya and co-workers engineered a selective C-2 functionalization of tryptophan in non-protected peptides. Their approach leverages *in situ*-generated iodine(iii) species and simple azoles under acidic aqueous conditions, bypassing the need for protective groups and preserving the native secondary structure of the peptide. The methodological breakthrough lies in an acidic solvent system (DMSO/H_2_O = 1 : 9, pH ≈ 1) composed of KI, KIO_3_, and methanesulfonic acid. Notably, water is far more than a passive medium. It actively enables the dissolution and handling of biologically relevant peptides without denaturation, while its precise acidity governs the reactive iodine equilibrium, suppresses undesired side reactions (*e.g.*, tyrosine iodination), and fine-tunes the nucleophilicity of azoles, which are all critical for achieving high tryptophan selectivity.^58^

Overall, iodine-mediated C–N bond formation in water has evolved from the oxidative heterocyclization of activated substrates to more sophisticated transformations involving C(sp^3^)–H functionalization, radical pathways, water-assisted hypervalent iodine chemistry, and peptide modification. Across these examples, water plays multiple roles, including solvent, iodine-speciation medium, stabilizer of polar intermediates, proton-transfer medium, interfacial promoter, and, in some cases, direct reactant. Nevertheless, several limitations remain. Many protocols still require stoichiometric iodine reagents, external oxidants, strong acids, or peroxide additives, which compromise atom economy and operational simplicity. In addition, substrate scope is often restricted to preorganized or highly activated systems, and radical or single-electron-transfer pathways can make selectivity difficult to control. Therefore, future progress should focus on truly catalytic iodine cycles using benign terminal oxidants, such as air, oxygen, or hydrogen peroxide, or *via* electrochemical oxidation, as well as on expanding aqueous iodine-mediated C–N bond formation to complex, pharmaceutical, and asymmetric synthesis.

### 2.2 Water-enabled C–N bond formation through dual activation

The ability of water to promote C–N bond formation through hydrogen-bond-mediated dual activation represents a distinctive concept in aqueous organic synthesis. Various experiments indicate that hydrogen bonding contributes, to a certain degree, to the rate enhancements of pericyclic reactions in water, as well as the hydrophobic effect.^59^ Unlike conventional solvent effects, this mode of activation relies on the simultaneous organization of both reaction partners by water molecules. In such systems, water can activate the electrophile through hydrogen bonding while assisting the nucleophile through complementary hydrogen-bond interactions, thereby lowering the energetic barrier for C–N bond formation without the need for classical catalysts or toxic organic solvents.^38–41^

Chakraborti and co-workers first established unprecedented water-enabled dual activation through the catalyst-free *N-tert*-butyloxycarbonylation of amines under ambient conditions, where water was proposed to activate both (Boc)_2_O and the amine through cooperative hydrogen bonding.^60^ This concept was later extended to the “on-water” synthesis of benzothiazoles and benzothiazolines from aldehydes and 2-aminothiophenols, in which water likely promoted condensation and cyclization by simultaneously engaging the aldehyde carbonyl and thiol/amine functionalities.^61^ These studies demonstrate that water can function as an active amphiphilic promoter, enabling C–N bond formation without external acid, base, or conventional catalyst.

The further development of this strategy led to an “all-water” tandem protocol for the synthesis of *N*-arylmethyl-2-substituted benzimidazoles. Notably, water plays a more complex mechanistic role beyond simple hydrogen-bond activation. A water dimer was proposed to activate both the nucleophilic amino group of *ortho*-nitroaniline and the electrophilic benzylic bromide, thereby facilitating the initial *N*-alkylation step. In addition, water may also assist the subsequent nitro reduction by solvating In^3+^ species and promote the final cyclocondensation step through related hydrogen-bond-mediated activation. The comparable performance of tap water and ultrapure water further underscores the practical value of this aqueous protocol (Scheme 6).^62^

**Scheme 6:**
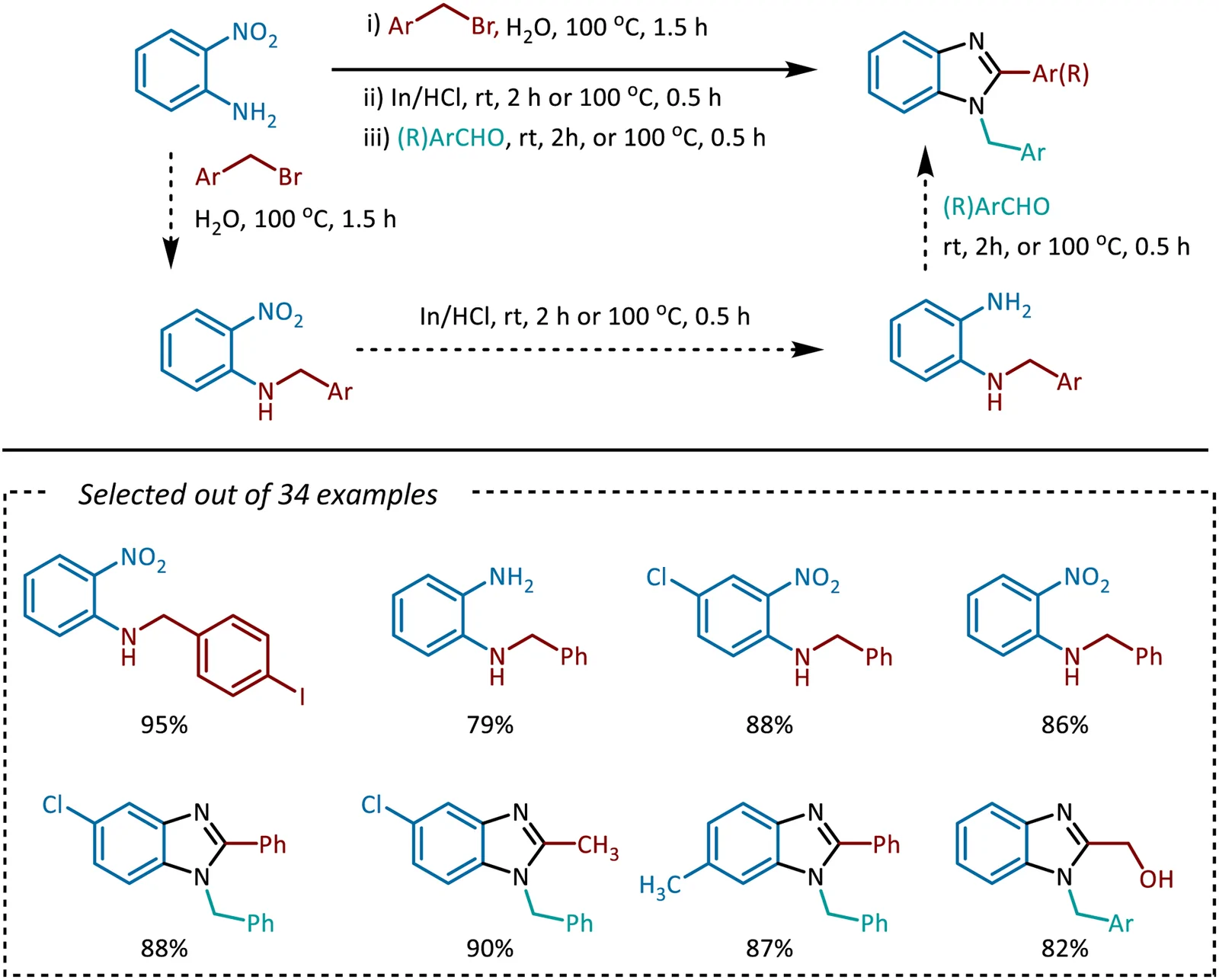
Synthesis of *N*-arylmethyl-2-substituted benzimidazoles.

Within the same mechanistic philosophy, Chakraborti and co-workers also reported a diversity-oriented synthesis of regiodefined 1,2-disubstituted benzimidazoles in water. This transformation proceeds through synergistic electrophile–nucleophile dual activation, in which one water molecule hydrogen-bonds with the electrophilic fluoronitrobenzene to increase the susceptibility of the carbon center to nucleophilic attack, while another water molecule stabilizes and organizes the amine nucleophile. This study provides a clear mechanistic illustration of how water can actively control substrate activation and reaction selectivity through a hydrogen-bonding network (Scheme 7).^63^

**Scheme 7:**
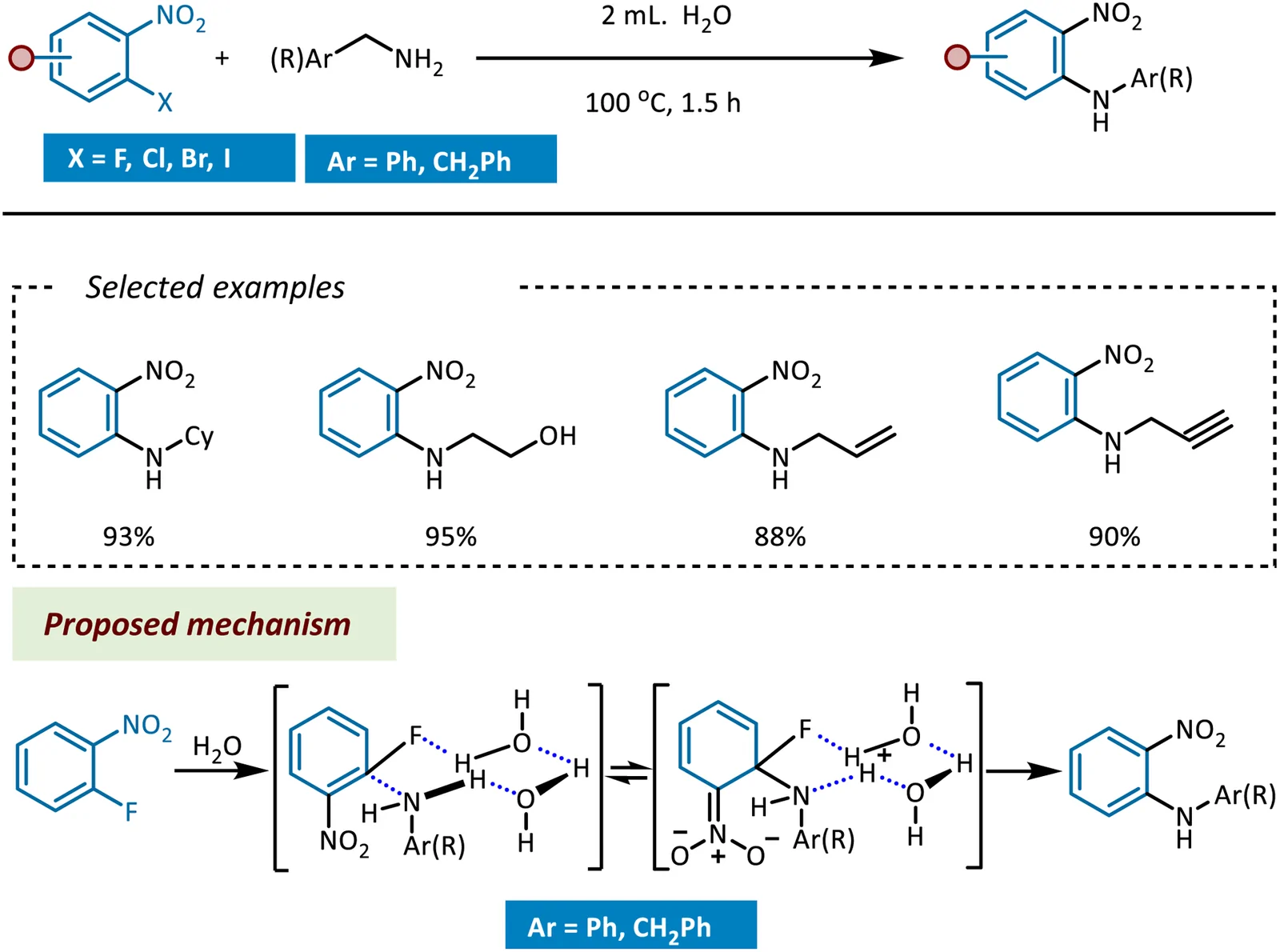
Hydrogen-bond-driven synergistic electrophile–nucleophile dual activation by water.

Despite these advances, the synthesis of 1,2-disubstituted benzimidazoles remains limited to benzyl-type *N*-alkylation and is highly dependent on halogen type, hydrogen-bond interactions, and precise reaction conditions. Moreover, the reduction step requires specific acidic conditions, which restrict substrate diversity and operational flexibility. This dual-activation concept provides a catalyst-free, environmentally benign platform for constructing diverse N-containing heterocycles under mild aqueous or “on-water” conditions. However, the further expansion of substrate scope, mechanistic generality, and operational flexibility is needed to transform this strategy into a broadly applicable synthetic tool.

### 2.3 Microwave-assisted C–N bond formation in water

Microwave-assisted synthesis in water has become an attractive strategy for sustainable C–N bond formation because it combines rapid energy transfer with the environmental advantages of an aqueous medium. Owing to its high dielectric constant, water efficiently absorbs microwave irradiation and converts it into heat, enabling the fast and relatively homogeneous heating of reaction mixtures. It is worth mentioning that in addition to serving as a green solvent, water can also improve mass transfer, promote interfacial contact between hydrophobic substrates, and simplify product isolation in selected transformations.^16–18^

Wacharasindhu and co-workers demonstrated these advantages in the catalyst-free microwave-assisted amination of 2-mercaptobenzoxazoles with amines “on water” to afford 2-aminobenzoxazoles. In contrast to the hydrogen-bond-mediated dual activation described in Section 1.2,^62,63^ water mainly functions as the sole reaction medium and microwave-absorbing phase. The reaction proceeds without an added catalyst, base, or organic co-solvent, while product isolation is simplified because the crude organic product separates as a distinct upper layer on the water surface. This example highlights how microwave irradiation can enhance the practical utility of water even when its role is primarily physical rather than explicitly catalytic (Scheme 8).^64^

**Scheme 8:**
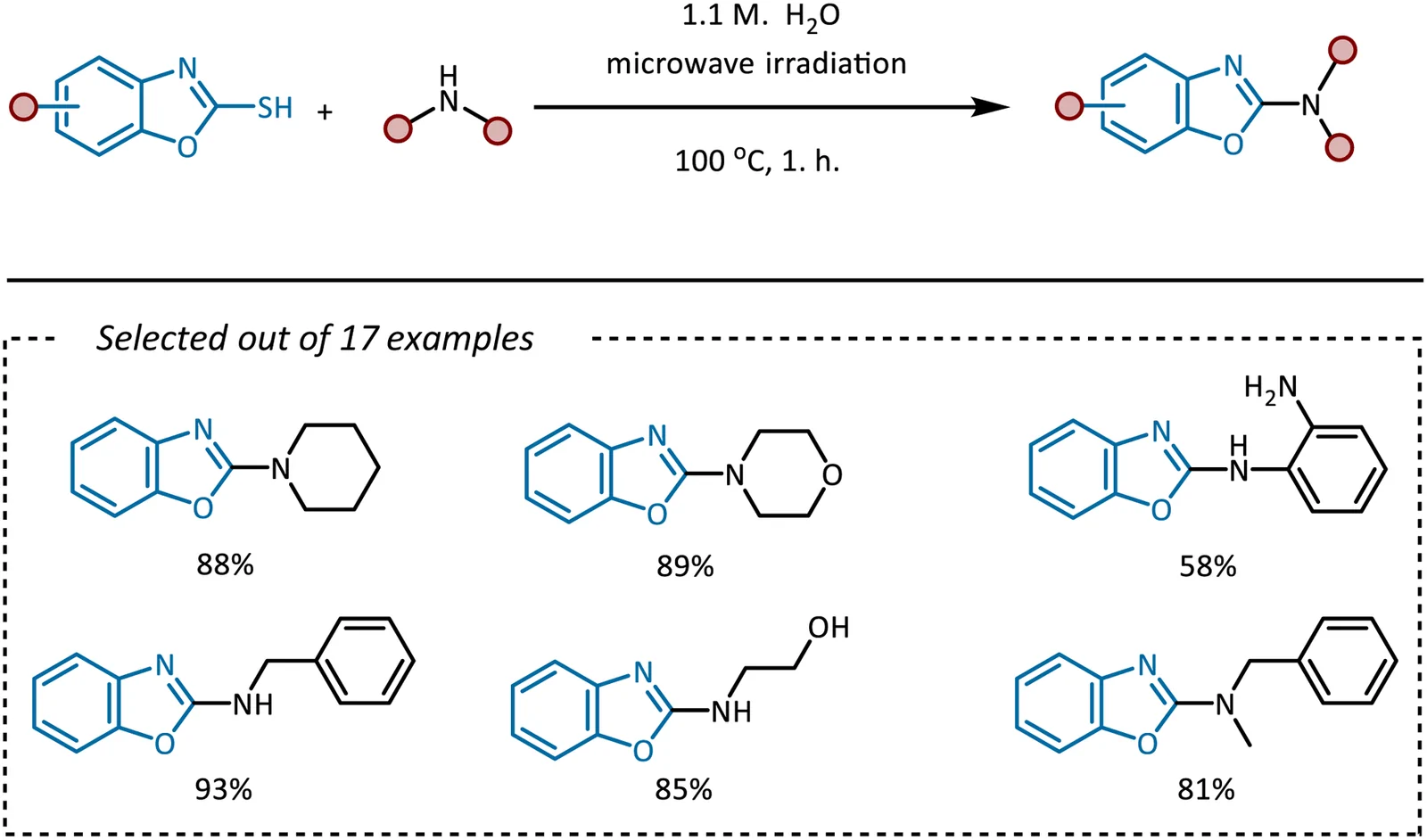
Microwave-assisted synthesis of 2-aminobenzoxazoles.

Parewa and co-workers demonstrated a more mechanistically complex microwave-assisted synthesis of quinazolin-4(3*H*)-ones in aqueous media, where pure water was insufficient because hydrophobic substrates became trapped within rigid water-organized structures. The addition of NaCl disrupted this kosmotrope-induced organization through a salting-out effect, bringing the reactants into close contact and promoting C–N bond formation. Water further assisted the process through amphiphilic dual activation, acting as both hydrogen-bond donor and acceptor toward the amine and alcohol components.^65^

Liu and co-workers further showed that microwave-assisted aqueous C–N bond formation can be enabled by phase-transfer catalysis. In their cascade cyclization/coupling reaction, tetrabutylammonium acetate (Bu_4_N^+^OAc^−^) promotes the conversion of 2-(phenylethynyl)benzoic acids and amines into 3-hydroxyisoindolin-1-ones in water. The acetate anion assists substrate activation and nucleophilic attack by the amine, whereas the tetrabutylammonium cation improves the transport and compatibility of organic reactants within the aqueous phase. This example illustrates that water can serve as an efficient platform for microwave-assisted cascade C–N bond formation when combined with suitable additives that overcome solubility and phase-contact limitations (Scheme 9).^66^ These studies demonstrate that microwave irradiation strongly amplifies the synthetic utility of water in C–N bond formation by enabling rapid heating, improved mass transfer, and clean reaction outcomes. However, substrate hydrophobicity and the frequent need for additives (salts or phase-transfer catalysts) indicate that further optimization is required to fully exploit microwave-assisted aqueous C–N bond-forming reactions.

**Scheme 9:**
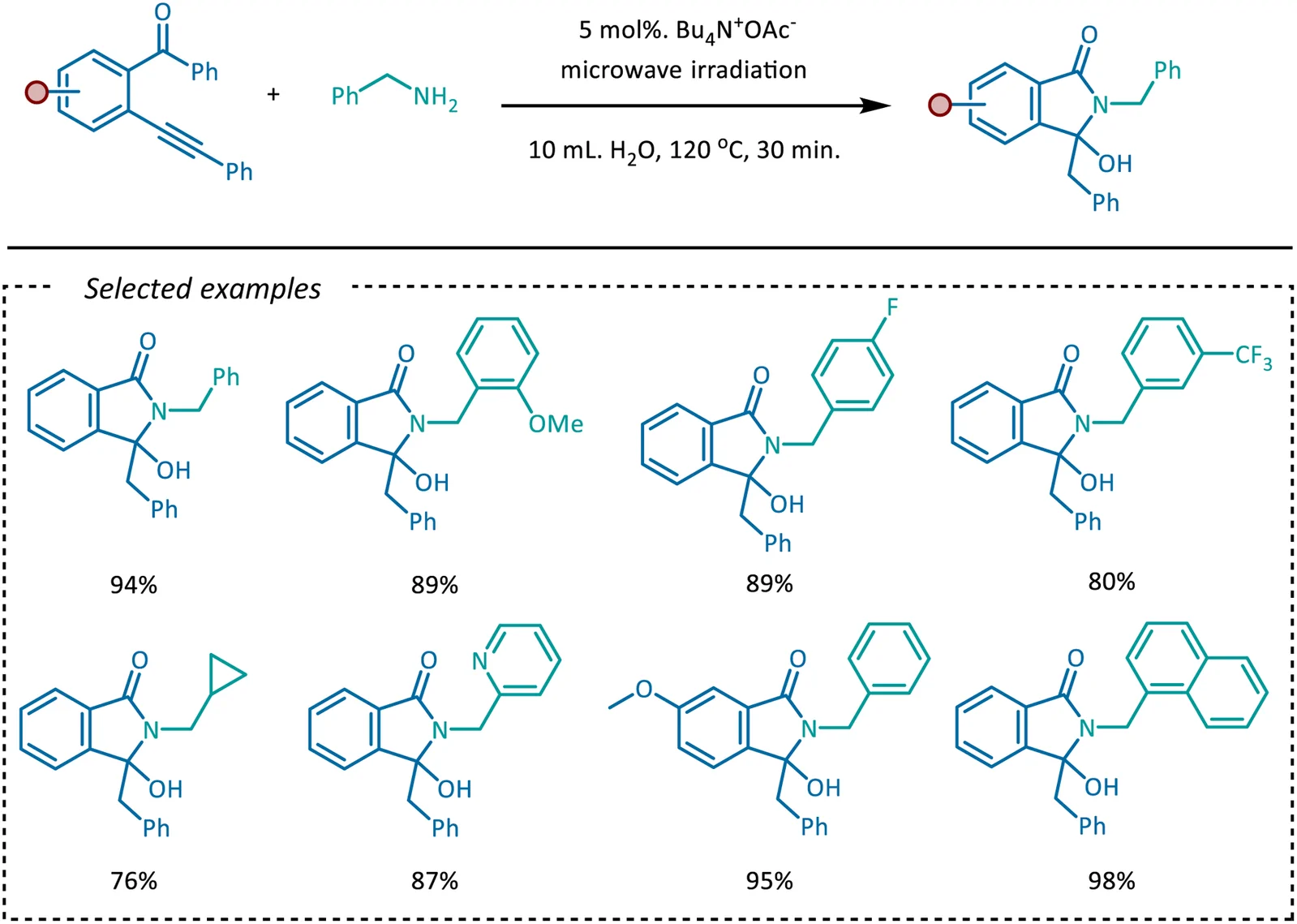
Microwave-assisted synthesis of 3-hydroxyisoindolin-1-ones.

### 2.4 Other transition-metal-free C–N bond formation approaches in water

Beyond hydrogen-bond-mediated dual activation and microwave-assisted protocols, several other transition-metal-free strategies have expanded the synthetic potential of water in C–N bond formation. These approaches demonstrate that aqueous media can promote reactions through interfacial effects, micellar organization, organocatalytic activation, ionic-liquid-assisted catalysis, solid-supported hydrophilic microenvironments, and microdroplet-mediated reactivity. Thus, water should not be viewed only as a benign solvent, but also as a dynamic reaction platform capable of organizing substrates, stabilizing polar intermediates, and accelerating otherwise difficult bond-forming events.^29–39^

A foundational contribution to this field was made by Sharpless and co-workers, who introduced the “on-water” concept by showing that many organic reactions proceed with remarkable rate acceleration when insoluble reactants are vigorously stirred in pure water as aqueous suspensions, without organic cosolvents or conventional catalysts. Although the original study focuses mainly on cycloadditions^67,68^ and Claisen rearrangements,^69^ its implications are highly relevant to C–N bond formation. For instance, the nucleophilic ring-opening of epoxides and aziridines with amines, a direct C–N bond-forming process, was shown to proceed efficiently under “on-water” conditions.^70^

Building on the “on-water” concept, Chakraborti and co-workers reported an SDOSS-assisted three-component Mannich reaction in water for the diastereoselective synthesis of functionalized tetrahydropyridines (Scheme 10).^71^ While the reaction was inefficient in pure water due to poor substrate miscibility, SDOSS formed micellar microreactors that concentrated the hydrophobic reactants at the water–surfactant interface, thereby promoting efficient C–N bond formation.

**Scheme 10:**
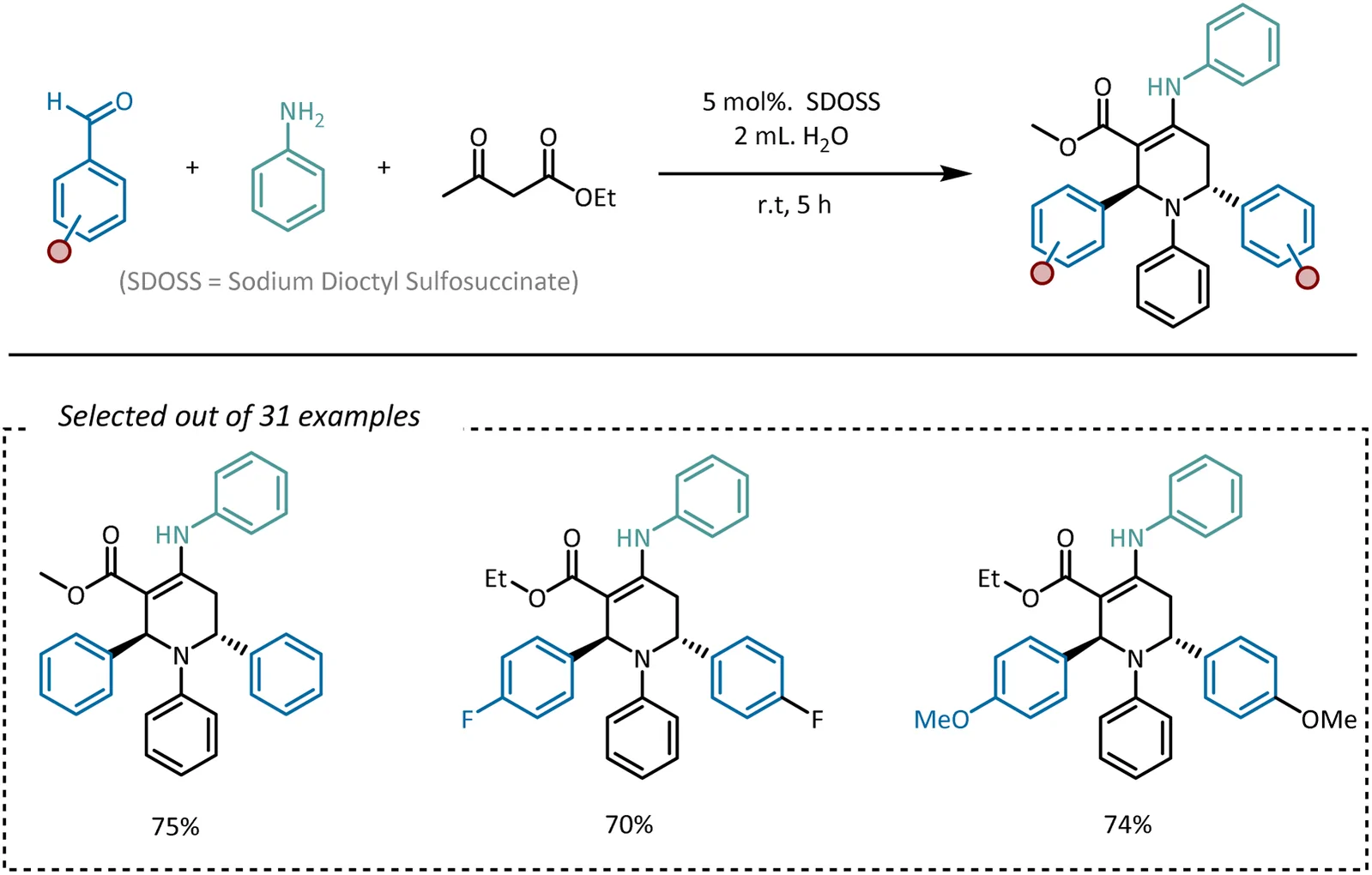
Diastereoselective synthesis of functionalized tetrahydropyridines.

Organocatalysis provides another important transition-metal-free pathway for aqueous C–N bond construction. Agarwal and co-workers reported a multicomponent synthesis of fused pyrimidines in water using l-proline as a bifunctional organocatalyst. In this transformation, l-proline promotes iminium ion formation, Knoevenagel condensation, and Michael addition, enabling consecutive C–C and C–N bond-forming steps in a single operation. Water contributes to the sustainability and operational simplicity of the process while facilitating clean product isolation (Scheme 11).^72^

**Scheme 11:**
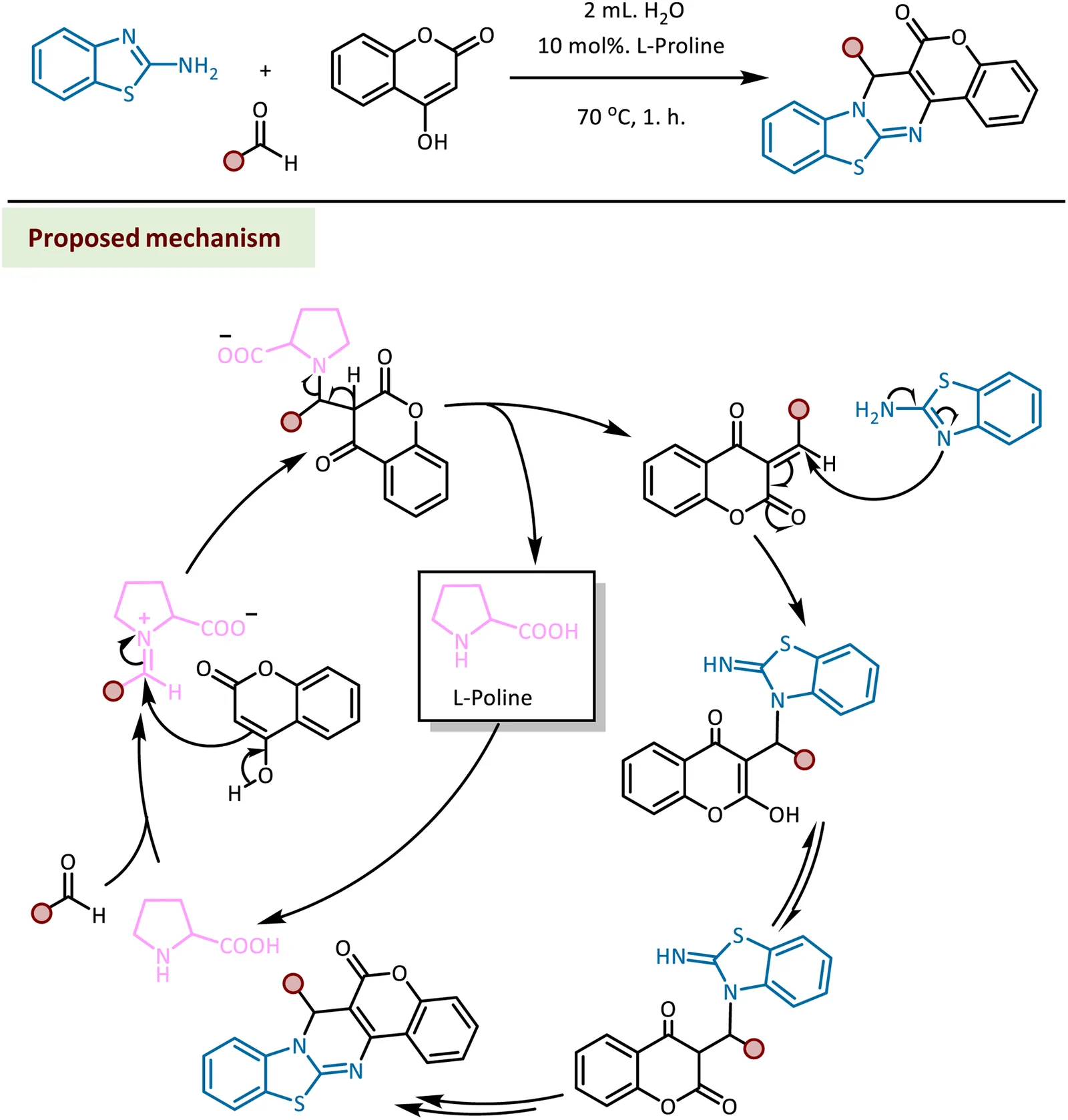
Synthesis of fused pyrimidines.

Similarly, Shao and co-workers developed a catalyst-free/base-promoted *N*-alkylation strategy using formamides as nitrogen sources in neat water under mild conditions.^73^ The method tolerates a broad substrate scope and can be performed on gram scale, highlighting the practical value of water as a simple and environmentally benign medium for C–N bond formation. Nageswar and co-workers further demonstrated that 2-chloroazoles could undergo efficient amination in water at room temperature under transition-metal-free, ligand-free, and base-free conditions.^74^ In this case, the high dielectric constant and hydrogen-bonding capacity of water likely facilitate the S_N_Ar process by stabilizing polar transition states and enhancing nucleophilic substitution. Ionic liquids and hydrophilic solid supports have also been used to improve aqueous C–N bond-forming reactions.

Deng and co-workers showed that silica gel-confined ionic liquids can promote simultaneous C=O and C=N bond transformations in water. Water enhanced catalytic efficiency relative to organic solvents by improving phase contact and participating in hydrogen-bond activation of the amine and oxime substrates.^75^ In a related strategy, Zhang and co-workers developed a hydrophilic phosphoric acid-functionalized polyacrylonitrile fiber catalyst, PANEAF, for the cyclocondensation of β-ketoesters with 2-aminobenzamides to form quinazolones. The catalyst's high density of polar –OH and –P(=O)(OH)_2_ groups, strong water uptake, and low contact angle created a water-compatible microenvironment that improved substrate interaction and afforded high yields in water.^76^

Zhang and co-workers also reported a Brønsted acidic ionic-liquid-promoted diazo coupling of naphthols with aryltriazenes for the synthesis of azo dyes in water.

The ionic liquid catalysed the diazenylation process, enabled recyclability, simplified product isolation, and allowed gram-scale synthesis with excellent yields, demonstrating the synthetic utility of ionic-liquid-assisted aqueous C–N bond formation (Scheme 12).^77^

**Scheme 12:**
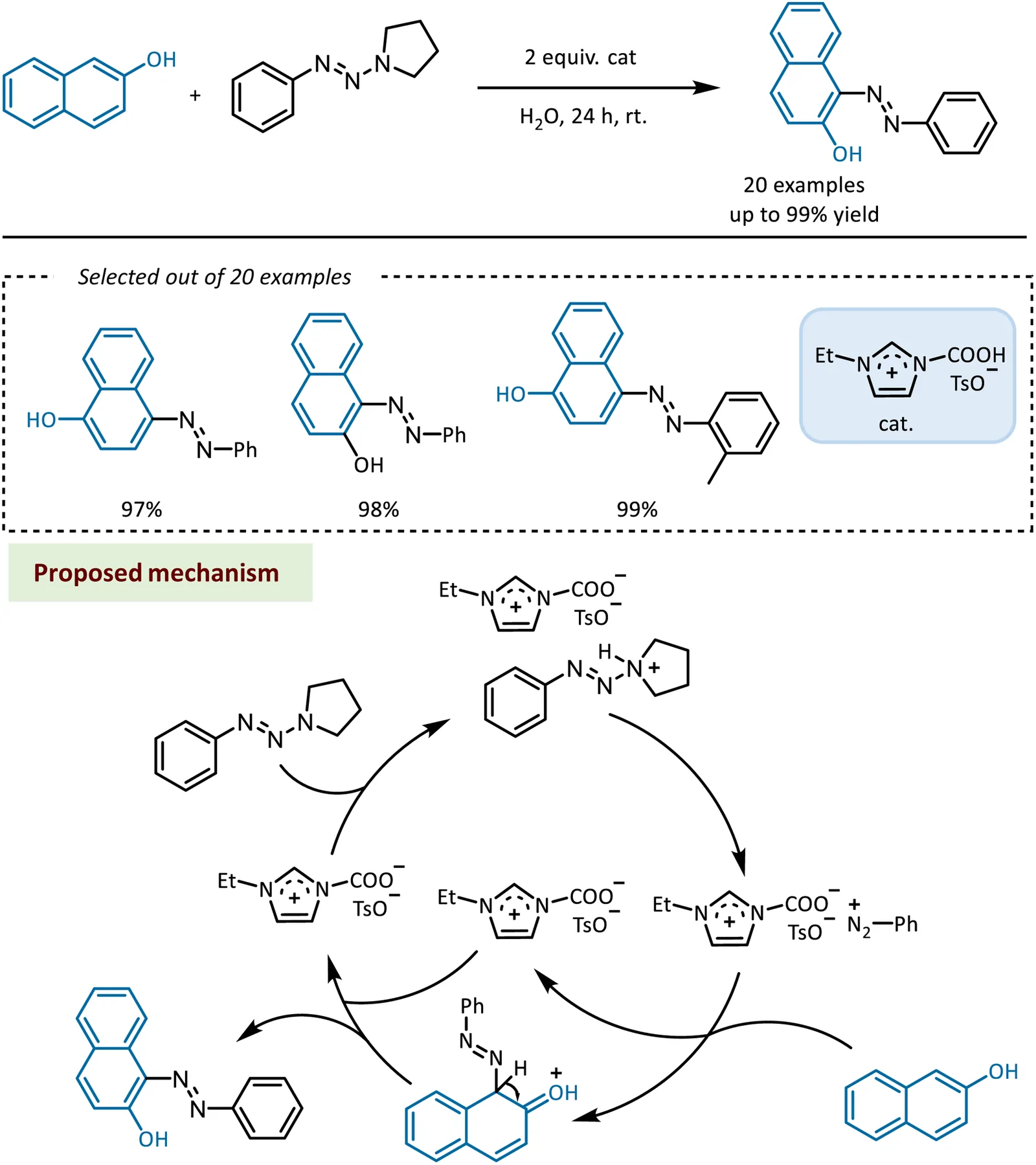
Synthesis of azo dyes *via* ionic liquid-promoted C–N bond formation.

More recently, water microdroplets have opened a distinct direction in transition-metal-free C–N bond formation. Wang and co-workers demonstrated ultrafast C–N bond formation in water microdroplets through the spontaneous generation of diarylcarbenium ions. Under electrospray conditions, the strong electric field and proton enrichment at the water–air interface create localized “super-acidic” microenvironments that promote the protonation and dehydration of diarylmethanols. The resulting carbocation intermediates rapidly react with nitrogen-containing heterocycles to give C–N coupling products under catalyst-free conditions. This study highlights that water can display reactivity in confined interfacial environments that is not observed in bulk solution.^78^

Collectively, these transition-metal-free approaches demonstrate that water promotes C–N bond formation through diverse nonclassical mechanisms, interfacial acceleration, micellar concentration, organocatalysis, ionic-liquid mediation, proton shuttling, and microdroplet-induced super-acidity. Nevertheless, several limitations remain. Many reactions still require surfactants, ionic liquids, organocatalysts, strong bases, or specialized interfacial conditions, and the generalizability of these methods is often limited to activated substrates or favourable cyclization pathways. Future progress should focus on developing broadly applicable, additive-minimized, recyclable, and mechanistically well-understood aqueous transition-metal-free systems for sustainable C–N bond formation.

## 3 Metal-catalysed C–N bond formation in water

For decades, late transition metal catalysis has been conducted under rigorously anhydrous and anaerobic conditions. This caution stems from two well-founded threats: water as a competitive ligand, blocking coordination sites,^79^ and water as a nucleophilic or electrophilic weapon, cleaving metal–carbon bonds *via* proton transfer or oxygen attack.^80,81^ However, recent advances have demonstrated that certain catalytic systems can operate efficiently in aqueous media under aerobic conditions*.* Recent studies have revealed that water can function as an effective reaction medium rather than a catalyst poison.^19^ Certain catalytic systems not only tolerate water but also exhibit enhanced activity in aqueous media under open-air conditions. Water, once considered detrimental to many catalytic systems, is now recognized as a viable and often beneficial reaction medium, enabling green and practical synthetic methodologies.

### 3.1 Cu-catalysed C–N bond formation in water

#### 3.1.1 Cu-catalysed Ullmann-type couplings

Copper-mediated Ullmann and Goldberg reactions represent some of the earliest and notable metal-mediated C–N bond-forming transformations.^82,83^ Traditionally, these reactions required high temperatures, strong bases, organic solvents, and carefully controlled conditions. Recent advances, however, have shown that appropriately designed copper catalysts, ligands, additives, and aqueous microenvironments can enable efficient C–N bond formation in water or aqueous media.^43–55^ In these systems, water acts as a green solvent, may assist base-mediated deprotonation, likely stabilizes polar Cu intermediates, promotes interfacial contact between hydrophobic substrates, or supports micellar and phase-transfer processes. Although ligand-assisted copper-catalysed C–N cross-couplings in aqueous media have been reviewed by Jianwei,^43^ the present section focuses on representative examples that clarify how water contributes to catalytic activity, selectivity, and sustainability.

Strijdonck and co-workers reported an early aqueous Cu(ii)-catalysed coupling of arylboronic acids with imidazole under mild conditions. Notably, the 1 : 1 (v/v) mixture of NMP and water fully solubilizes the binuclear µ-hydroxo Cu(ii) complex, thereby promoting the coordination of the nitrogen nucleophile (imidazole). Remarkably, this binary solvent system strikes a delicate balance; water likely facilitates the deprotonation of imidazole and may assist in transmetallation or product release, while NMP ensures substrate solubility and catalyst stability. The reaction proceeds under both air and nitrogen, indicating that external O_2_ is not required for the aryl–nitrogen bond-forming process.^84^

A different role of water was demonstrated by Teo and co-workers in a Mn/Cu bimetallic system for the Ullmann-type *N*-arylation of nitrogen heterocycles with aryl halides. Unlike the NMP/H_2_O system described above, this protocol uses water as the sole solvent and gives markedly higher yields in water than in DMF or toluene. Notably, the aqueous medium likely assists the KOH-mediated deprotonation of azoles and stabilizes reactive Cu–O or Mn–O species involved in the cooperative catalytic cycle. Therefore, this study highlights how water can promote bimetallic C–N coupling beyond its conventional role as a benign reaction medium (Scheme 13).^85^

**Scheme 13:**
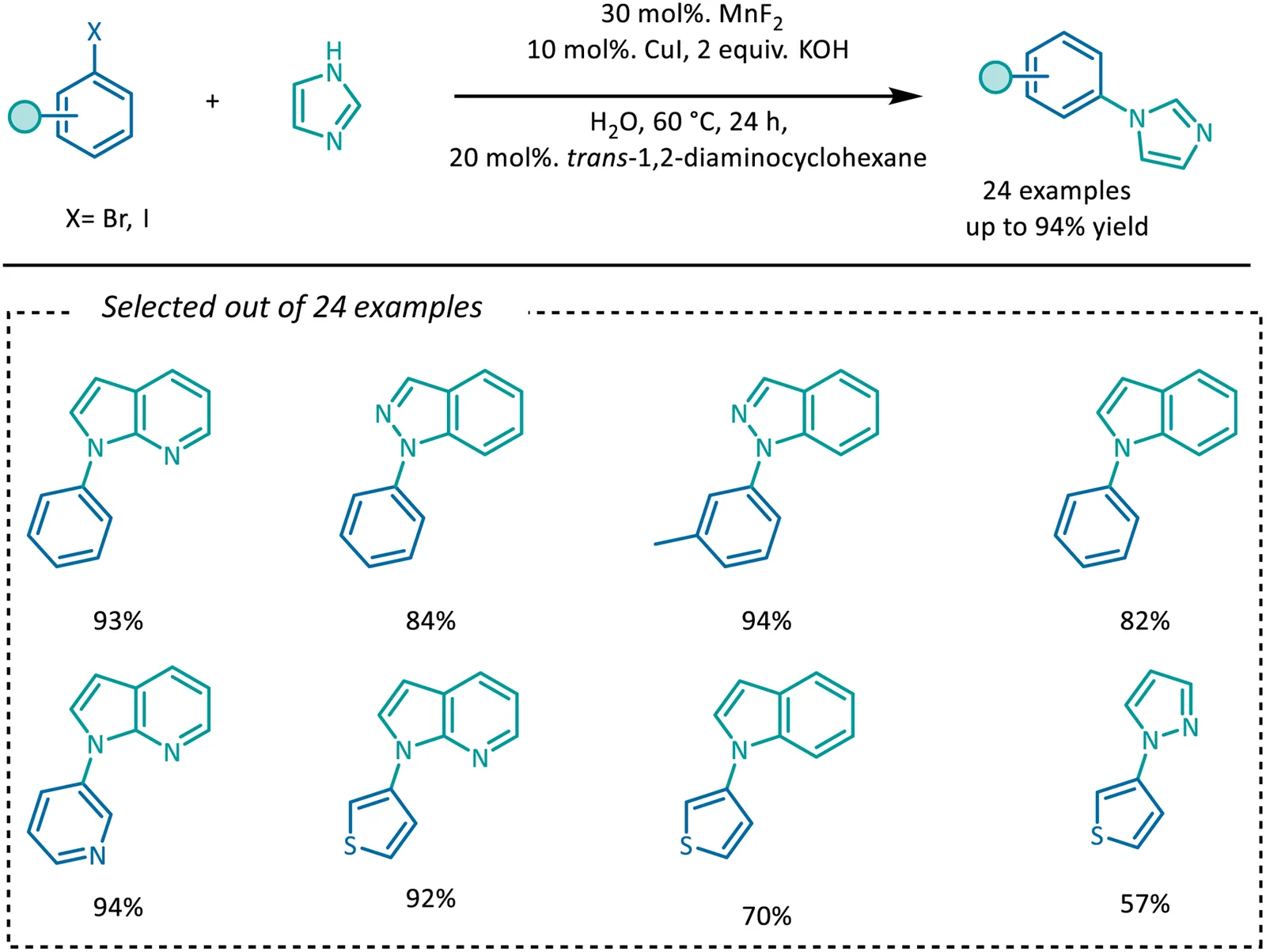
Mn-/Cu-catalysed C–N bond formation in aqueous media.

Ligand and microenvironment design have further expanded the efficiency of Cu-catalysed C–N coupling in water. Su and co-workers developed a calix[4]arene-supported amino-acid ionic liquid as a bifunctional ligand/catalyst for the Cu(i)-catalysed Ullmann-type *N*-arylation of imidazoles with aryl and heteroaryl halides under microwave irradiation (Scheme 14).^86^ In this system, water serves as the exclusive solvent, while the amphiphilic calix[4]arene framework creates a micellar-like environment that concentrates hydrophobic substrates and the copper catalyst. Notably, the combination of ligand coordination, hydrophobic substrate organization, and rapid microwave heating enables efficient coupling, including the reactions of electron-deficient aryl chlorides.

**Scheme 14:**
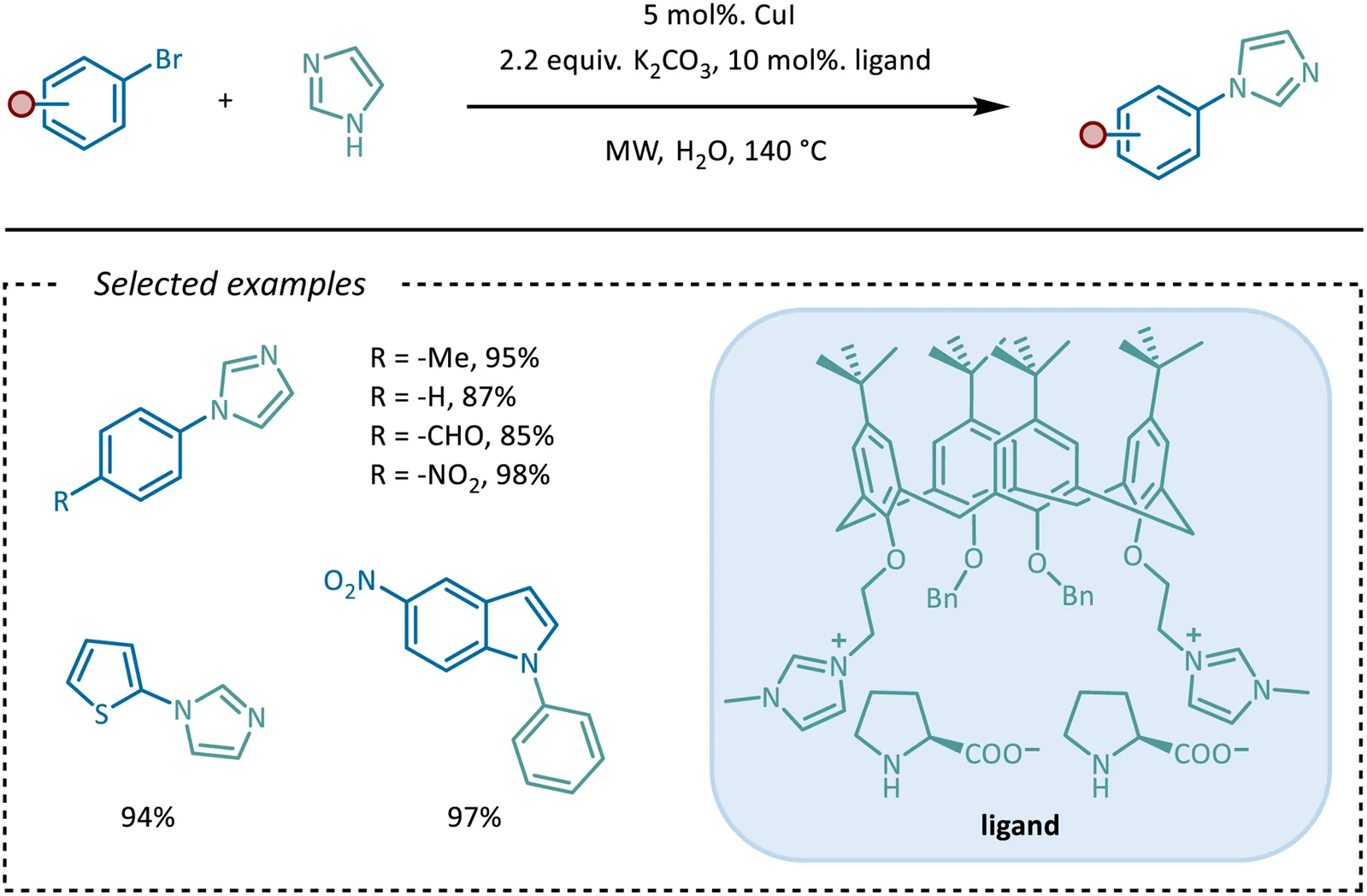
Cu(i) iodide- and calix[4]arene-catalysed C–N bond formation.

Micellar catalysis has further improved aqueous Cu-catalysed C–N bond formation. Schmitt and co-workers used d-glucose to generate active Cu(i) species *in situ* from Cu(ii) triflate, enabling the *N*-arylation of challenging primary aliphatic amines with aryl bromides under TPGS-750-M micellar conditions.^87^ The micelles concentrate hydrophobic substrates and the copper catalyst, allowing the reaction to proceed under mild conditions, from room temperature to 50 °C. Recently, in 2025, Hazra and co-workers designed a hydrophobic bifunctional ligand derived from 6-hydroxypicolinamide for Cu(i)-catalysed C–N cross-coupling in aqueous micellar media (Scheme 15).^88^ Here, the micellar core concentrates the copper–ligand complex and organic substrates, while the ligand promotes oxidative addition and reductive elimination within the confined hydrophobic environment. The system shows broad substrate compatibility and recyclability, illustrating the growing importance of ligand–micelle synergy in aqueous Cu catalysis.

**Scheme 15:**
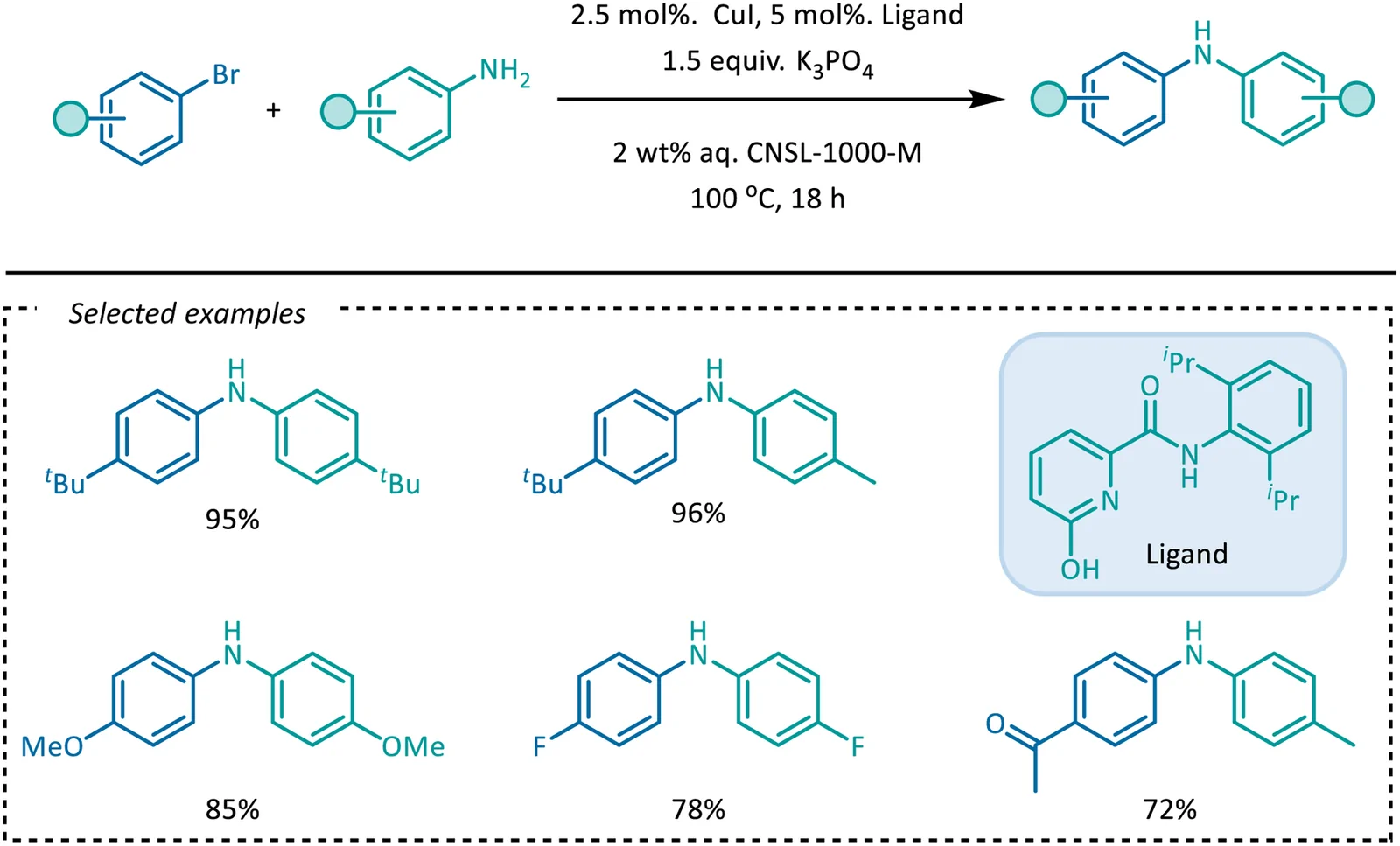
Cu(i)-catalysed C–N cross-coupling under aqueous micellar conditions.

Li and co-workers subsequently reported an efficient Cu(i)-catalysed *N*-arylation of azoles in water using 6,7-dihydroquinolin-8(5*H*)-one oxime as a ligand (Scheme 16).^89^ This system allows imidazole, benzimidazole, 1,2,4-triazole, and pyrazole to couple with aryl iodides, bromides, and even electron-deficient chlorides under relatively low CuI and ligand loadings. Mechanistically, the reaction is consistent with a ligand-supported Ullmann pathway, involving a Cu(i)–ligand complex, oxidative addition to form a transient Cu(III) species, coordination or nucleophilic attack by the azole, and reductive elimination to furnish the *N*-arylated product. The study demonstrates that suitable ligand design can overcome several limitations of classical aqueous Ullmann chemistry.

**Scheme 16:**
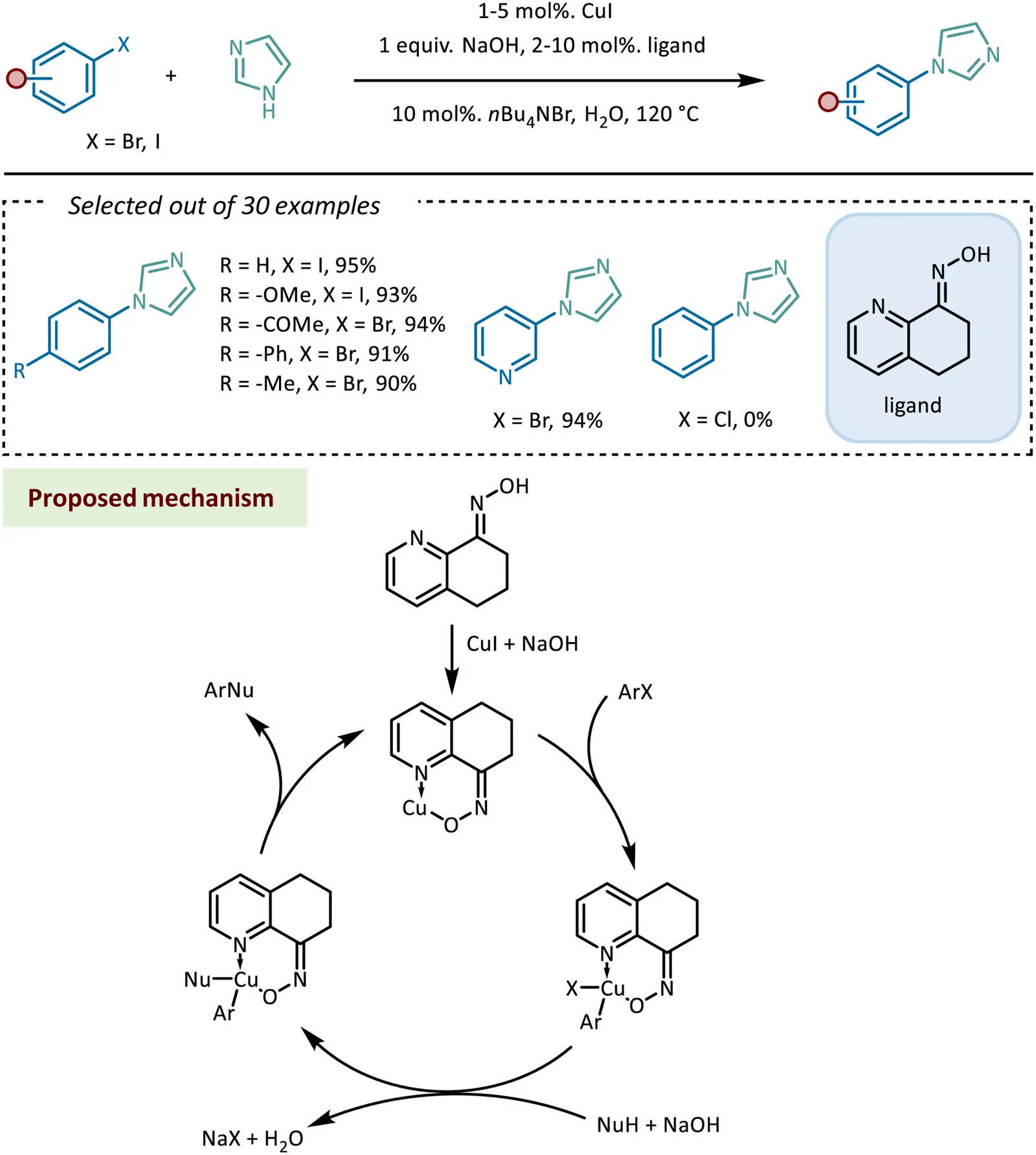
Cu-catalysed *N*-arylation of azoles in water.

Dash and co-workers unveiled an efficient Cu(i)-catalysed Ullmann-type coupling in aqueous media using readily available prolinamide ligands. Water plays a dual role; it solubilises the CuI catalyst and base while simultaneously enhancing the reactivity of poorly soluble amines through interfacial effects and stabilising key transition states (Scheme 17).^90^ This strategic advantage enabled a dramatic reduction in synthetic steps, providing direct access to complex carbazole alkaloids, such as euchrestifoline, girinimbine, and murrayacine.

**Scheme 17:**
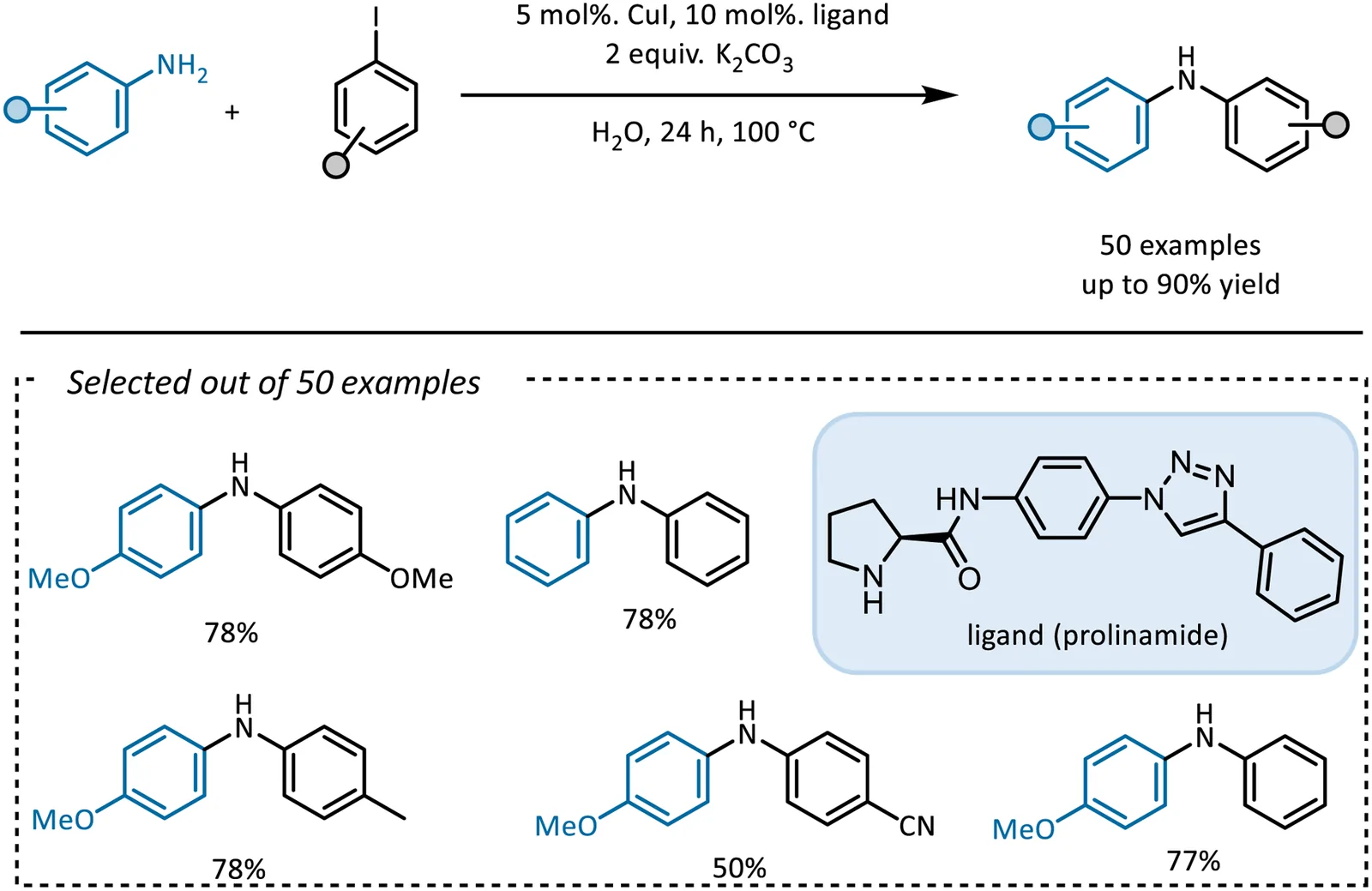
“On water” Ullmann-type C–N bond formation.

A separate but important class of aqueous Cu systems relies on oxalic acid bishydrazide ligands. Wan and co-workers developed *N*^2^,*N*^2^-disubstituted oxalic acid bishydrazides as ligands for CuO-catalysed Ullmann-type C–N coupling in water. Ligand screening revealed that free NH groups on the non-amide nitrogens were essential for activity, and microwave irradiation dramatically accelerated the reaction, reducing the reaction time from hours to minutes.^91^ Building on this strategy, Kurandina, Boyarskiy, and co-workers introduced *N*-phenyloxalyl bishydrazide combined with hexane-2,5-dione as an additive, enabling the coupling of aryl bromides with aliphatic amines using low copper and ligand loadings (Scheme 18).^92^ Importantly, this study also revealed a limitation of aqueous systems; excess water can extract water-soluble aliphatic amines from the organic reaction domain, sharply reducing yields. Boyarskiy and co-workers further developed *N*(2)-monosubstituted oxalic acid bishydrazides as efficient ligands for the Cu-catalysed *N*-arylation of aromatic amines in water. In these systems, water dissolves KOH and Bu_4_NBr, likely supports deprotonation and phase transfer, and facilitates interaction between aryl halides and amines.^93^

**Scheme 18:**
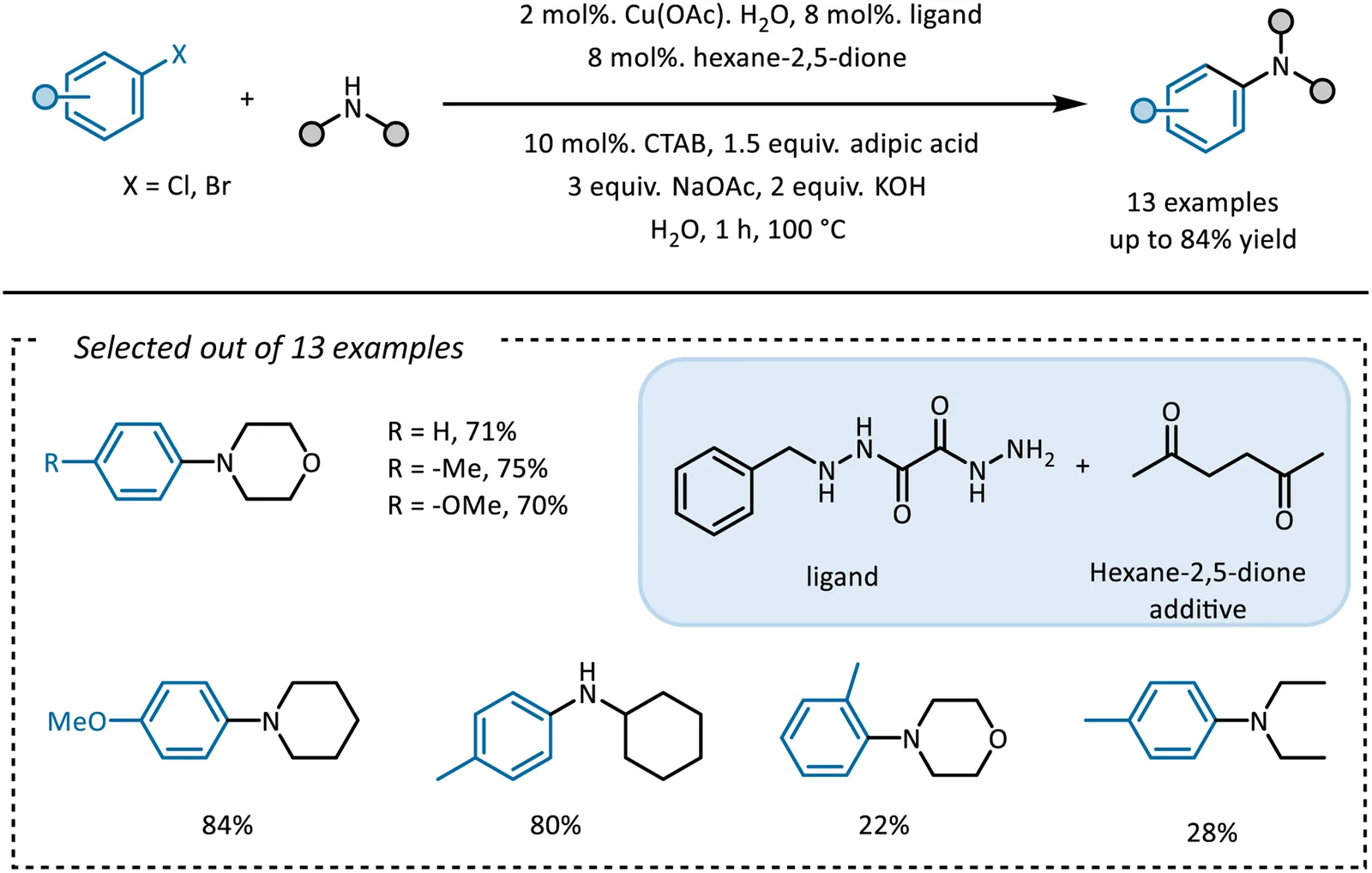
Copper-catalysed C–N bond cross-coupling of aryl halides and amines in water.

Beyond mononuclear copper systems, bimetallic cooperativity has also been exploited in water. Liu and co-workers developed a preorganized bpnp-bridged dicopper(ii) complex that enables the efficient monoamination and selective diamination of aryl dihalides in water with Cs_2_CO_3_ and TBAB (Scheme 19).^94^ The dicopper architecture outperformed mononuclear copper salts and other dicopper complexes, suggesting that proximity between the two copper centers could enhance both activity and selectivity. Water serves as the reaction medium, while TBAB improves phase transfer and substrate accessibility. This example shows how aqueous C–N coupling can benefit not only from ligand effects but also from deliberate bimetallic catalyst design.

**Scheme 19:**
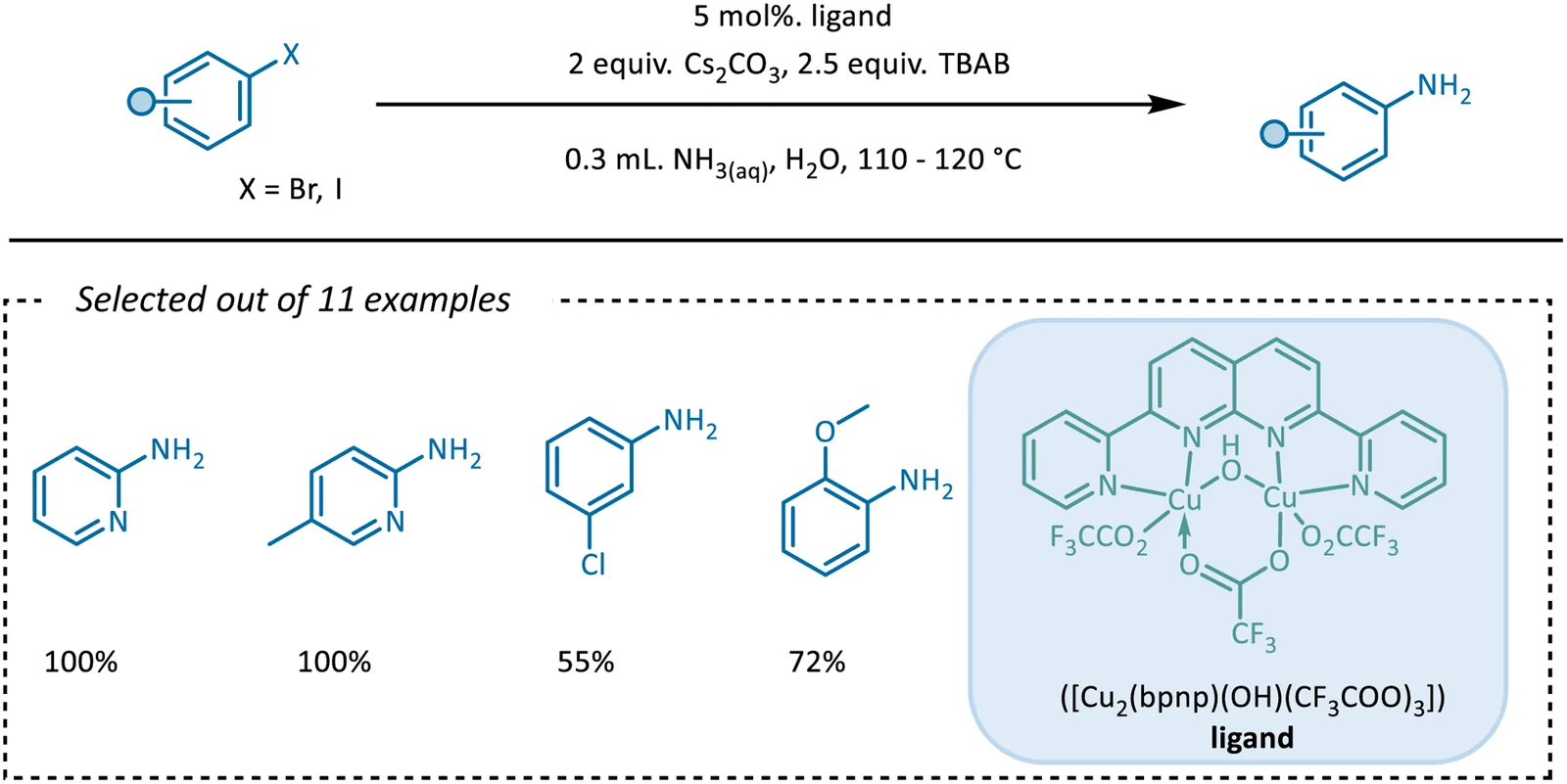
Homogeneous bimetallic catalysis for C–N bond formation.

Bardajee and co-workers reported a CuI/KF-Al_2_O_3_-promoted aqueous protocol for the synthesis of functionalized fluorescent naphthalimide dyes through C–N, C–O, and C–S Ullmann-type condensation reactions (Scheme 20).^95^ This method demonstrates the applicability of aqueous Cu catalysis to functional materials.

**Scheme 20:**
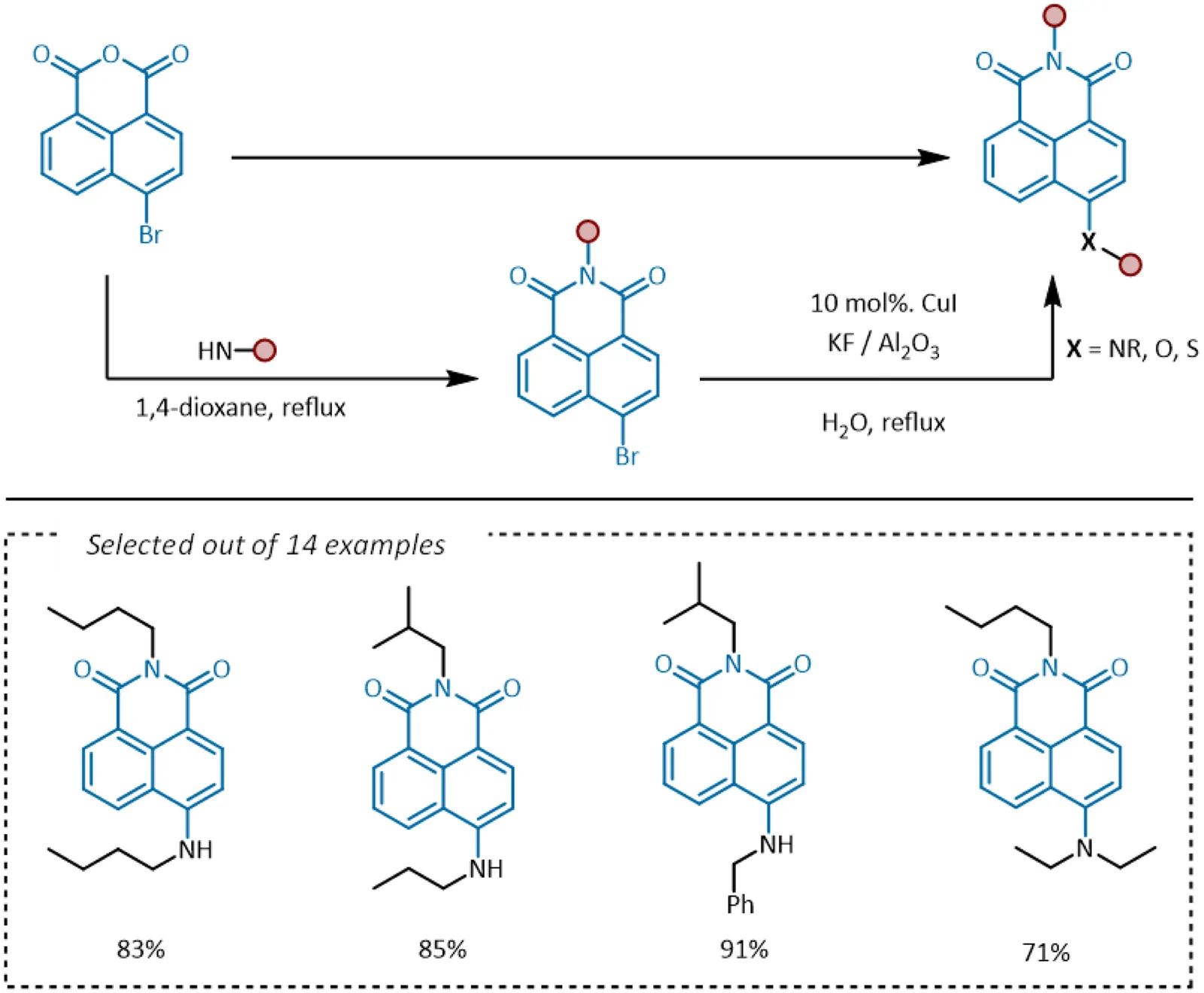
Synthesis of functionalized naphthalimide dyes *via* copper-catalysed C–N, C–O, and C–S cross-coupling.

Heterogeneous copper catalytic systems have been investigated to enhance catalyst reusability and operational simplicity. For instance, Nasrollahzadeh and colleagues introduced a nanopolystyrene-supported Cu(ii) tetrazole complex, which effectively catalysed the Chan–Lam *N*-arylation of sulfonamides with arylboronic acids in aqueous media under an air atmosphere. Water acts as a green polar medium that assists base solubility and substrate–catalyst contact, while the polymer-supported catalyst can be recovered and reused over multiple cycles. This recyclable platform provides a more practical alternative to homogeneous Cu systems, particularly where catalyst recovery and metal contamination are important considerations.^96^

#### 3.1.2 Cu-catalysed C–H functionalization

Aqueous Cu catalysis has also been extended beyond classical *N*-arylation. Adimurthy and co-workers developed a CuI-catalysed aerobic oxidative C–N/C–S bond-forming reaction for the synthesis of functionalized imidazo[1,2-*a*]pyridines through the direct C–H activation of methyl ketones and aminopyridines in water.^97^ This transformation shows that water-mediated aerobic oxidative conditions can support direct C–H functionalization, providing step-economical access to biologically relevant N-heterocycles.

#### 3.1.3 Cu-catalysed aerobic oxidative cyclization

Chen and co-workers further reported an intramolecular Cu_2_O-catalysed *N*-arylation of *ortho*-haloaryl amidines in water, affording benzimidazole derivatives in good to excellent yields with minimal decomposition (Scheme 21).^98^

**Scheme 21:**
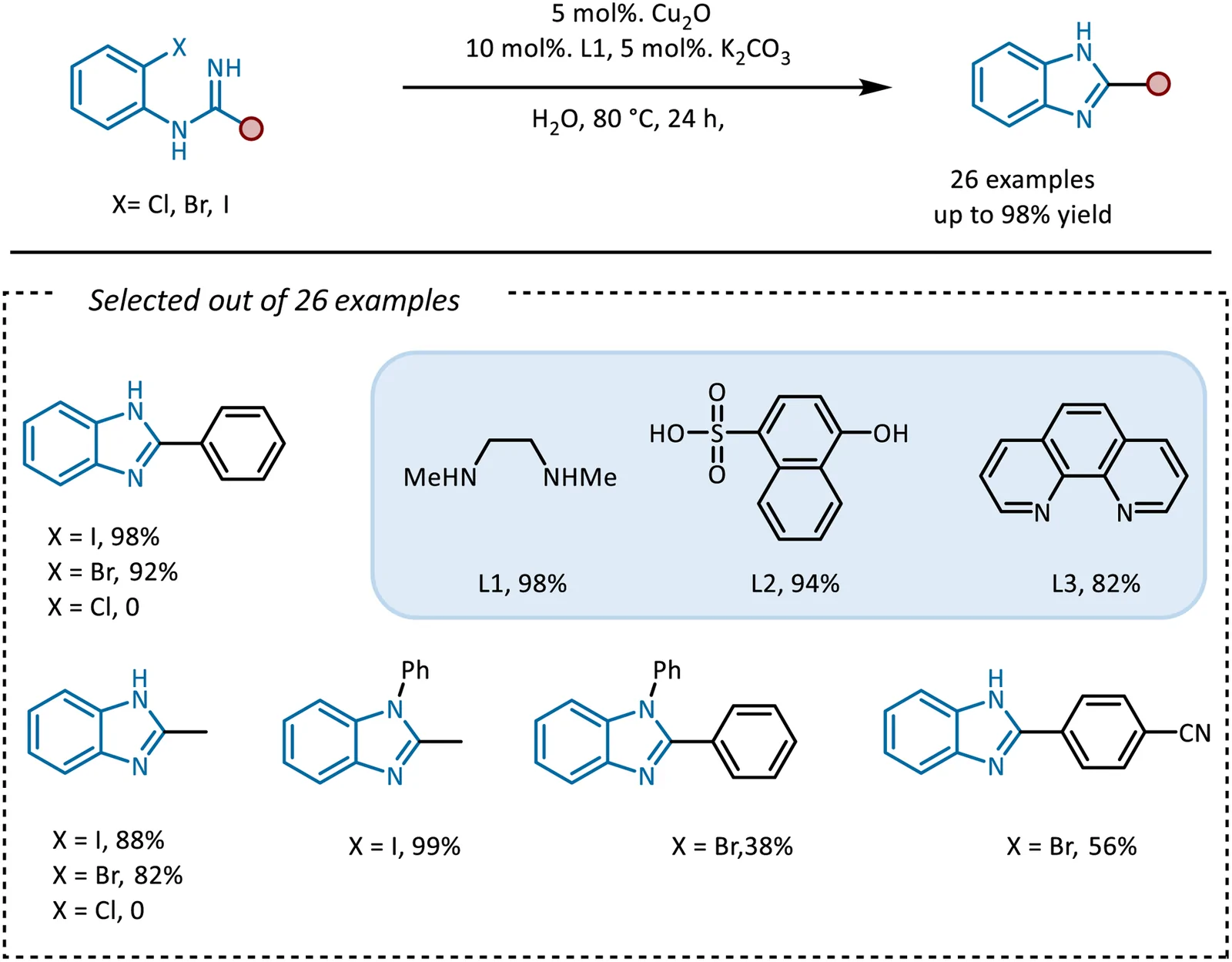
Copper-catalysed intramolecular C–N bond formation.

Aqueous Cu-catalysed C–N bond formation has evolved from simple arylation reactions into a versatile platform encompassing ligand-assisted Ullmann coupling, bimetallic catalysis, micellar systems, heterogeneous catalysts, direct C–H activation, and intramolecular *N*-arylation. In these processes, water acts not only as a green solvent but also as a medium for base/catalyst solubilization, interfacial substrate organization, micellar concentration, and stabilization of polar Cu intermediates. However, many protocols still depend on strong bases, surfactants, phase-transfer agents, microwave heating, or specially designed ligands and remain less effective for unactivated aryl chlorides, hindered amines, and complex substrates. Future efforts should prioritize mild, recyclable, low-loading copper systems with broad functional-group tolerance and clear mechanistic understanding of water-controlled copper speciation and selectivity.

### 3.2 Pd-catalysed C–N bond formation in water

#### 3.2.1 Pd-catalysed Buchwald–Hartwig aminations

Palladium catalysis has long occupied a central position in C–N bond formation, particularly through Buchwald–Hartwig amination and related C–H functionalization processes.^99,100^ Although classical Pd-catalysed C–N coupling generally relies on dry organic solvents, air-sensitive ligands, and carefully controlled conditions, recent advances show that appropriately designed catalysts, ligands, surfactants, and heterogeneous supports can enable efficient Pd-catalysed C–N bond formation in water or aqueous media. In these systems, water can act as a green solvent, micelle-forming medium, interfacial promoter, polymer-swelling agent, recyclable reaction phase, or proton-transfer mediator. This section highlights representative Pd-catalysed aqueous C–N bond-forming reactions with emphasis on how water influences reactivity, selectivity, recyclability, and practical applicability.

A major challenge in aqueous Buchwald–Hartwig amination is the poor compatibility of many aryl halides, amines, and Pd complexes with pure water. Stradiotto and co-workers addressed this limitation using the commercially available [Pd(cinnamyl)Cl]_2_/MorDalPhos catalyst system for the *N*-arylation of primary and secondary amines with a broad range of (hetero)aryl chlorides under strictly aqueous conditions, without organic co-solvents or surfactants. This example is important because it demonstrates that carefully matched Pd precatalysts and ligands can overcome the solubility and catalyst-stability limitations that usually restrict Buchwald–Hartwig chemistry in water. Moreover, the reaction enables the selective monoarylation of primary amines, showing that water can support useful chemoselectivity in Pd-catalysed C–N coupling (Scheme 22).^101^

**Scheme 22:**
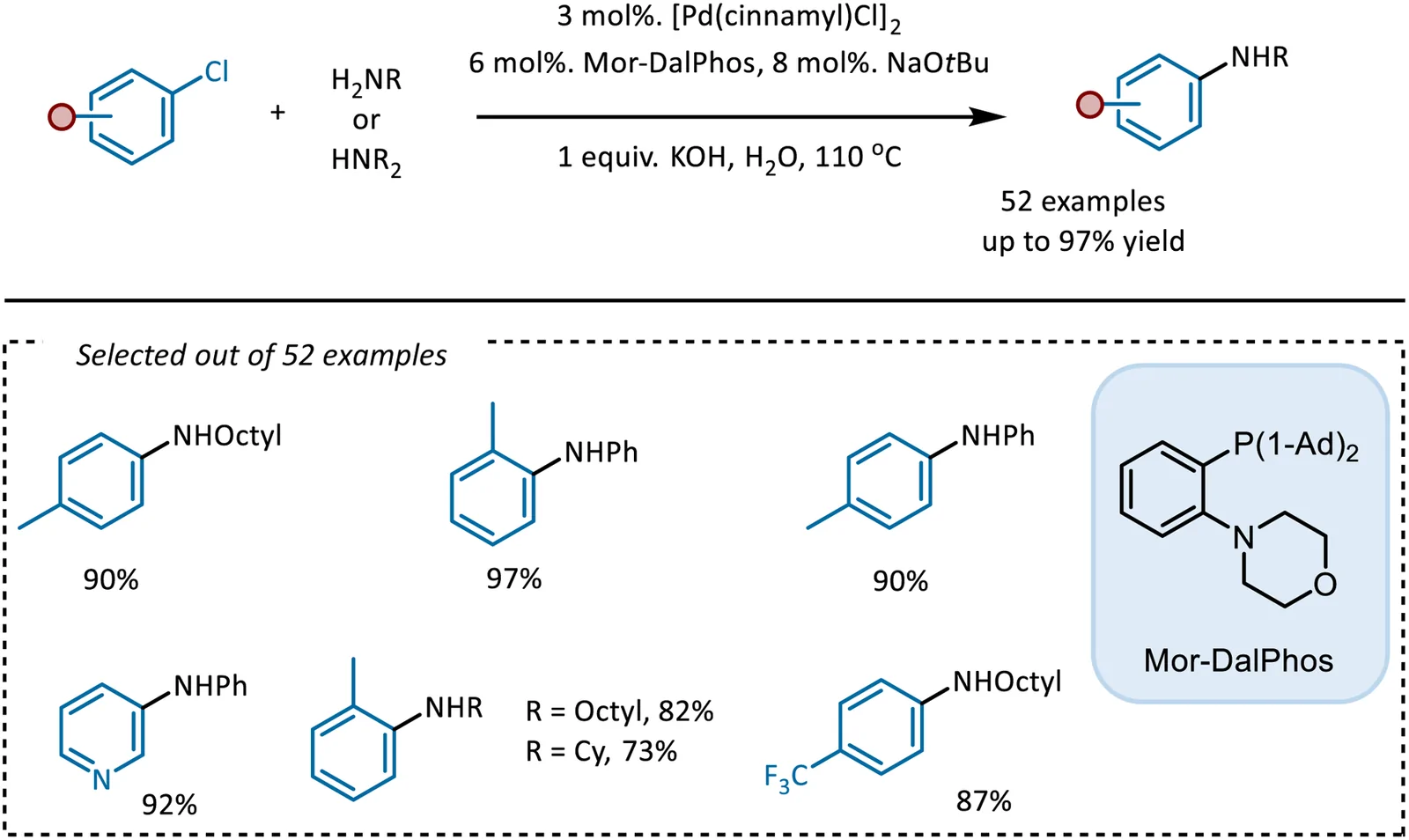
Buchwald–Hartwig amination of (hetero)aryl chlorides using Mor-DalPhos.

Micellar catalysis has provided an alternative strategy to overcome substrate insolubility in water. Schmitt and co-workers reported a Buchwald–Hartwig amination protocol using [(cinnamyl)PdCl]_2_/*t*-BuXPhos in aqueous TPGS-750-M (Scheme 23).^102^ In this system, water is believed to enable the self-assembly of the non-ionic surfactant into nanomicelles, whose hydrophobic interiors concentrate the Pd catalyst, aryl halides, and amines. The same group further expanded surfactant-assisted aqueous amination using [(allyl)PdCl]_2_/c-BRIDP in TPGS-750-M micelles. These studies show that water can indirectly promote C–N bond formation by creating lipophilic nanoreactors that mimic organic solvent environments while retaining the operational and environmental advantages of an aqueous medium.^103^

**Scheme 23:**
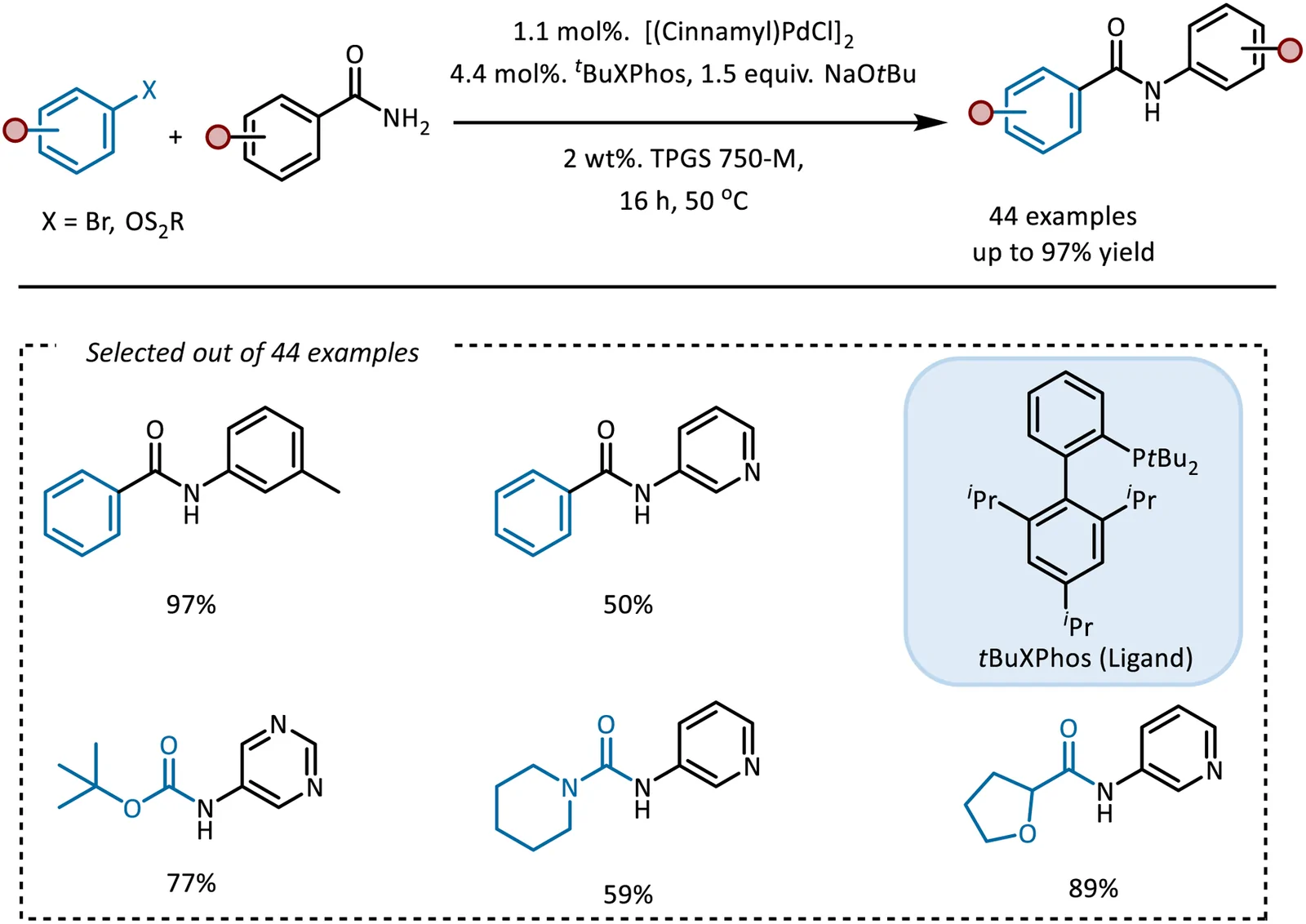
Buchwald–Hartwig coupling using the *t*-BuXPhos ligand.

More recently, Vinayagam and co-workers employed a naturally derived saponin surfactant from quillaja bark to enable the Pd-catalysed C–N coupling of aryl/heteroaryl bromides with weak nitrogen nucleophiles, including anilines, amides, carbamates, ureas, and sulfonamides (Scheme 24).^104^ The saponin-based micellar medium improves substrate organization, facilitates reactions at room temperature or mild heating, and enables the recycling of the aqueous phase. Together with TPGS-based systems, this work highlights surfactant design as a key strategy for translating Pd-catalysed C–N coupling into water.

**Scheme 24:**
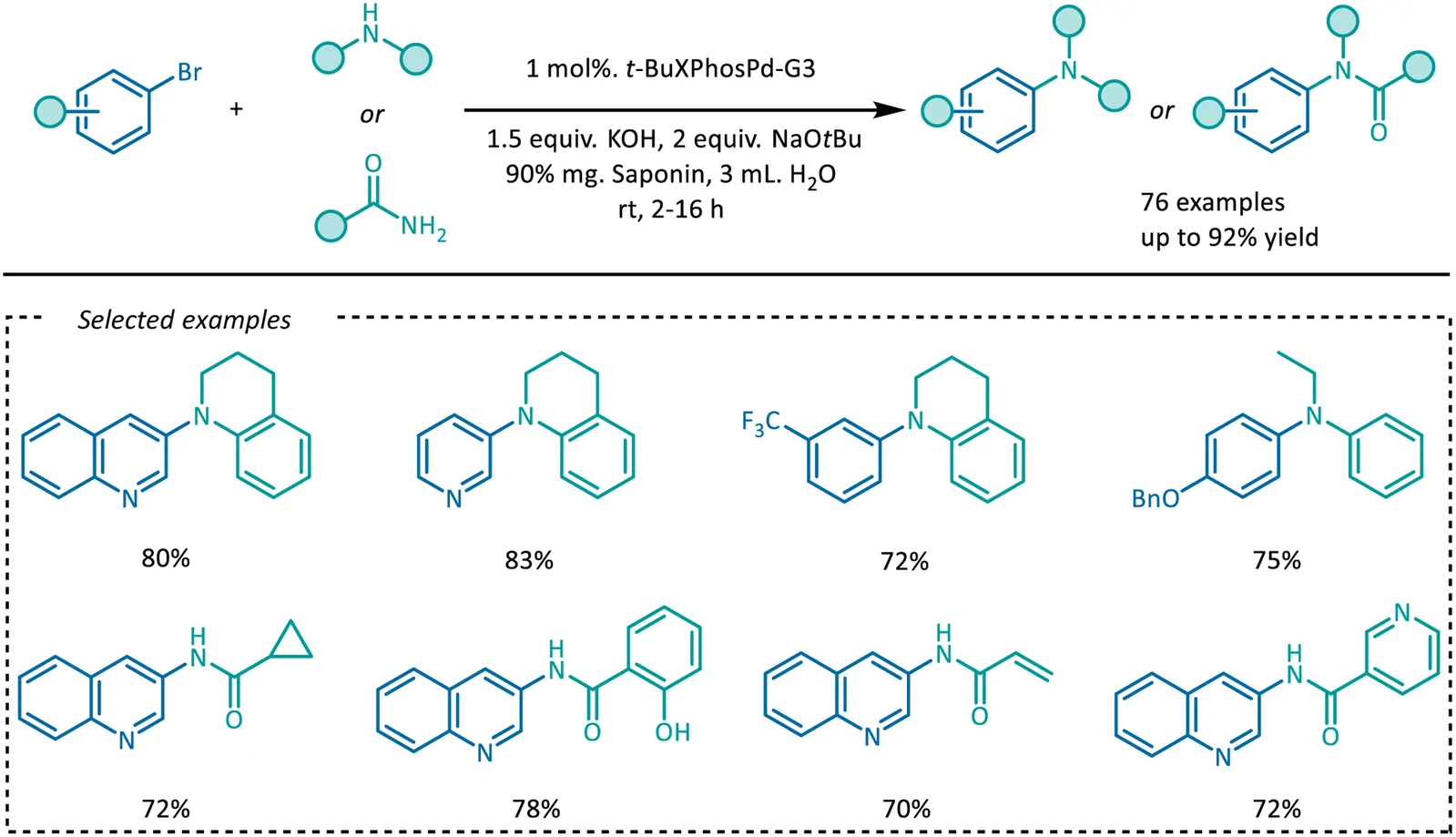
Pd-catalysed C–N bond formation with weak nitrogen nucleophiles.

Supported and heterogeneous Pd systems further improve recyclability and product purification. Uozumi and co-workers reported the asymmetric allylic amination of cycloalkenyl carbonates in water using an amphiphilic PS–PEG resin-supported chiral Pd complex.^105^ Notably, water selectively swells the amphiphilic polymer support, exposing the hydrophobic chiral Pd sites and allowing lipophilic substrates to react in a concentrated microenvironment. Unlike previous aqueous Buchwald–Hartwig systems, this protocol combines asymmetric induction with heterogeneous catalysis, achieving high yields and excellent enantioselectivity (up to 98% ee), while the heterogeneous resin support enables easy product separation and catalyst recycling. This polymer-swelling effect is essential, as the resin collapses in conventional organic solvents and loses catalytic activity. The same resin-supported Pd platform was later extended to general C–N and C–S cross-coupling reactions in water, again relying on water-induced swelling and localized substrate concentration near the Pd centres.^106^

In 2024, Lipshutz and co-workers advanced the sustainability of aqueous Pd catalysis by developing Fe nanoparticles containing only ppm levels of Pd in combination with a biodegradable surfactant, Savie, for the Buchwald–Hartwig amination of aryl bromides and pseudohalides in water (Scheme 25).^107^ This heterogeneous system operates under mild conditions, enables the recovery of the nanoparticle catalyst, and permits the recycling of the aqueous micellar medium, leading to low E-factors and very low residual Pd levels in the products.

**Scheme 25:**
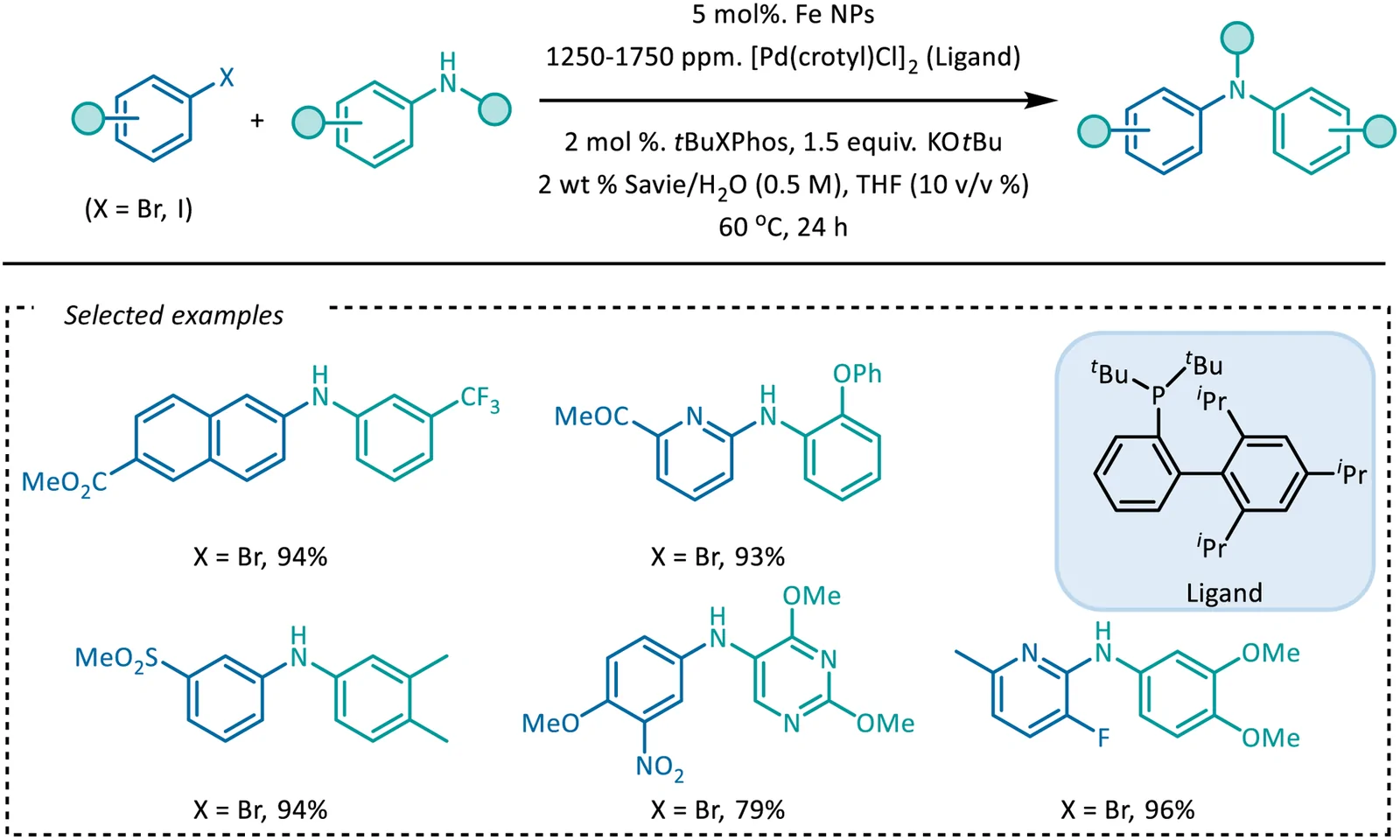
Nanoparticles as heterogeneous catalysts for ppm Pd-catalysed aminations in water.

The same group later translated aqueous Pd-catalysed amination into continuous-flow conditions using a recyclable water/*n*-PrOH medium and low Pd loadings (Scheme 26).^108^

**Scheme 26:**
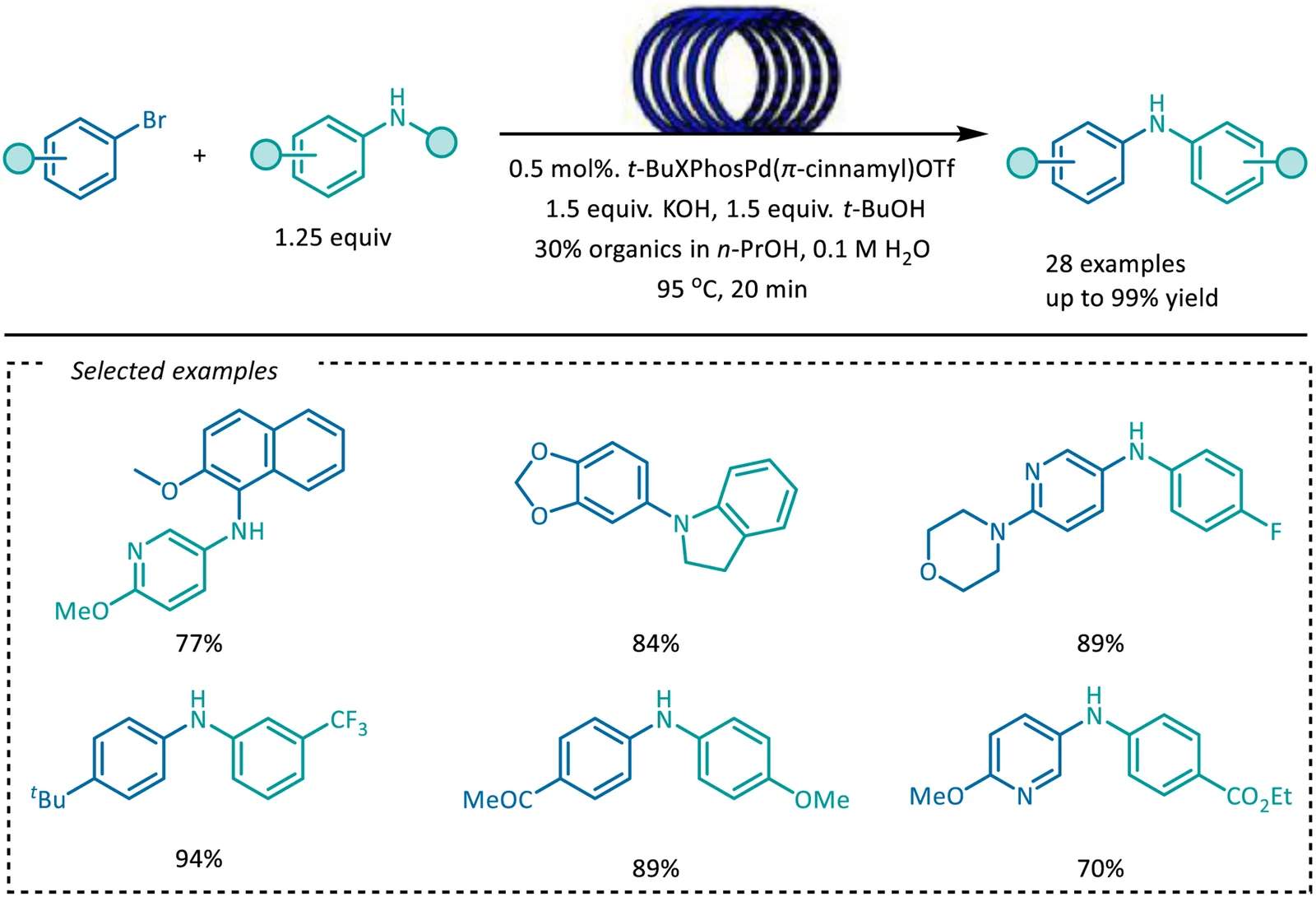
Palladium-catalysed aminations in flow on water.

This flow system shortens reaction times and improves process practicality, indicating that aqueous Pd catalysis can move beyond small-scale batch synthesis toward scalable manufacturing platforms. The authors reported that this method accommodated both aromatic and aliphatic amines and tolerated a range of functional groups, including esters, pyridines, and indoles.

#### 3.2.2 Pd-catalysed cascade cyclization

Pd-catalysed C–N bond formation in water is not limited to classical amination. Barluenga and Valdés developed an “on-water,” microwave-assisted Pd-catalysed synthesis of indoles from imines and *ortho*-difunctionalized arenes using XPhos and [Pd_2_(dba)_3_] (Scheme 27).^109^ This cascade process involves the initial α-arylation of the imine, followed by intramolecular C–N bond formation. Here, water provides an “on-water” reaction environment, while microwave irradiation rapidly heats the aqueous suspension and significantly reduces reaction times relative to conventional heating.

**Scheme 27:**
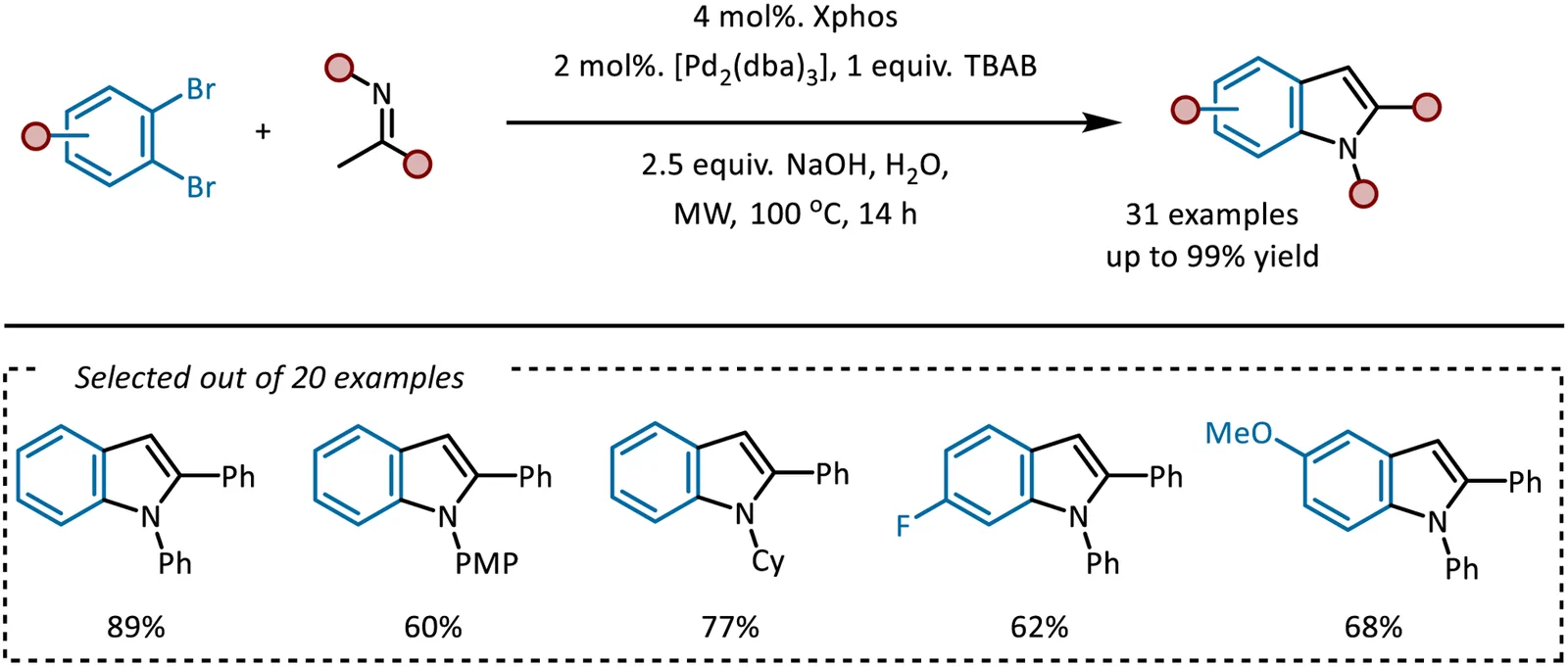
Microwave-assisted, Pd-catalysed synthesis of indoles from imines and *o*-difunctionalized arenes.

Hydrogen-transfer strategies have also been adapted to aqueous Pd-catalysed C–N bond formation.

Tang and co-workers developed a heterogeneous Pd/C-catalysed hydrogen-transfer cyclization of *ortho*-nitroacetophenones with benzylamines to access quinazolines in water (Scheme 28).^110^ The proposed pathway involves benzylamine dehydrogenation, Pd-hydride species formation, *in situ* nitro reduction, imine formation, cyclization, and final hydrogen-transfer steps. Water provides a benign medium for this multistep redox-neutral sequence, while the Pd/C catalyst enables recovery and reuse.

**Scheme 28:**
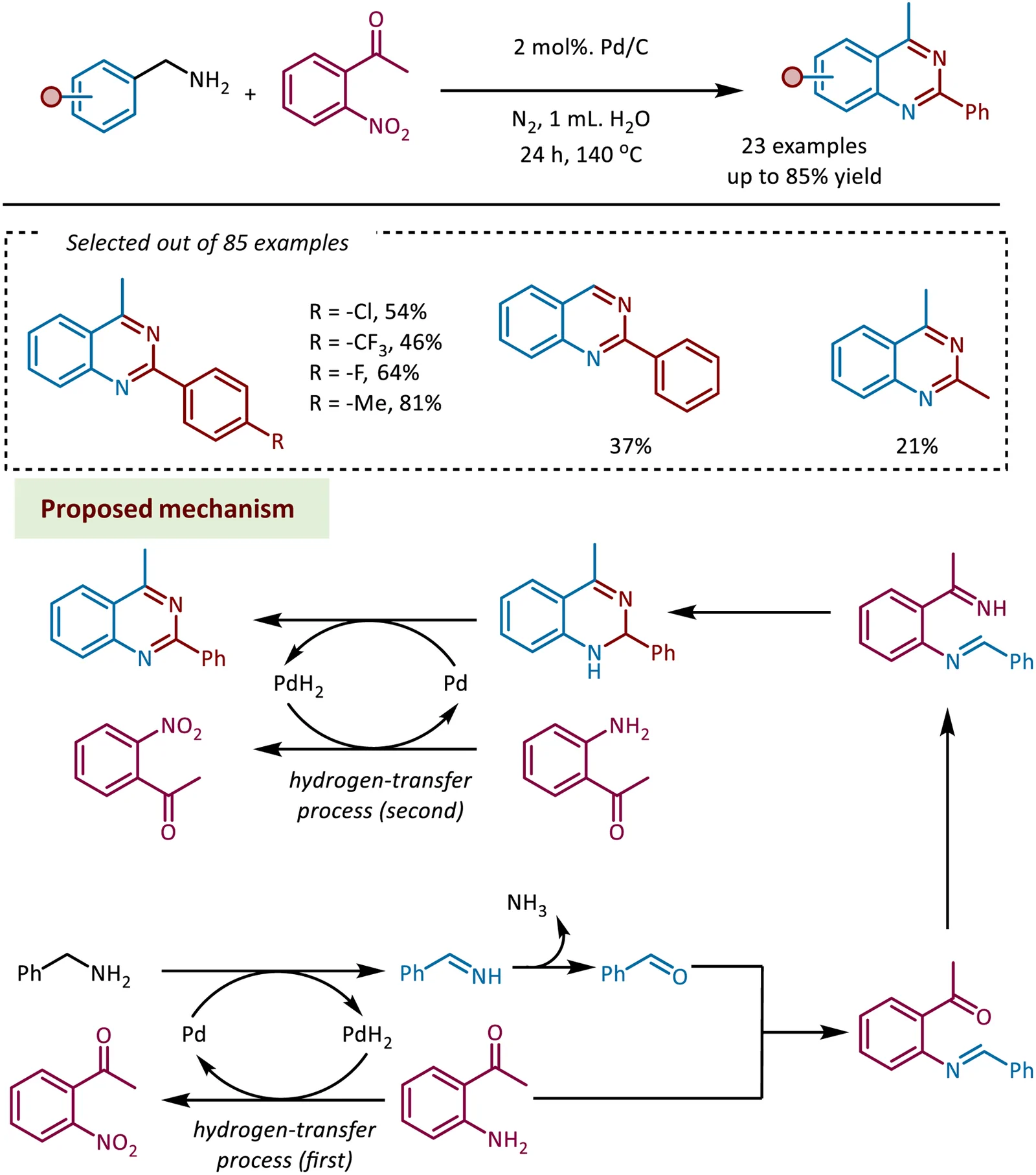
Heterogeneous Pd-catalysed hydrogen-transfer cyclization of nitroacetophenones with benzylamines.

Mechanistic studies have further clarified how water can directly influence Pd-catalysed C–N bond formation.

#### 3.2.3 Pd-catalysed C–H amination

Zhang and co-workers investigated water-assisted Pd-catalysed benzylic C–H amination using *N*-fluorobenzenesulfonimide (NFSI) as both a nitrogen source and oxidant. Combined experimental and DFT studies revealed that water likely stabilizes high-valent Pd(iv) intermediates through hydrogen-bonding networks and acts as a proton-transfer bridge during both concerted metalation–deprotonation and reductive elimination. This work is particularly important because it demonstrates that water plays a direct mechanistic role in the elementary steps of Pd-catalysed C–H amination.^111^

In conclusion, the development of C–N bond-forming reactions in water has emerged as a versatile and sustainable strategy, encompassing a variety of methodologies, including “on-water” microwave-assisted, heterogeneous Pd-catalysed, micellar, and flow-based approaches. Water serves not only as a green solvent but also as a promoter of substrate activation, enhancing solubility, reaction rates, and selectivity across a wide range of nitrogen nucleophiles. The integration of heterogeneous catalysts, surfactants, and micellar systems enables efficient C–N bond formation under mild conditions while allowing catalyst and solvent recyclability. Collectively, these strategies highlight the potential of aqueous media to provide environmentally benign, operationally simple, and broadly applicable solutions for the synthesis of biologically and pharmaceutically relevant amines, amides, ureas, carbamates, and sulfonamides.

### 3.3 Ir-catalysed C–N bond formation in water

#### 3.3.1 Ir-catalysed transfer hydrogenation and borrowing-hydrogen amination

Iridium catalysis has emerged as a powerful platform for aqueous C–N bond formation, particularly in reductive amination, borrowing-hydrogen chemistry, and tandem hydrofunctionalization processes. Unlike Cu- and Pd-catalysed systems, which are mainly associated with aryl–nitrogen cross-coupling, Ir-catalysed aqueous reactions often rely on hydrogen-transfer pathways, imine/iminium intermediates, and *in situ* generation of Ir–H species. In these transformations, water can act as a green medium, pH-controlling environment, substrate-activation platform, proton-transfer medium, or even a hydrogen source.

Xiao and co-workers developed an efficient “on-water” reductive amination of aldehydes and ketones using cyclometallated Ir complexes and formate as the hydrogen donors (Scheme 29).^112^

**Scheme 29:**
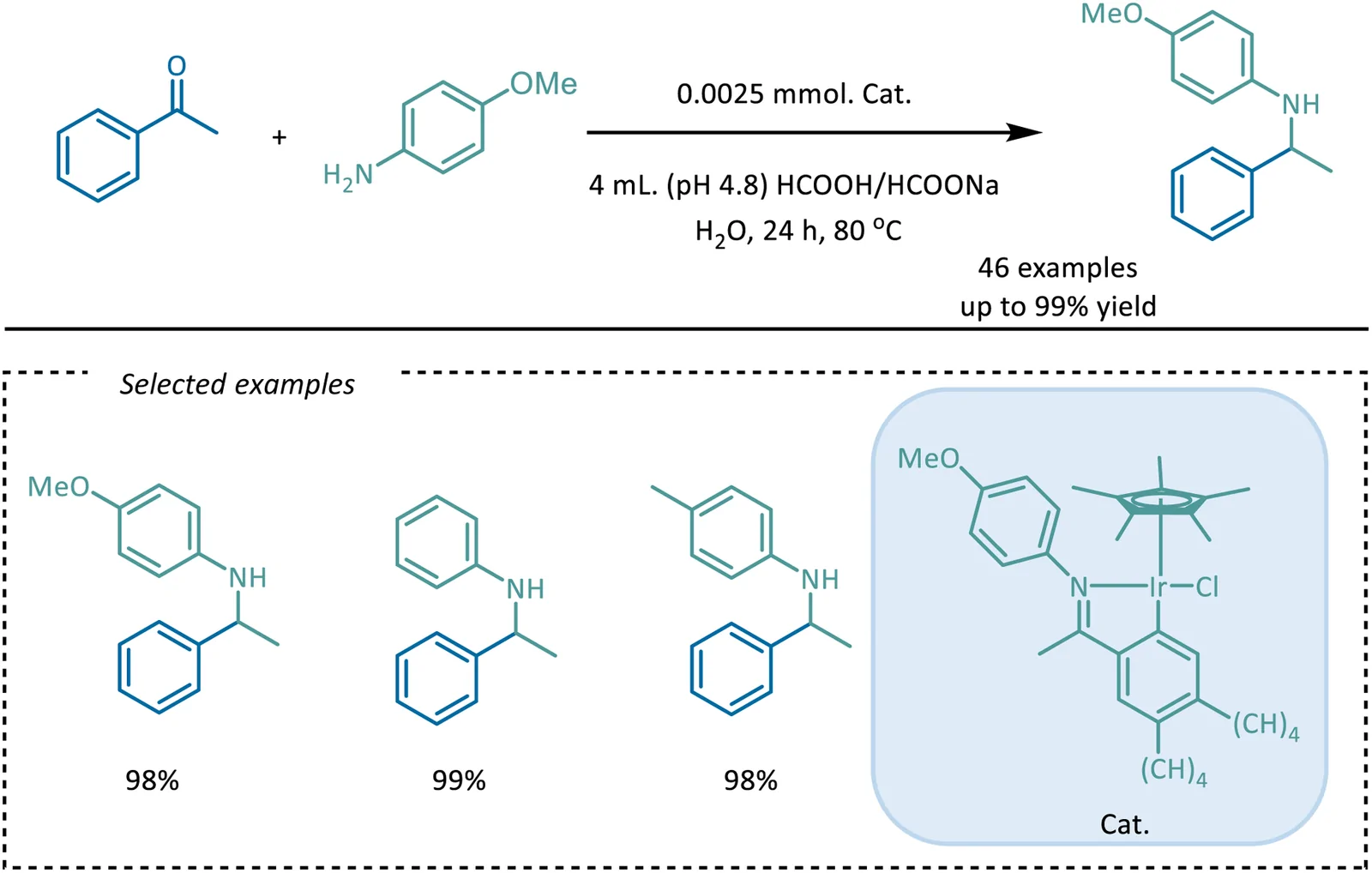
Reductive amination by transfer hydrogenation “on water”.

In this system, the aqueous medium enables precise pH control through a HCOOH/HCOONa buffer, with pH 4.8 being optimal for balancing carbonyl condensation, imine/iminium formation, and catalyst stability. This example demonstrates that water likely serves as an active medium for transfer-hydrogenative C–N bond formation. Fujita and co-workers reported an Ir-catalysed *N*-alkylation of aqueous ammonia with alcohols to give primary amines *via* a borrowing-hydrogen pathway. Water-soluble dicationic Ir–NHC complexes bearing diammine ligands allow the direct use of safe, inexpensive aqueous NH_3_, avoiding gaseous or liquefied ammonia and minimising waste.^113^ Extending this hydrogen-transfer manifold, Luo and co-workers developed a one-pot Ir-catalysed reductive hydroamination of terminal alkynes with amines under aqueous conditions using formic acid as the hydrogen donor (Scheme 30).^114^ In this reaction, water first promotes the Markovnikov hydration of the terminal alkyne to generate a ketone intermediate, which then condenses with the amine to form an imine or iminium species. Subsequent reduction by Ir–H, generated from formic acid decarboxylation, furnishes the corresponding aliphatic amines. Thus, water plays a direct substrate-activation role by converting alkynes into carbonyl-type intermediates suitable for reductive amination.

**Scheme 30:**
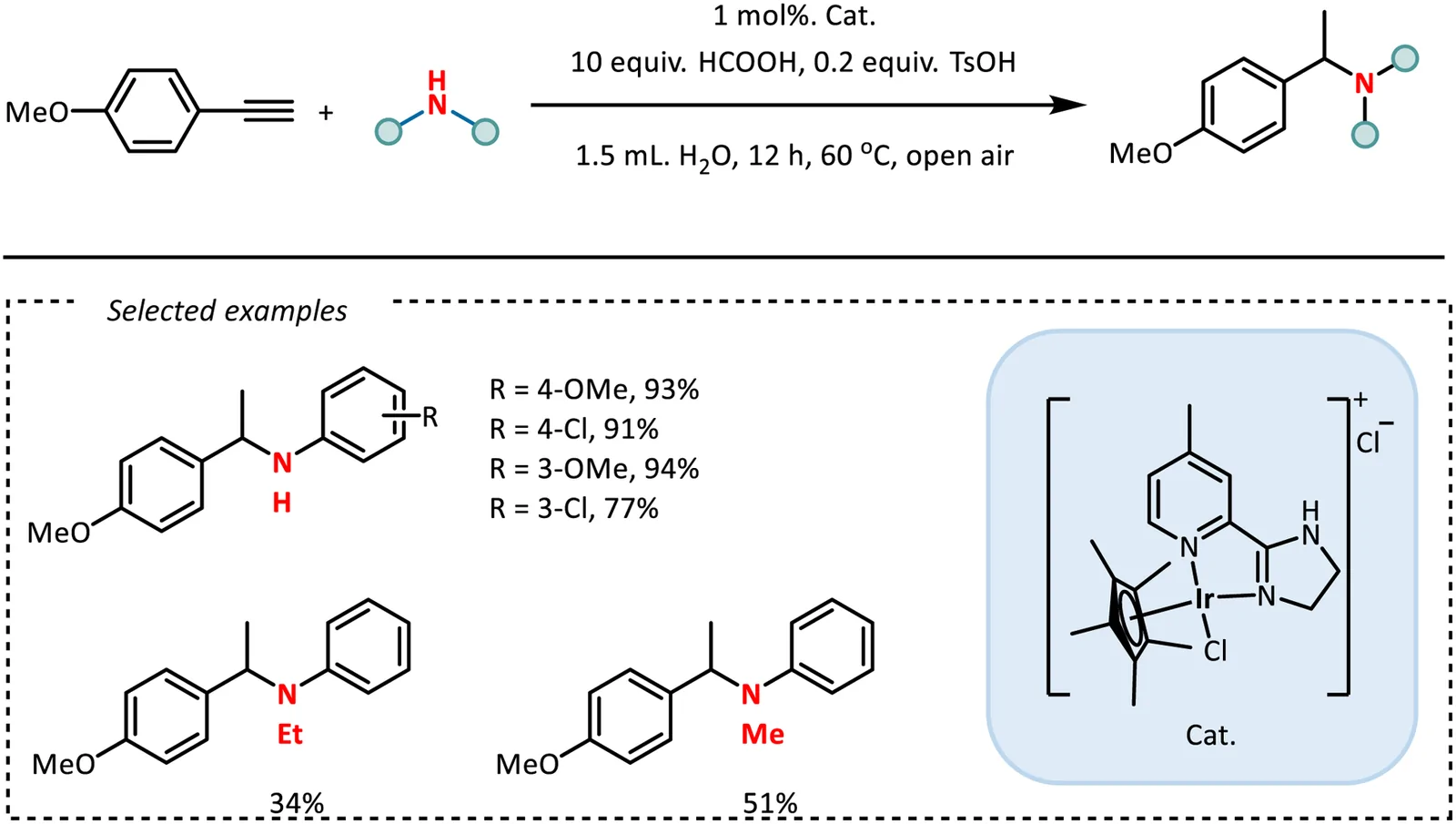
Iridium-catalysed reductive hydroamination of terminal alkynes.

More recently, Tu and co-workers developed a hydrophilic bis-NHC–Ir complex for the additive-free hydroaminomethylation of long-chain olefins in neat water (Scheme 31).^115^ This system couples long-chain alkenes with various amines, including primary and secondary anilines and heterocyclic amines, to afford linear amines in high yields and excellent regioselectivity. Mechanistically, water was proposed to participate in the Ir-catalysed water–gas shift reaction with CO to generate H_2_ and CO_2_*in situ*. The alkene then undergoes hydroformylation to give a linear aldehyde, followed by condensation with the amine and reduction by the *in situ*-generated hydrogen. Deuterium-labelling and kinetic studies confirmed that water acts not only as the solvent but also as the hydrogen source, with hydrogen generation through the water–gas shift process likely being the rate-determining step.

**Scheme 31:**
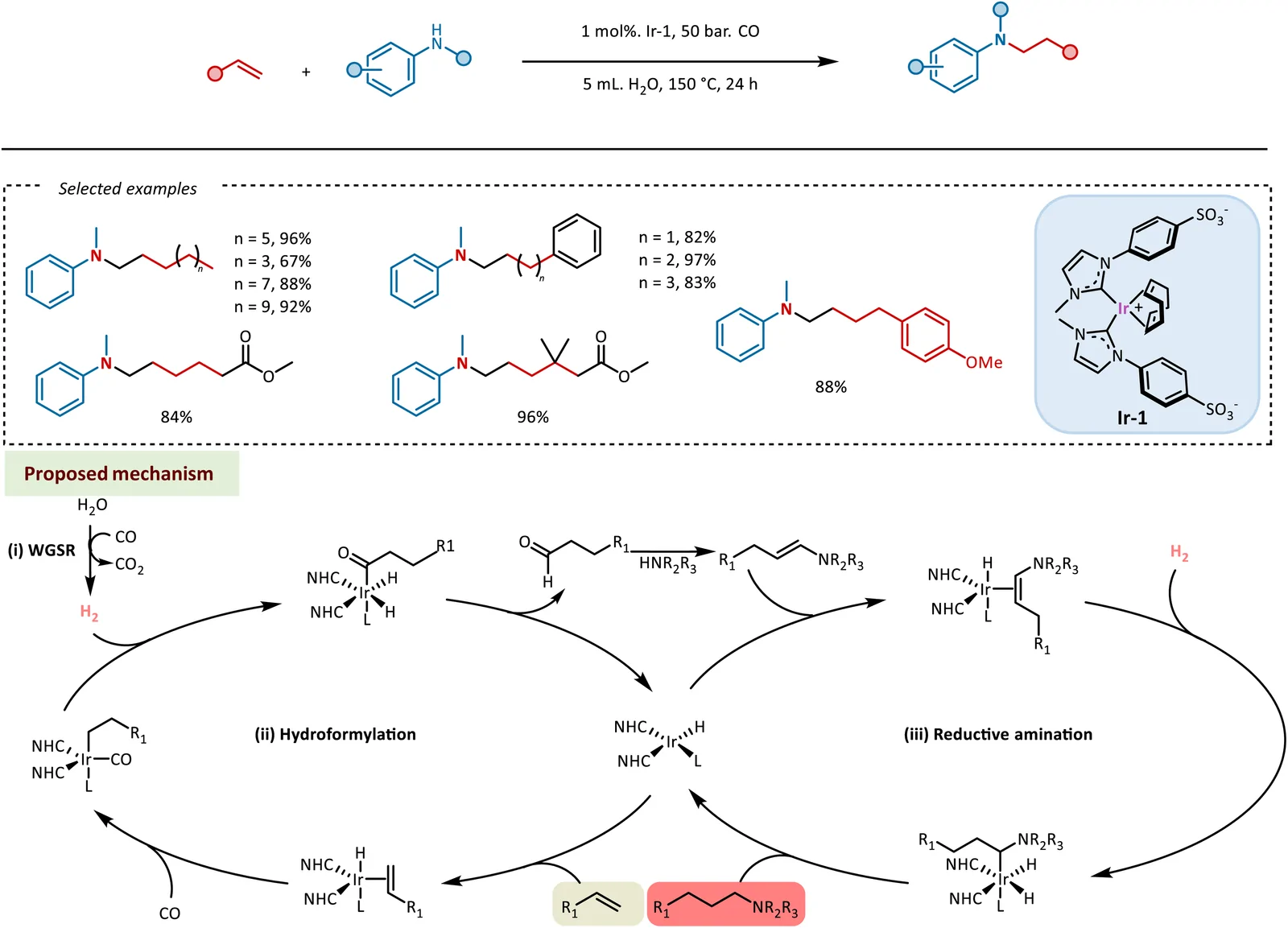
Iridium bis-N-heterocyclic carbene complexes catalyze the hydroaminomethylation of long-chain olefins in water.

Overall, Ir-catalysed C–N bond formation in water is dominated by hydrogen-transfer and tandem reductive amination pathways rather than classical cross-coupling. Water plays diverse roles as a pH-regulating medium, ammonia-compatible solvent, alkyne-hydration promoter, proton-transfer environment, and, in hydroaminomethylation, a direct hydrogen source. Despite these advances, the high cost of Ir, the need for specialized water-soluble ligands, and the limited substrate generalizability of some systems remain important challenges. Future progress should focus on recyclable Ir catalysts, low metal loading, broad compatibility with complex amines and unsaturated substrates, and deep mechanistic understanding of water-assisted Ir–H generation and transfer.

### 3.4 Rh-catalysed C–N bond formation in water

#### 3.4.1 Rh-catalysed C–H amidation and C–H amination

Rhodium catalysis has enabled several distinctive C–N bond-forming reactions in aqueous media, particularly through nitrene-transfer chemistry, directed C–H activation, carbene insertion, and annulation pathways. Compared with Cu- and Pd-catalysed cross-coupling, aqueous Rh catalysis often relies on highly reactive Rh–nitrenoid, Rh–carbene, or rhodacyclic intermediates. In these transformations, water can serve not only as a green reaction medium but also as an “on-water” interfacial promoter, hydrogen-bonding activator, Brønsted acid catalyst, nucleophile, and mediator of acidic byproducts.

Our group reported the first Rh-catalysed intermolecular C(sp^3^)–H amination in a purely aqueous system, enabling direct nitrene-transfer C–N bond formation from diverse benzylic, secondary, tertiary, allylic, and biologically relevant C–H substrates (Scheme 32).^116^

**Scheme 32:**
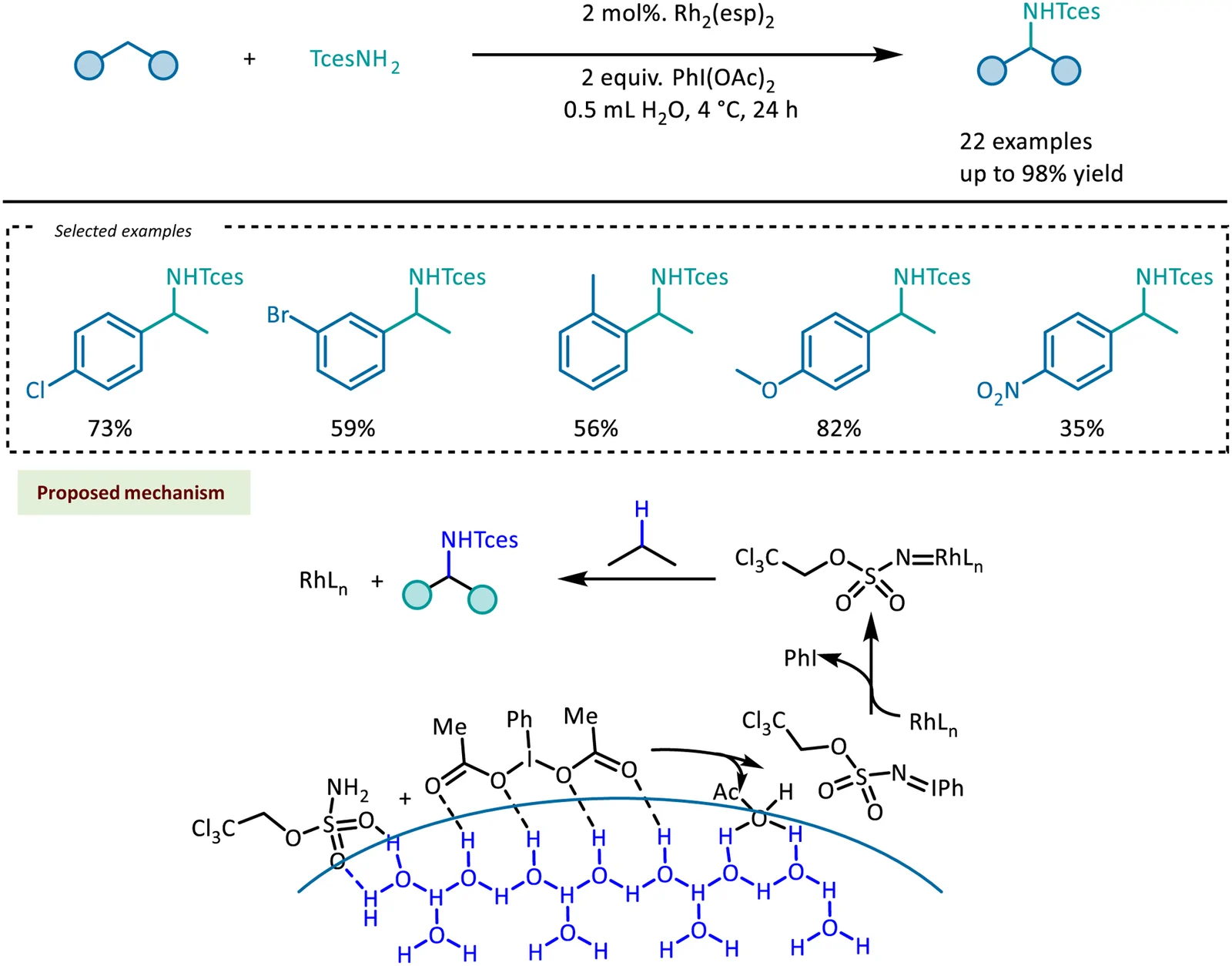
Rhodium-catalysed intermolecular C(sp^3^)–H amination in a purely aqueous system.

Using Rh_2_(esp)_2_, trichloroethylsulfamate, and PhI(OAc)_2_, the reaction proceeded efficiently under mild aqueous conditions. Mechanistically, water promotes the formation of the active iminoiodinane intermediate at the oil–water interface, likely through hydrogen bonding with the sulfamate oxygen and PhI(OAc)_2_. It also removes AcOH from the organic phase, suppresses the protonolysis of the iminoiodinane, preserves the highly electrophilic Rh–nitrenoid intermediate, and enhances intermolecular C–N bond formation through hydrophobic interfacial effects. We extended this aqueous Rh–nitrenoid strategy to the aminohydroxylation of unactivated alkenes, providing a one-pot route to valuable 1,2-amino alcohols through sequential C–N and C–O bond formation.^117^ Using Rh_2_(esp)_2_, sulfamates, and PhI(OAc)_2_, the reaction proceeds through the initial Rh-nitrenoid-mediated aziridination of the alkene, followed by the water-assisted opening of the aziridine intermediate. Notably, water plays multiple mechanistic roles; it likely promotes iminoiodinane formation at the oil–water interface, dilutes or likely removes AcOH to suppress competing acetoxylation, and directly acts as the nucleophile that opens the aziridine ring. The acidic PBS medium further functions as a Brønsted acid catalyst, activating the strained aziridine toward regioselective hydrolysis, while Rh may provide minor Lewis-acid assistance.

Rh(iii)-catalysed directed C–H amination has also been successfully adapted to aqueous systems. Lu and co-workers developed the first Rh(iii)-catalysed sp^2^ C–H *N*-Boc amidation of arenes “on water” using a stable nitrene source (Scheme 33).^118^ Mechanistic studies suggested that dangling OH groups at the hydrophobic water–organic interface activated the Rh(iii) catalyst through hydrogen bonding, thereby facilitating the formation of the rhodacyclic intermediate required for C–H activation. Water also assists in managing acidic byproducts and regenerating the arene substrate from its protonated form, allowing the reaction to proceed without an external base. The same group later extended this strategy to the selective mono- and di-amination of arenes with aryl azides, where interfacial hydrogen bonding enables a rollover pathway that provides access to a second C–H site.^119^

**Scheme 33:**
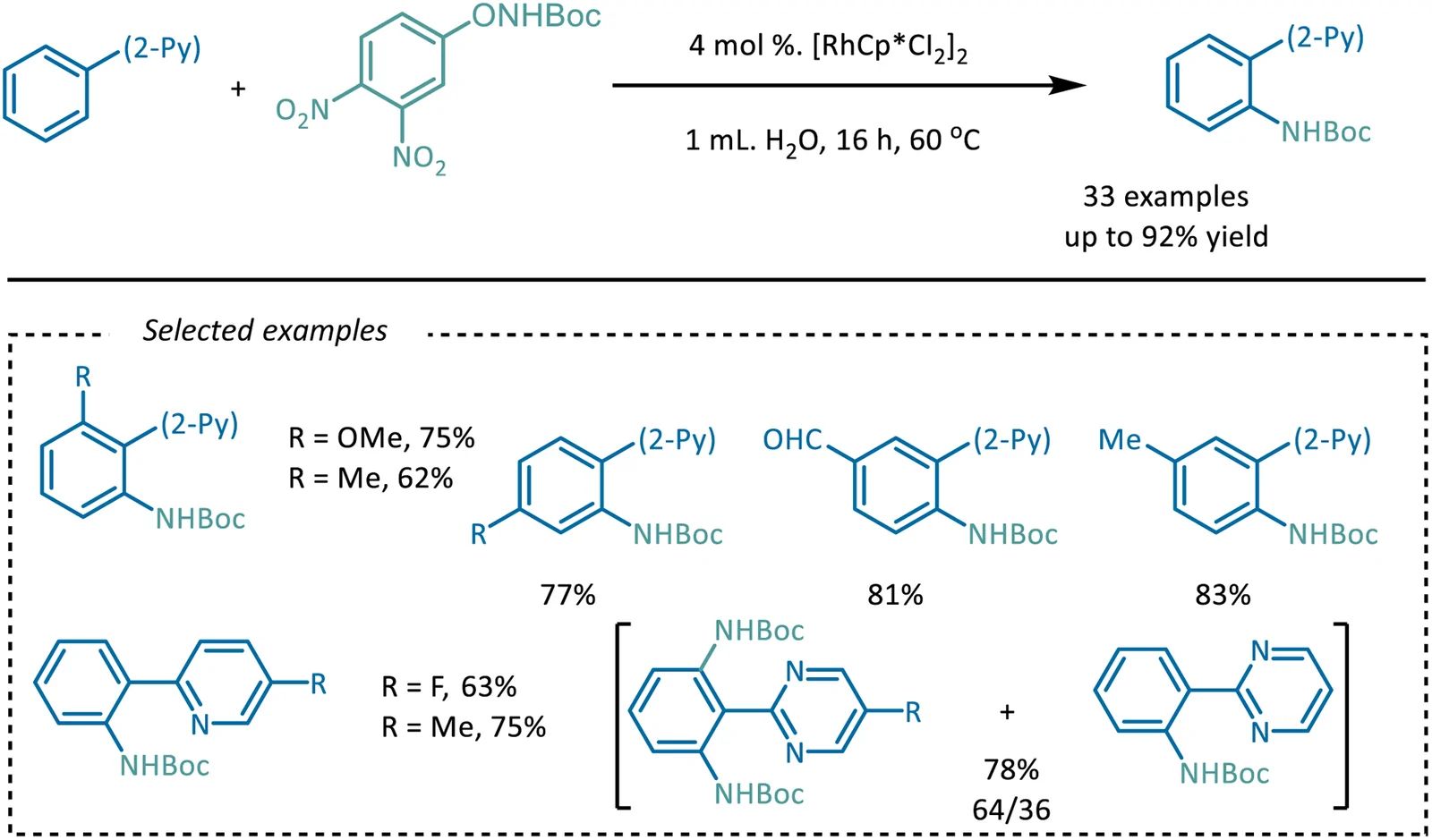
[RhCp*Cl_2_]_2_-catalysed directed *N*-Boc amidation of arenes.

Beyond nitrene-transfer amidation, Rh(iii) catalysis in water has also enabled C–N bond formation through carbene insertion and cyclization. Wang and co-workers developed a Rh(iii)-catalysed cyclization of 2-acetyl-1-arylhydrazines with diazo compounds in water, affording 1-aminoindole derivatives through a tandem C–H activation, carbene insertion, and condensation sequence.^120^ The reaction proceeds under redox-neutral conditions without external oxidants and shows broad substrate scope and excellent regioselectivity, especially at the less hindered position of meta-substituted arenes.

Ma and co-workers further developed a Rh(iii)-catalysed synthesis of isoindolin-1-ones in water through a sequential C–H activation/allene formation/cyclization pathway (Scheme 34).^121^ Readily available benzamides and propargylic acetates were converted into 3,3-disubstituted isoindolin-1-ones with excellent regioselectivity and good functional-group tolerance. Mechanistically, the reaction involves CMD-type rhodacycle formation, regioselective alkyne insertion, β-acetoxy elimination to generate an allene intermediate, and final azometalation to forge the C–N bond of the isoindolinone core.

**Scheme 34:**
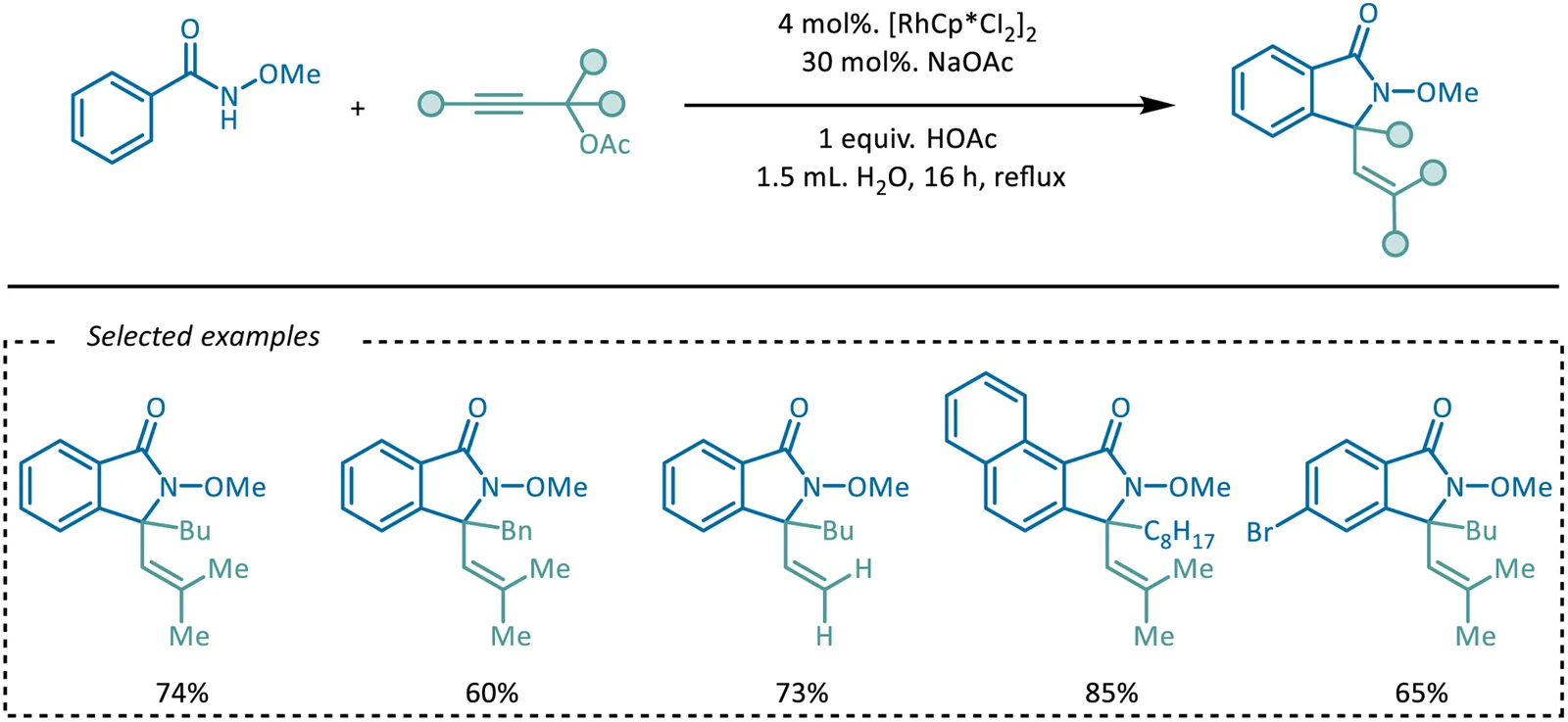
Rh(iii)-catalysed C–H functionalization in water for isoindolin-1-one synthesis.

Overall, Rh-catalysed C–N bond formation in water has progressed from direct C(sp^3^)–H amination and alkene aminohydroxylation to direct arene amidation, diamination, carbene insertion, and annulation chemistry. Across these reactions, water likely plays mechanistically diverse roles as an interfacial promoter, hydrogen-bonding activator, acid/byproduct mediator, nucleophile, Brønsted acid catalyst, and sustainable reaction medium. However, these systems still commonly require precious Rh catalysts, hypervalent iodine oxidants, directing groups, or specialized nitrene/carbene precursors. Future progress should focus on lowering Rh loading, replacing stoichiometric oxidants, improving asymmetric control, and expanding aqueous Rh catalysis to complex and pharmaceutically relevant substrates.

### 3.5 Fe-catalysed C–N bond formation in water

#### 3.5.1 Fe-catalysed C–H activation and ammoxidation

Iron catalysis offers an attractive, earth-abundant, and low-toxicity alternative to precious-metal-based C–N bond-forming systems in water. Early progress in this area was reported by Teo and co-workers, who developed the FeCl_3_/*N*,*N*′-dimethylethylenediamine-catalysed *N*-arylation of nitrogen nucleophiles with aryl halides in water. This method enabled the coupling of pyrazoles, indoles, 7-azaindoles, and benzamides with aryl halides to afford *N*-arylated products in good to excellent yields. In this system, the aqueous medium likely supports catalyst and base compatibility, stabilizes polar iron intermediates, and facilitates the redox-neutral cross-coupling process involving nitrogen coordination, aryl halide activation, and C–N bond-forming reductive elimination (Scheme 35).^122^

**Scheme 35:**
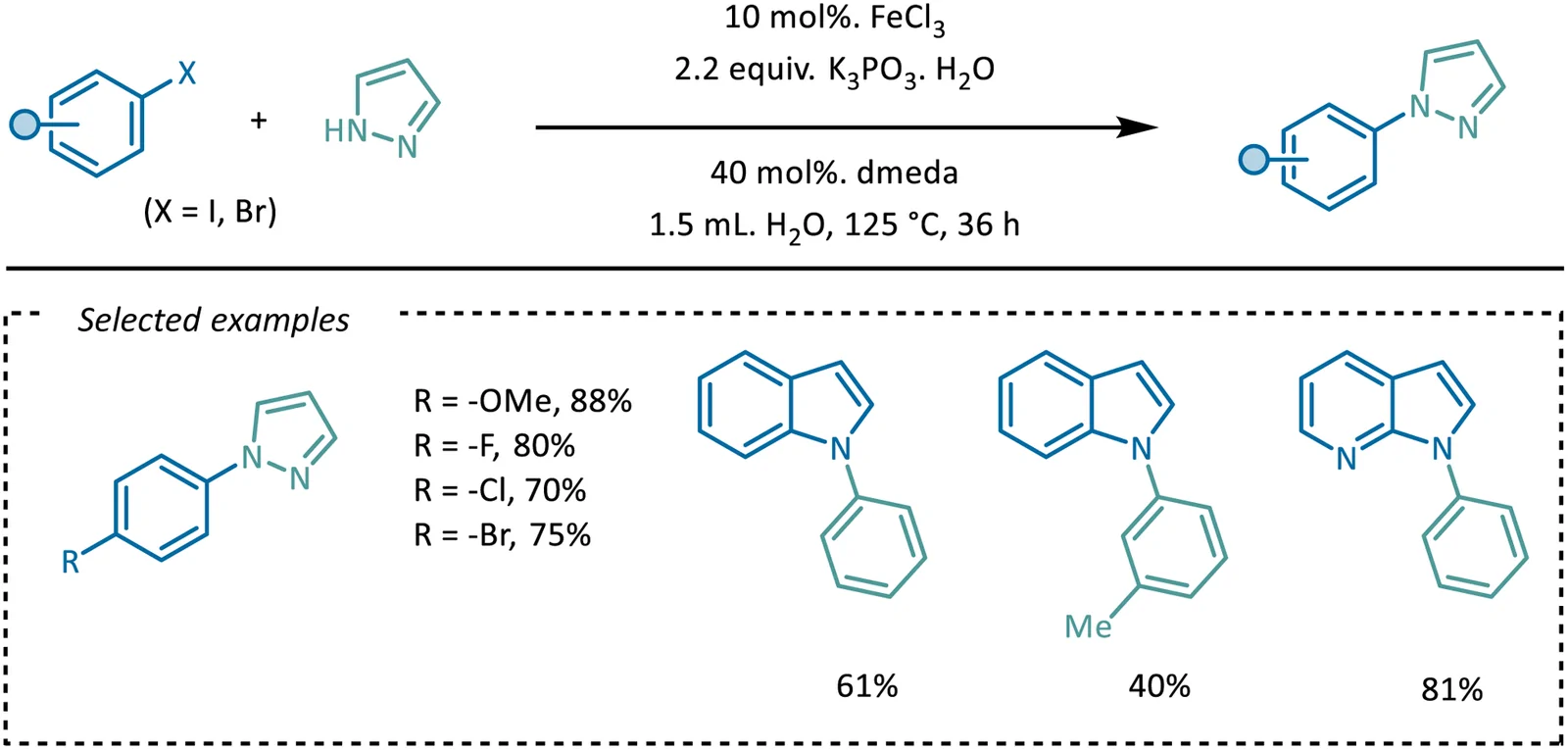
Cross-coupling reactions of nitrogen nucleophiles with aryl halides in water.

In 2022, Beller and co-workers reported a Fe_1_–N–C single-atom catalyst (SAC) for C–N bond formation in water, representing a significant advancement beyond conventional iron-based heterogeneous catalysts and precious metal systems. Derived from benzylamine-modified ZIFs and featuring isolated Fe–N_4_ active sites, this heterogeneous catalyst enables the ammoxidation of primary alcohols from benzylic and heterocyclic to allylic and aliphatic into valuable nitriles under exceptionally mild conditions (35 °C and atmospheric air) using aqueous ammonia as the nitrogen source. Notably, this work offers sustainable nitrile synthesis, offering a recyclable, earth-abundant alternative to traditional high-temperature, toxic cyanide-based methods (Scheme 36).^123^

**Scheme 36:**
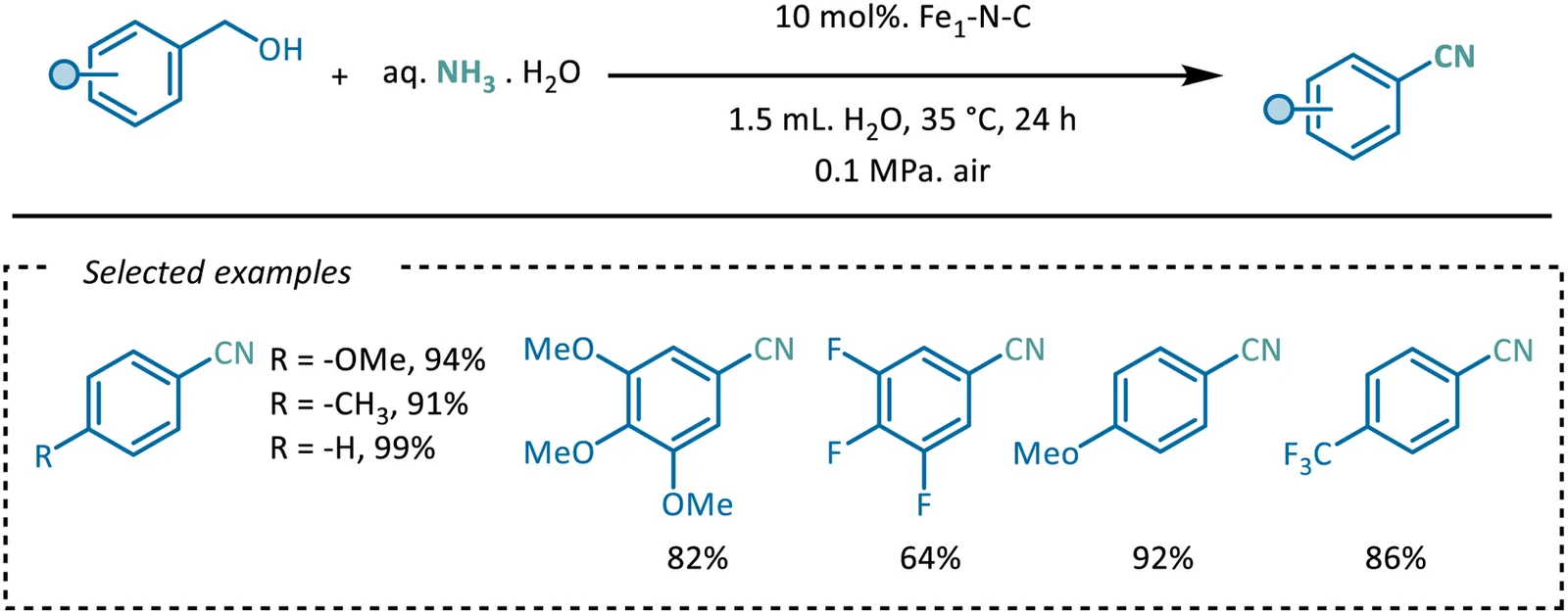
Iron single-atom catalysts for the selective ammoxidation of alcohols to nitriles.

In 2026, the same group extended this single-atom strategy to the ammoxidation of C(sp^3^)–H bonds and oxidative C–C cleavage using Fe–N_5_–B-based catalysts. The Fe@BNC-800-L1 catalyst enabled the conversion of methyl-substituted heteroarenes, alkylarenes, and lignin-derived substrates into nitriles under aqueous conditions with air and ammonia. Mechanistically, the isolated Fe–N_5_ site activates the benzylic C(sp^3^)–H bond to generate a radical intermediate, which reacts with ammonia to form an imine that is subsequently oxidized to the nitrile. In this process, water may assist substrate dispersion, stabilize the Fe–N active site, support proton-transfer steps, and enable selective nitrile formation under comparatively mild heterogeneous conditions (Scheme 37).^124^

**Scheme 37:**
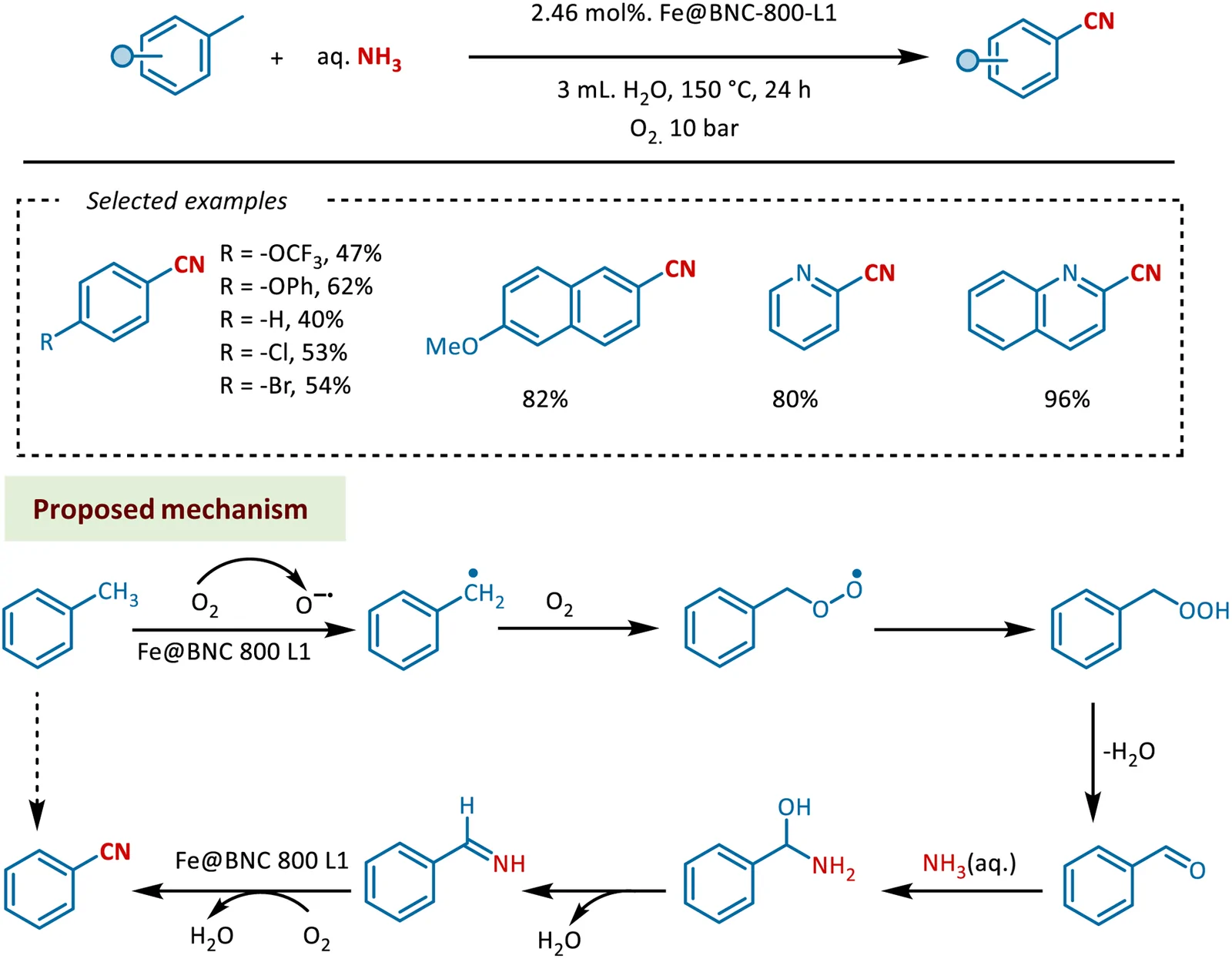
Iron-based single-atom catalysts for the selective ammoxidation of C(sp^3^)–H bonds and oxidative C–C cleavage reactions.

Fe-catalysed C–N bond formation in water has progressed from homogeneous *N*-arylation to heterogeneous single-atom-catalysed ammoxidation. These systems highlight the potential of iron as a sustainable alternative to Pd, Rh, Ir, and Au catalysts, particularly for ammonia-based nitrogen incorporation and recyclable aqueous catalysis. However, the field remains less developed than Cu- and Pd-catalysed aqueous C–N coupling. Current challenges include limited reaction diversity, the need for carefully engineered single-atom sites, the control of catalyst stability and metal dispersion, and the difficulty of functionalizing inert C–H bonds under very mild conditions. Future studies should focus on expanding Fe catalysis beyond nitrile synthesis, improving functional-group tolerance, and clarifying how water influences Fe speciation, radical formation, ammonia activation, and proton-transfer pathways.

### 3.6 Gold-catalysed C–N bond formation in water

Supported gold catalysts have also been applied to aqueous C–N bond formation, particularly in hydrogen-transfer and reductive amination-type processes. Wang and co-workers reported an ammonia-promoted synthesis of 2,4-disubstituted quinazolines from *ortho*-nitroacetophenones and alcohols in aqueous media using Au/TiO_2_ as a heterogeneous catalyst. This one-pot cascade involves alcohol dehydrogenation to aldehydes, nitro-group reduction, imine formation with ammonia, and subsequent cyclization to construct the quinazoline framework through multiple C–N bond-forming events. In this transformation, water provides a benign medium for the multistep hydrogen-transfer sequence, while the supported Au catalyst enables redox activation and catalyst recyclability (Scheme 38).^125^

**Scheme 38:**
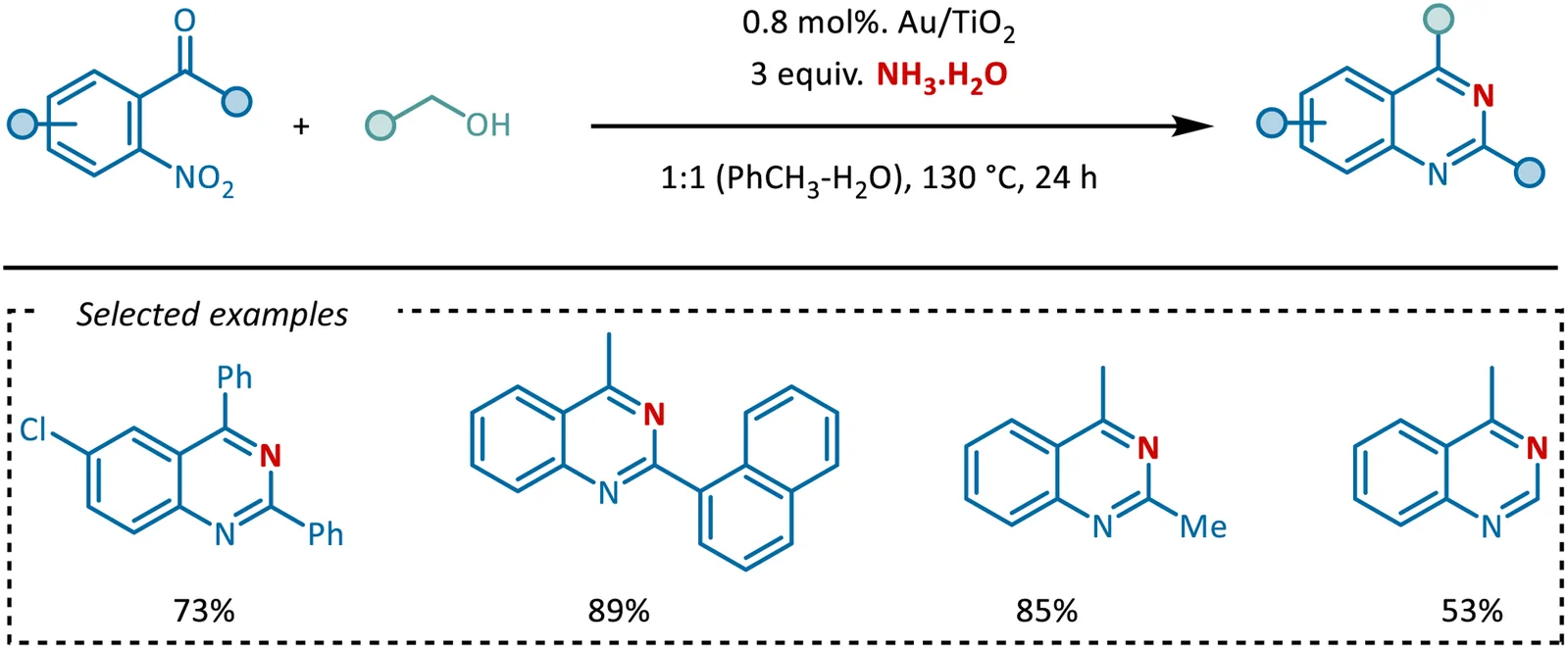
Supported gold-catalysed and ammonia-promoted selective synthesis of quinazolines in aqueous media.

A mechanistically distinct role of water was later demonstrated by Shi and co-workers in the Au/Al_2_O_3_-catalysed reductive *N*-methylation of amines with paraformaldehyde.

In this reaction, the supported nanogold catalyst promotes the condensation of amines with formaldehyde to form imine intermediates, followed by reduction to the corresponding *N*-methylated products. Isotope-labelling experiments confirmed that part of the hydrogen incorporated into the product originated from water, indicating that water functioned not only as a solvent but also as a co-hydrogen donor in the reductive amination process (Scheme 39).^126^

**Scheme 39:**
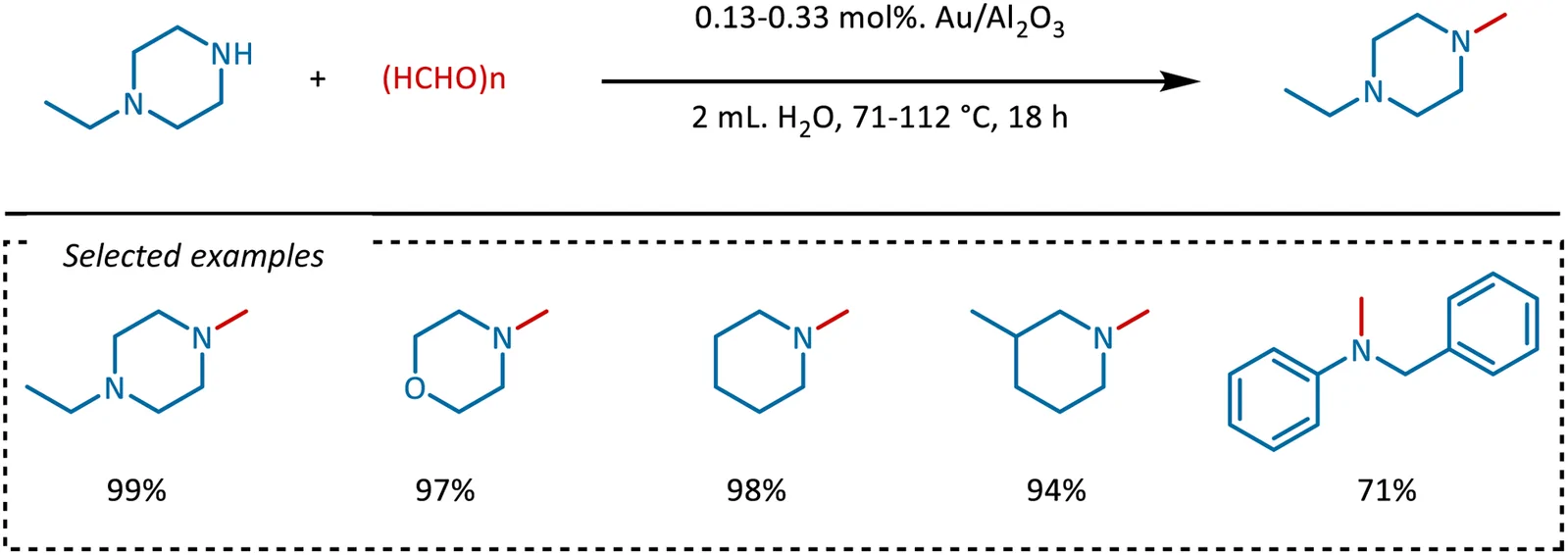
Water as a co-hydrogen donor in reductive aminations.

Overall, gold-catalysed C–N bond formation in water demonstrates the ability of supported Au nanoparticles to mediate cascade hydrogen-transfer reactions and water-assisted reductive amination under relatively mild and recyclable conditions. These examples highlight water's roles as a green medium, hydrogen-transfer participant, and, in selected cases, a direct hydrogen source. However, compared with Cu and Pd catalysis, aqueous Au-catalysed C–N bond formation remains less developed and is currently limited to specific reductive or cascade transformations. Future studies should expand substrate scope, reduce catalyst cost, and clarify how water participates in hydrogen transfer on supported Au surfaces.

### 3.7 Nickel-catalysed C–N bond formation in water

While palladium, gold, iron, and copper have dominated the landscape of aqueous C–N bond formation, nickel, a relatively cheap and abundant alternative, has received comparatively less attention. Moghaddam and co-workers addressed this gap by developing a reusable nickel ferrite (NiFe_2_O_4_) nanoparticle catalyst for the *N*-arylation of nitrogen nucleophiles with aryl halides in water under conventional heating. The heterogeneous magnetic catalyst, prepared by a simple co-precipitation method, showed high catalytic efficiency and could be readily separated from the reaction mixture using an external magnet. In this system, water serves as a benign reaction medium, while the magnetic support improves catalyst recovery and operational practicality (Scheme 40).^127^

**Scheme 40:**
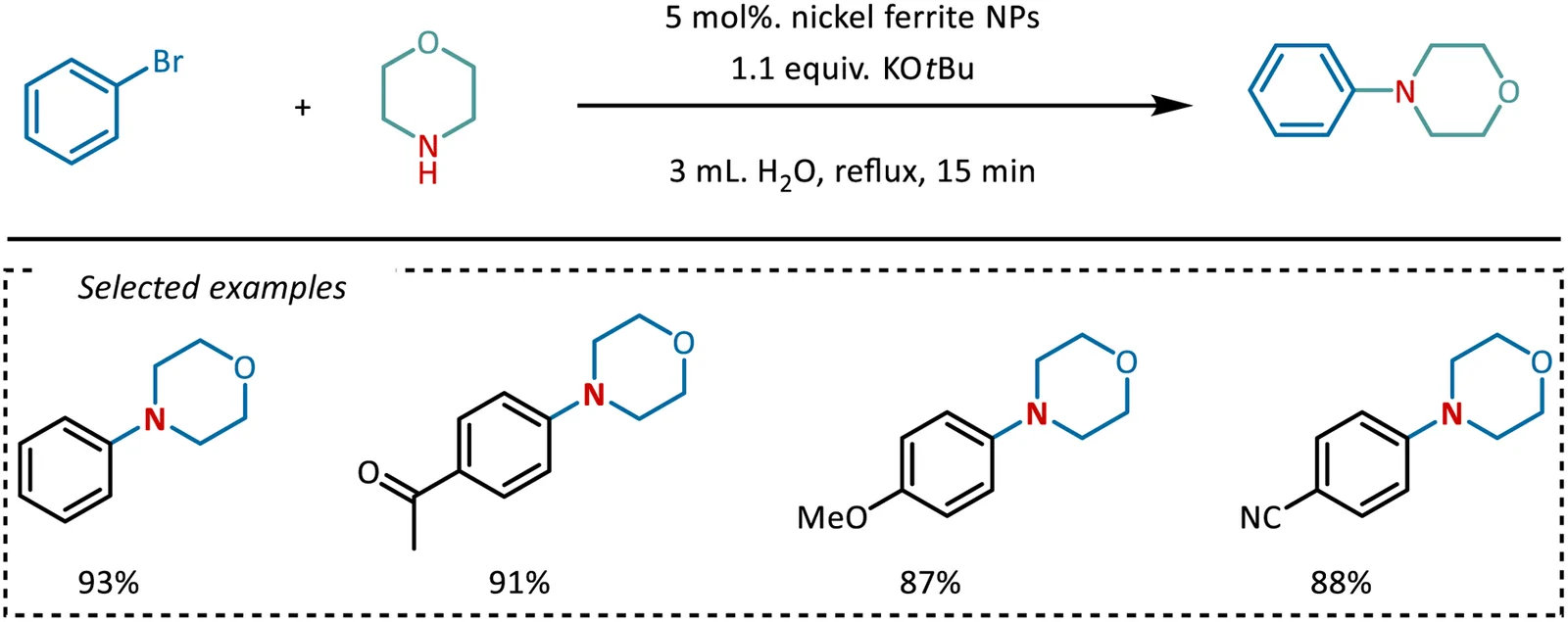
Nickel ferrite nanoparticle-catalysed C–N bond formation in water.

A mechanistically distinct nickel-based approach was reported by Schäfer and co-workers, who demonstrated the reductive amination of aldehydes and ketones with amines in water using Ni–Al alloy. The reaction proceeds through imine formation, followed by hydrogenation over Raney-type nickel generated *in situ* from the interaction of Ni–Al alloy with water. Thus, water not only acts as the solvent but also participates indirectly in hydrogen generation through the Al–H_2_O process. Ultrasonic activation improves the catalyst surface and likely enhances hydrogen formation, enabling efficient reductive C–N bond construction under aqueous conditions (Scheme 41).^128^

**Scheme 41:**
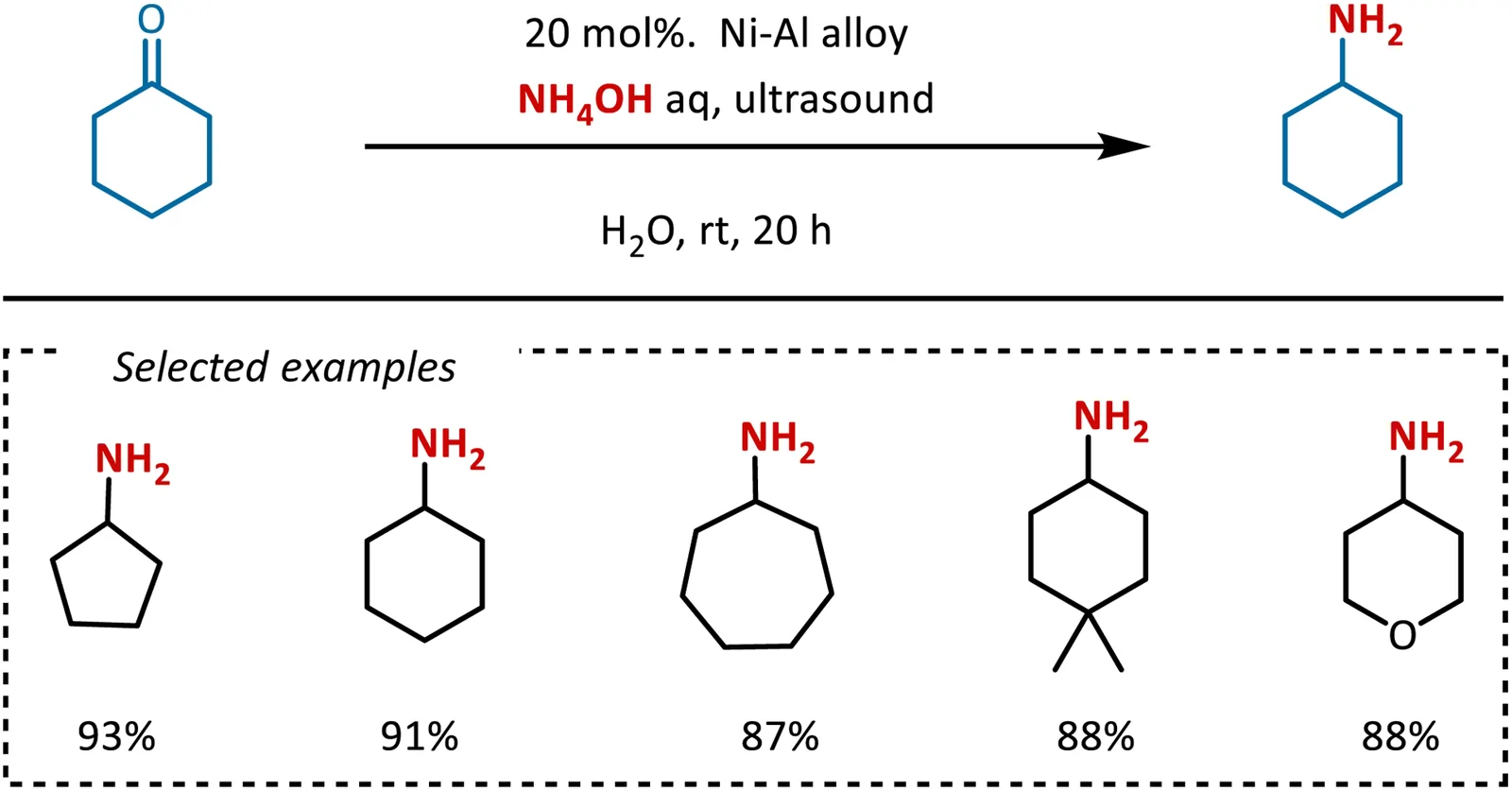
Heterogeneous catalytic reductive amination of carbonyl compounds with Ni–Al alloy in water.

### 3.8 Other metal-catalysed C–N bond formation in water

Beyond the dominant Cu-, Pd-, Ir-, Rh-, Fe-, Au-, and Ni-based catalytic platforms, several less-explored metal systems further demonstrate the versatility of water as a medium for C–N bond construction.

Rose and co-workers reported the first heterogeneous Ru/C-catalysed amination of biogenic isohexides, including isosorbide, isomannide, and isoidide, in aqueous ammonia. This transformation converts renewable sugar-derived diols into valuable amino alcohols and diamine monomers through a hydrogen autotransfer (borrowing-hydrogen) pathway. Mechanistically, the secondary alcohol undergoes dehydrogenation to generate a carbonyl intermediate, followed by condensation with ammonia to form an imine species and subsequent hydrogenation to furnish the corresponding amine products. The use of aqueous ammonia as both a nitrogen source and reaction medium highlights the potential of water-based hydrogen-transfer processes for the sustainable synthesis of nitrogen-containing building blocks.^129^ Pt/C has also been applied for the microwave-assisted upgrading of primary amines to secondary amines in water through sequential imine formation and reductive amination pathways.^130^

Cobalt catalysis provides an alternative approach using a more abundant metal platform. Teo and co-workers developed a Co(C_2_O_4_)·2H_2_O/dmeda-catalysed *N*-arylation of benzamides with aryl iodides in water, affording *N*-aryl products with high selectivity and yields. Compared with conventional precious-metal-based cross-coupling systems, cobalt catalysis offers advantages in terms of cost and resource availability, although broad substrate applicability and mechanistic understanding remain areas for further development.^131^

Silver catalysis has further extended aqueous C–N bond formation to nitrene-transfer chemistry. Schomaker and co-workers demonstrated that water-compatible nitrene precursors could enable selective Ag-catalysed C–H amination in aqueous media while maintaining useful chemo- and site-selectivity. This study is particularly significant because it shows that highly reactive metal–nitrene intermediates can be generated and controlled under aqueous conditions.^132^

Collectively, these emerging metal-based systems demonstrate that aqueous C–N bond formation is not restricted to established Cu, Pd, Rh, Ir, Fe, Au, and Ni platforms. Instead, water can support diverse catalytic mechanisms by facilitating hydrogen-transfer processes, stabilizing reactive intermediates, improving substrate organization, and enabling environmentally preferable reaction conditions. Nevertheless, these approaches remain at an early stage compared with mainstream aqueous catalytic systems, and challenges related to catalyst cost, substrate scope, reaction efficiency, additive dependence, and scalability must be addressed before broader synthetic and industrial applications can be realized.

Table 1 provides a comparative overview of representative metal-catalysed C–N bond-forming strategies in water, highlighting the diversity of catalytic platforms, reaction types, advantages, limitations, and mechanistic contributions of water.^16,24^ The comparison demonstrates that water can influence different catalytic systems through distinct effects, including catalyst stabilization, interfacial activation, proton transfer, substrate organization, and hydrogen transfer. While earth-abundant metals, such as Cu, Fe, and Ni, offer advantages in terms of cost and sustainability, noble-metal catalysts, including Pd, Rh, Ir, and Au, provide unique reactivity and selectivity. Nevertheless, challenges related to catalyst cost, substrate scope, additive requirements, and scalability remain important considerations for the future development of aqueous C–N bond-forming methodologies.

**Table 1:** Comparative summary of metal-catalyzed C–N bond formation in water

Metal	Typical reactions	Advantages	Limitations	Representative role of water^a^
Cu	Ullmann-type cross-coupling, C–H functionalization, aerobic oxidation, and intramolecular *N*-arylation	Low cost, versatile, and amenable to micellar and heterogeneous systems	Requirement for ligands, need for strong bases, high temperature requirement, and limited scope for unactivated aryl chlorides	Solvent, Cu-species stabilizer, proton-transfer medium, and interfacial promoter
Pd	Buchwald–Hartwig amination, C–H amination, and cascade cyclization	Broad substrate scope, high functional-group tolerance, and high activity	High cost, specialized ligand requirement, and potential metal contamination	Solvent, micellar organizer, catalyst stabilization, and substrate dispersant
Ir	Transfer hydrogenation, borrowing-hydrogen amination, and reductive hydroamination	High selectivity, mild operating conditions, and water as hydrogen source	High cost, water-soluble ligand requirement, and limited substrate scope	pH regulator, proton-transfer medium, and hydrogen source
Rh	C–H amidation, C–H amination, nitrene transfer, annulation, and aminohydroxylation	High reactivity, broad C–H substrate scope, and regioselectivity	Precious metal status, requirement for oxidants/nitrene precursors, and necessity of directing groups	Interfacial activator, hydrogen-bonding promoter, proton-transfer mediator, and nucleophile source
Fe	*N*-Arylation, ammoxidation, C–H activation, and nitrile synthesis	Earth-abundance, low toxicity, and synthetic feasibility of heterogeneous single-atom catalysts	Limited reaction diversity and requirement for engineered catalysts	Substrate dispersion, Fe-site stabilization, and proton-transfer mediation
Au	Reductive amination, cascade hydrogen transfer, and quinazoline synthesis	Heterogeneous, recyclable, and operable under mild conditions	High cost, limited reaction scope, supported catalyst requirement	Green medium and hydrogen donor
Ni	*N*-Arylation and reductive amination	Earth-abundance, low cost, magnetic recoverability	Limited examples and requirement for specialized conditions	Solvent and hydrogen generation through Al–H_2_O process
Ru	Borrowing-hydrogen amination and reductive amination	Versatility and heterogeneous catalytic systems	Precious metal and limited examples	Solvent
Ag	Nitrene-transfer C–H amination	Reactive intermediates and mild conditions	High cost and limited scope	Solvent
I_2_ (metal-free)	Oxidative cyclization and C–H functionalization	No metal required, air as oxidant, and high atom economy	Limited examples	Solvent

a The role of water depends on the specific reaction system and should not be generalized across all catalytic processes. In some cases, its involvement has been experimentally demonstrated, whereas in others, it has been proposed based on mechanistic studies. Sustainability advantages should be evaluated using comprehensive green chemistry metrics, including atom economy, E-factor, and process mass intensity, rather than solvent selection alone.

## 4 Critical limitations and future challenges of metal-catalysed aqueous C–N bond formation

Despite significant advances, aqueous C–N bond-forming methodologies still face several substrate- and catalyst-dependent limitations. For example, Cu-catalysed Ullmann-type processes generally show reduced efficiency toward unactivated aryl chlorides and sterically demanding amines because of challenging oxidative addition and competitive coordination effects.^133,134^ Pd-based systems provide broad substrate tolerance but often rely on specialized ligands and face concerns associated with catalyst cost and residual metal contamination.^135^ Precious-metal catalysts, such as Rh and Ir, enable highly selective C–N bond construction but remain limited by catalyst expense, ligand requirements, and restricted substrate classes.^136^ Furthermore, complex multifunctional substrates may suffer from reduced chemoselectivity, catalyst inhibition, or instability of reactive intermediates under aqueous conditions. Heterogeneous and micellar aqueous systems offer advantages in catalyst recovery and substrate organization; however, challenges related to mass transfer, substrate solubility, catalyst stability, and scale-up remain important considerations.^137^

## 5 Electrochemical and photochemical C–N bond formation in water

Electrochemical C–N bond formation in water has recently emerged as a sustainable strategy for converting inorganic nitrogen sources into value-added organonitrogen compounds under mild conditions. In contrast to conventional thermal routes, electrosynthesis uses electricity as the redox input and water as the electrolyte medium, thereby enabling proton/electron transfer, ion transport, and *in situ* generation of reactive nitrogen intermediates.

Zhang and co-workers reported an aqueous pulsed-electrochemical strategy for the one-pot synthesis of primary arylamines from nitrite and arylboronic acids (Scheme 42a).^138^ In this system, nitrite is first reduced to ammonia on a low-coordinated Cu nanocoral cathode. During the anodic pulse, the same Cu electrode generates Cu(ii) species *in situ*, which then promote a Chan–Lam-type coupling between ammonia and arylboronic acids. Thus, water serves as the reaction medium for both nitrite reduction and subsequent C–N coupling, while the pulsed potential integrates heterogeneous electroreduction with homogeneous Cu-mediated amination without requiring an external copper salt.

**Scheme 42:**
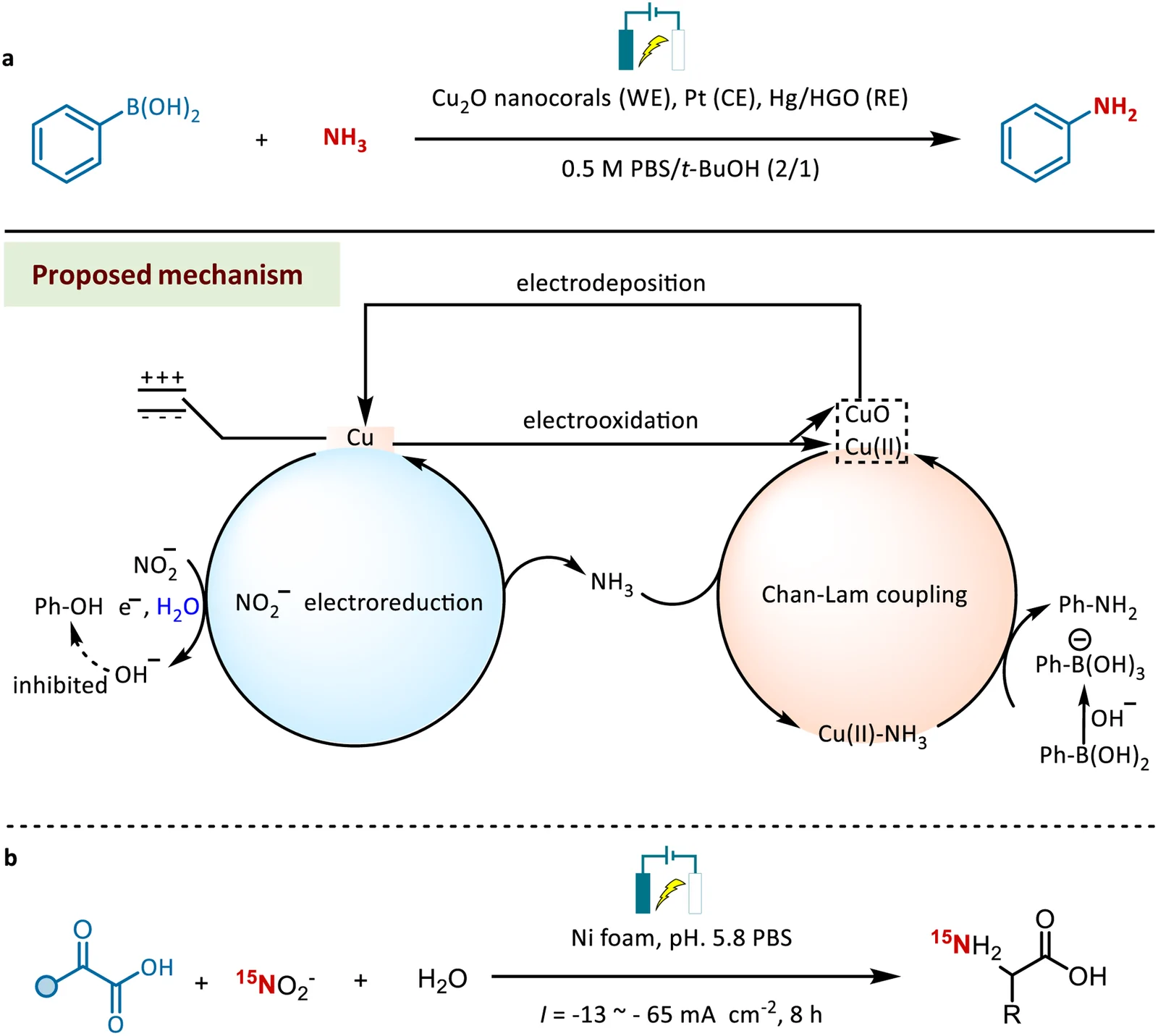
(a) Aqueous pulsed electrochemistry promotes C–N bond formation *via* a one-pot cascade approach. (b) Electrocatalytic C–N bond construction from inorganic nitrogen sources in water.

In a broader development, Zhang and co-workers, in 2026, described electrocatalytic C–N bond construction from inorganic nitrogen sources, including NO, NO_2_^−^, NO_3_^−^, and ^15^NO_2_^−^, in water under ambient conditions (Scheme 42b).^139^ This platform enables the synthesis of urea, formamide, cyclohexanone oxime, amino acids, and ^15^N-labelled amino acids. Mechanistically, electroreduction generates nucleophilic nitrogen intermediates, such as *NH_2_OH and *NH_2_, which couple with electrophilic carbon intermediates derived from CO_2_, formic acid, ketones, or keto acids. Water is essential as the electrolyte environment because it supports ion transport, proton/electron transfer, stabilization of charged intermediates, and the use of nitrogen oxides as recyclable nitrogen sources. However, the efficiency of these systems is still limited by competing hydrogen evolution, self-reduction of carbon or nitrogen intermediates, mismatched C/N intermediate formation rates, and challenges in long-term scale-up.

The electrochemical C–N bond formation in water expands aqueous synthesis beyond classical organic substrates by directly valorising inorganic nitrogen species into useful organonitrogen products. This strategy is particularly attractive for sustainable synthesis because it combines water, renewable electricity, and waste-derived nitrogen sources, although further improvements in faradaic efficiency, product scope, catalyst durability, and scalable reactor design remain necessary.

Visible-light-driven photochemistry has emerged as a useful strategy for C–N bond formation under mild conditions, particularly when combined with aqueous or partially aqueous reaction media. In these systems, water may serve as a green medium, a proton-transfer environment, or a participant in downstream hydrolysis steps, while light enables single-electron-transfer pathways or metal-catalysed cross-coupling under ambient conditions.^140^

Akita and co-workers reported an intermolecular aminotrifluoromethylation of alkenes using Ru(bpy)_3_^2+^ as a photocatalyst under blue LED irradiation in an aqueous MeCN system (Scheme 43).^141^

**Scheme 43:**
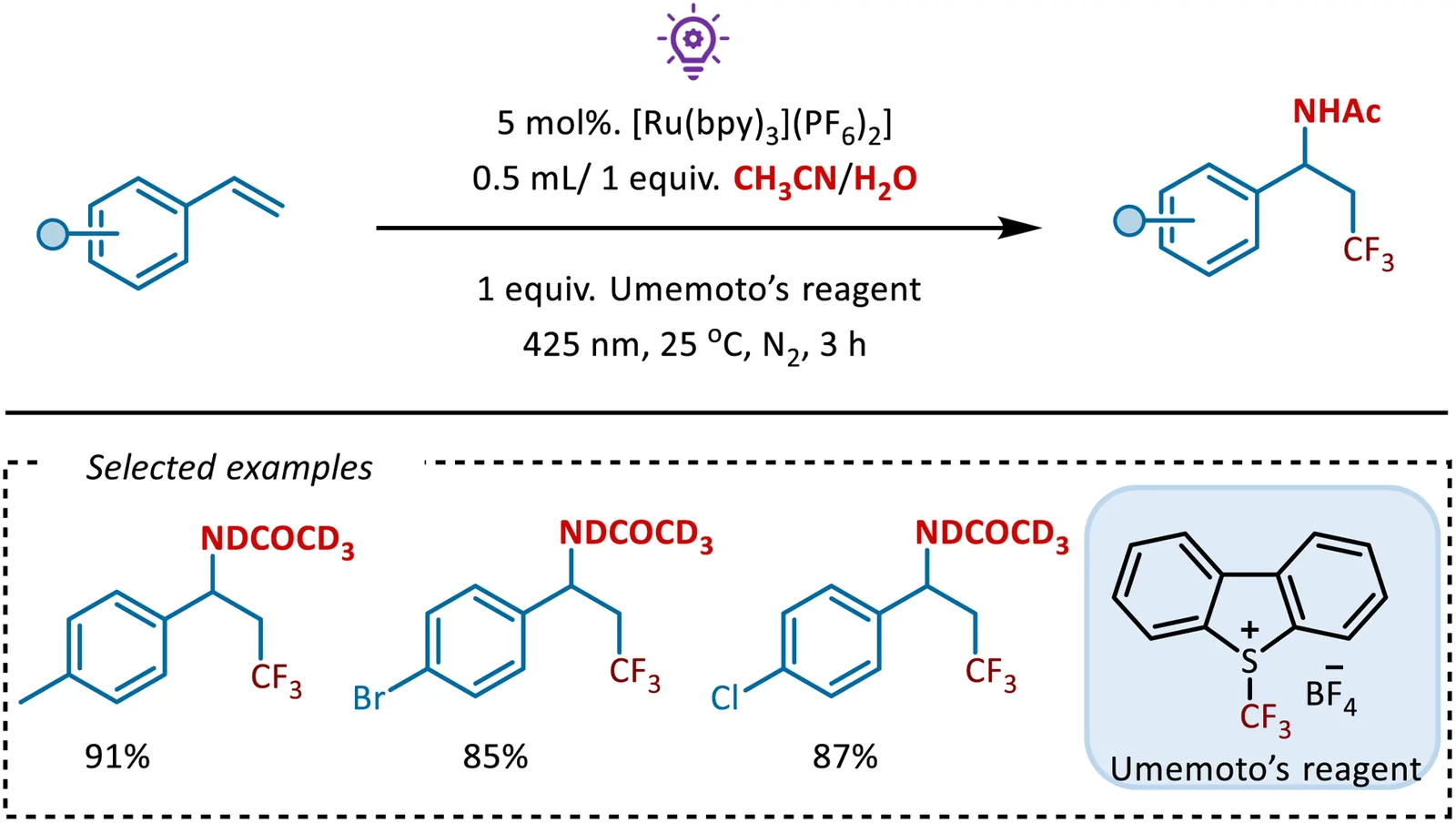
Intermolecular aminotrifluoromethylation of alkenes by visible-light-driven photoredox catalysis.

In this transformation, Umemoto's reagent generates a CF_3_ radical through single-electron transfer, followed by radical addition to styrenyl alkenes to form a β-trifluoromethylated radical/carbocation intermediate. MeCN then acts as the nitrile nucleophile in a Ritter-type C–N bond-forming step, while water may assist the subsequent hydrolysis to afford β-CF_3_ amide products. Although this reaction is not performed in pure water, the MeCN/H_2_O medium is essential because MeCN provides the nitrogen source and water enables the hydrolytic conversion of the nitrilium-type intermediate into the final amide.

A more direct example of photochemical C–N cross-coupling in water was reported by Xia and co-workers, who developed a visible-light-mediated Ni(ii)-catalysed coupling of arylamine-derived picolinamides with pyrazoles (Scheme 44).^142^ Using NiSO_4_·6H_2_O as the nickel catalyst, Acr^+^-MesClO_4_^−^ as the photocatalyst, and H_2_O_2_ as the oxidant under blue LED irradiation, the reaction proceeds at room temperature under air to give pyrazole-containing products with good regioselectivity. Here, water serves as a benign reaction medium that supports the nickel/photoredox catalytic system, avoids toxic organic solvents, and enables C–N bond formation under mild oxidative conditions.

**Scheme 44:**
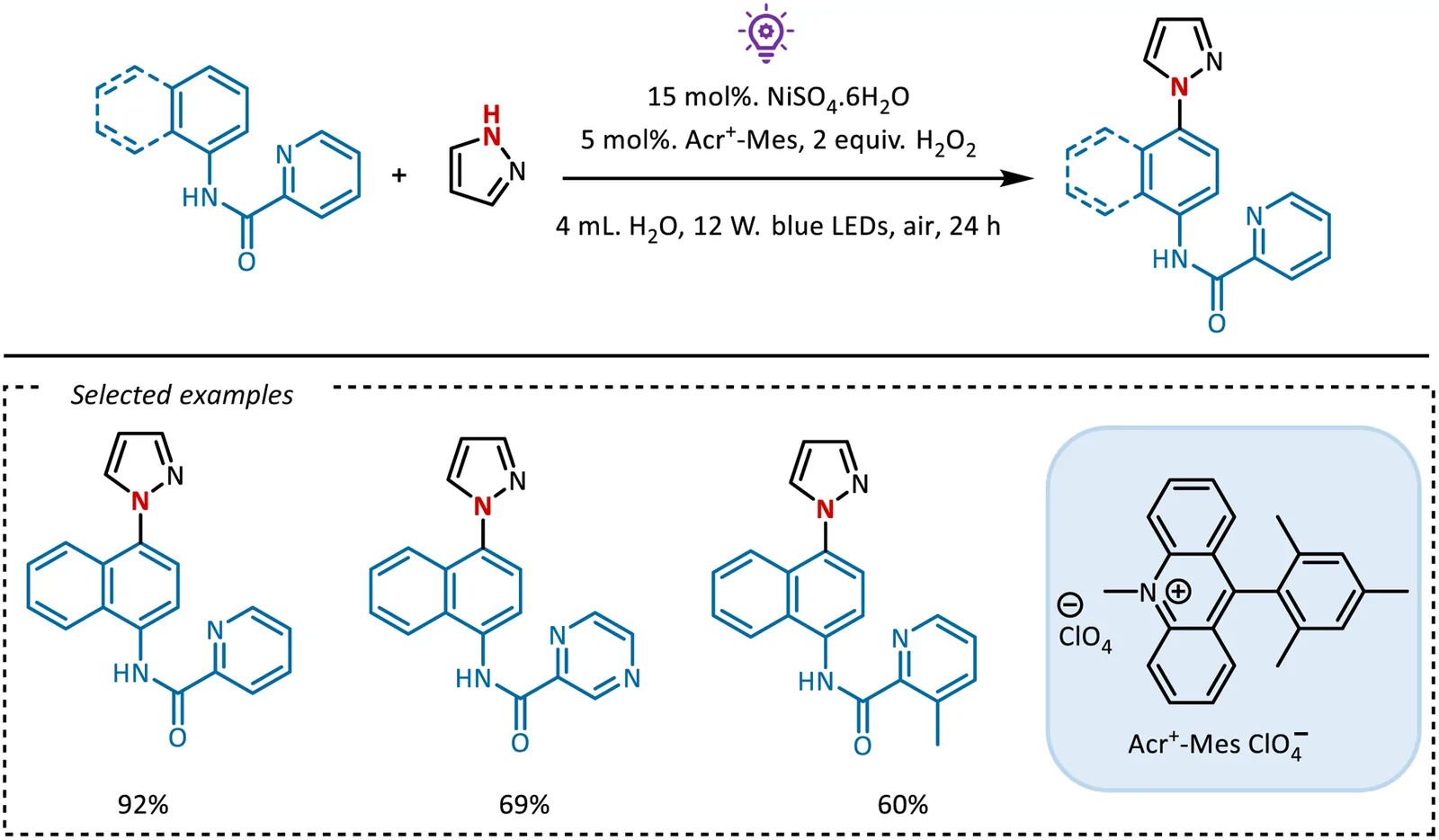
Visible-light-mediated Ni(ii)-catalysed C–N cross-coupling in water.

The photochemical C–N bond formation in aqueous media remains less developed than metal-free, Cu-, or Pd-catalysed aqueous strategies, but these examples highlight its potential for sustainable synthesis. Water can participate as a hydrolysis medium, proton-transfer environment, or green solvent for photocatalytic cross-coupling. Future development should focus on expanding truly water-based photochemical C–N coupling, reducing reliance on organic co-solvents, improving substrate scope, and clarifying how water influences radical, carbocationic, and metal-catalysed photoredox pathways.

## 6 Future perspective

The future development of aqueous C–N bond formation will require the integration of advanced catalyst design, mechanistic understanding, and process engineering. Artificial intelligence (AI)-guided reaction prediction combined with high-throughput experimentation could accelerate the discovery of efficient catalysts, optimized reaction conditions, and substrate water compatibility patterns in complex aqueous systems. Continuous-flow technologies represent another promising direction by improving mass transfer, heat management, catalyst recycling, and scalability compared to conventional batch processes. Furthermore, the integration of chemical catalysis with emerging biocatalytic strategies may provide complementary approaches for highly selective C–N bond construction under mild conditions, particularly for complex multifunctional molecules.

Beyond reaction development, successful industrial translation requires the consideration of process-related factors, including catalyst recovery, reaction concentration, energy efficiency, downstream processing, and regulatory requirements. Heterogeneous catalysts, recyclable nanocatalysts, and micellar catalytic platforms offer potential advantages through simplified separation, reduced organic solvent consumption, and improved catalyst reuse. However, challenges associated with dilute reaction conditions, substrate solubility, additive requirements, long-term catalyst stability, aqueous waste management, and residual metal control remain significant barriers. For pharmaceutical applications, compliance with regulatory standards, such as the ICH Q3D guideline for elemental impurities, is essential.

Therefore, future efforts should focus on developing concentrated aqueous reaction systems, recyclable catalyst architectures, continuous-flow processes, and renewable feedstock-based methodologies. The incorporation of biomass-derived substrates, CO_2_-derived intermediates, and nitrogen-containing waste streams could further enhance the sustainability of aqueous C–N bond formation. Importantly, quantitative evaluation using green chemistry metrics, including process mass intensity (PMI), E-factor, and atom economy, will be critical for assessing the overall environmental performance of these methodologies. Collectively, these advances will facilitate the transition of water-enabled C–N bond-forming reactions from laboratory-scale demonstrations toward practically viable and sustainable synthetic technologies.

## 7 Conclusion

This review has surveyed the rapid evolution of C–N bond formation in water, highlighting a paradigm shift from viewing water as a passive solvent to recognizing it as an active promoter of sustainable synthesis. Across transition-metal-free, transition-metal-catalysed, electrochemical, and photochemical strategies, water plays remarkably diverse mechanistic roles, such as a hydrogen-bond donor/acceptor, proton shuttle, interfacial activator, micellar organizer, polar-intermediate stabilizer, and even direct reactant or hydrogen source. Transition-metal-free systems exploit iodine-mediated oxidative cyclization and dual hydrogen-bond activation, while Cu and Pd catalysis have been successfully translated into aqueous micellar and heterogeneous platforms. Precious-metal catalysts (Ir, Rh) enable hydrogen-transfer and nitrene-transfer C–N bond formation in water. Fe- and Ni-based catalysts provide earth-abundant and cost-effective alternatives to noble-metal catalysts, whereas Au-based systems offer complementary advantages arising from their unique catalytic properties and selectivity. Electrochemical and photochemical approaches further expand the toolbox using renewable electricity or visible light to drive C–N coupling directly from inorganic nitrogen sources or under mild photo-redox conditions. Despite this progress, challenges remain: improving catalyst recyclability, replacing stoichiometric oxidants, broadening substrate scope to unactivated halides and complex amines, and scaling aqueous processes. Future directions should focus on integrating renewable feedstocks, developing fully recyclable catalytic systems, and deepening the mechanistic understanding of water's role at the molecular level. Ultimately, water is a powerful enabler of green, selective, and industrially relevant C–N bond construction.

## Conflicts of interest

There are no conflicts to declare.

## Data Availability

No primary research results, software or code have been included and no new data were generated or analysed as part of this review.
